# Can Gas Absorption be Tuned in a Multifunctional Ionic Liquid?

**DOI:** 10.1002/cssc.202501347

**Published:** 2025-09-02

**Authors:** Frederik Philippi, Margarida Costa Gomes

**Affiliations:** ^1^ Laboratoire de Chimie ENS de Lyon and CNRS Lyon 69364 France

**Keywords:** biogas upgrading, carbon capture, gas separation, porous ionic liquids, sustainability

## Abstract

The challenge of CO_2_ separation and management in biogas upgrading processes is addressed, which remains a critical bottleneck when considering biomethane as a competitive and sustainable alternative to natural gas. Ionic liquids offer a promising alternative to existing sorbents due to their negligible volatility and their tunable properties. Herein, a multifunctional phosphonium triazolate ionic liquid capable of reacting reversibly with CO_2_ without loss of fluidity through both cation and anion is presented. Using a combination of experiments and reaction models the interplay of different absorption mechanisms is demonstrated at varying temperatures and pressures, which lead to high capacity for CO_2_ absorption and excellent selectivity for CO_2_ over CH_4_. The multifunctional phosphonium triazolate can be used to prepare a porous ionic liquid with enhanced physical gas absorption by dispersing up to 10% w/w of ZIF‐8. The stability and porosity are maintained after CH_4_ absorption but are lost upon prolonged exposure to CO_2_ due to dissolution of the porous solid. These findings provide crucial insights for the development and modeling of ionic liquid‐based absorbents, paving the way for biogas upgrading technologies with reduced carbon footprint.

## Graphical abstract

A multifunctional phosphonium triazolate ionic liquid is capable of reversibly absorbing CO_2_ at high capacity while decreasing viscosity. The addition of ZIF‐8 leads to a porous ionic liquid with enhanced physical absorption, however, an unexpected loss of porosity occurs in the presence of CO_2_.

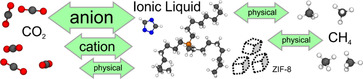

## 1 Introduction

Methane obtained from purified biogas is a renewable alternative to natural gas.^[^ ^1^ ^]^ In some locations, this biomethane is already fed into an existing commercial methane grid or used to supply small industrial, agricultural, and domestic networks.^[^ ^2^ ^]^ However, biogas purification currently presents two major challenges. First, biogas is a multicomponent mixture of gases present in widely different concentrations. This includes variable amounts of water as well as a number of other gases, some of which have physical properties similar to methane. Carbon dioxide typically accounts for around 50% of the mixture, while other components present in much lower concentrations include toxic and corrosive substances such as hydrogen sulphide (H_2_S) and ammonia (NH_3_).^[^ ^3^ ^]^ The second challenge lies in managing the relatively large amounts of separated carbon dioxide. Capturing the CO_2_ would further improve the carbon footprint of the process.^[^ ^4^, ^5^ ^]^

The first challenge is currently addressed using well‐established absorption and membrane‐based technologies.^[^ ^6^, ^7^, ^8^, ^9^ ^]^ Conventional CO_2_ absorption relies on aqueous amine solutions to chemically trap the gas, which is subsequently released by stripping at high temperatures.^[^ ^9^, ^10^ ^]^ These low‐cost absorbents show high reactivity and good capacity but also present serious drawbacks such as corrosion, degradation and solvent loss through evaporation.^[^ ^9^ ^]^ The main limitation however remains the high energy demand due to the large enthalpy of reaction with CO_2_ and the need to heat and evaporate significant amounts of water during regeneration. The second challenge – managing the separated carbon dioxide – is, in most cases, not presently addressed, and the gas is just released to the atmosphere.^[^ ^4^ ^]^ In very few cases the CO_2_ is collected for eventual utilization or valorization. Such strategies remain however, limited in scale and are not yet widely implemented in standard biogas upgrading systems.

In this work, we propose to address the two challenges in current technologies for biogas purification through the design of appropriate liquid absorbents. A key requirement for alternative liquid absorbents is negligible volatility to avoid losses and emissions when contacting with large volumes of gas over time. A second key requirement is a low environmental impact compared to existing technologies, including the avoided emissions of CO_2_ themselves. Finally, the safety of the working fluid itself is critical if small, decentralized biogas purification units are to be used widely. Ionic liquids – liquids composed solely of ions – are a large group of solvents with virtually unlimited design possibilities that can be adapted to fulfill these requirements.

**Figure 1:**
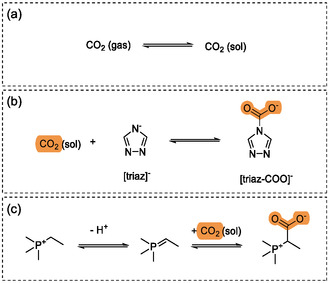
CO_2_ absorption mechanisms in an ionic liquid. a) Physisorption, b) anion reactivity, c) cation reactivity.

Ionic liquids, like any liquid, physically absorb CO_2_ (and other gases) as indicated in **Figure** **1**a. At low pressures, that is, low concentrations *c* of CO_2_, Henry's law can be used to describe the physisorption, Equation (1). Here, *p* is the partial pressure of CO_2_ above the liquid and $K_{H}$ is the Henry's law volatility constant.^[^ ^11^ ^]^ Typical values of $K_{H}$ for CO_2_ in ionic liquids range from 10 to 100 bar corresponding to mole fraction concentrations of CO_2_ around 10^−2^ at ambient temperature and 1 bar CO_2_.^[^ ^12^, ^13^ ^]^ Even though recycling ionic liquids is relatively straightforward using conventional pressure or temperature swing processes, their physical absorption capacities are too low to consider them viable alternatives as absorbents for biogas purification. Much higher concentrations can be achieved either by increasing the free volume in the ionic liquid or by making it react with CO_2_.

(1) $$K_{H}=\underset{\left[{\text{CO}}_{2}\right]\to 0}{\lim}\left(\frac{c\left({\text{CO}}_{2}\left(\text{gas}\right)\right)}{c\left({\text{CO}}_{2}\left(\text{sol}\right)\right)}\right)\approx \frac{p}{c\left({\text{CO}}_{2}\left(\text{sol}\right)\right)}$$

An effective way of increasing the free volume is to disperse a porous solid, such as a zeolitic imidazolate framework (ZIF), in a suitable ionic liquid which does not occupy the pores. This results in a porous ionic liquid with enhanced physisorption for small molecules such as CH_4_ or CO_2_,^[^ ^14^, ^15^ ^]^ compared to the pure ionic liquid.

When it comes to promoting the chemical reaction between carbon dioxide and the ionic liquid, consequently increasing the gas absorption capacities, many alternatives have been considered. Initially, efforts focused on the functionalization of ions to mimic known reaction pathways^[^ ^16^ ^]^ while more recently, new families of ions with tailored physicochemical properties have been proposed.^[^ ^17^ ^]^ An example of such reactions is the (reversible) formation of carbamates from aprotic heterocyclic anions, such as the 1,2,4‐triazolate anion shown in Figure 1b.^[^ ^17^, ^18^, ^19^ ^]^ Similarly, in some cases, the cation reacts with CO_2_ as reported for phosphonium‐ or imidazolium‐based ionic liquids that reversibly form carboxylate zwitterions.^[^ ^20^, ^21^, ^22^ ^]^ Typically, the loss of a proton is involved, requiring a sufficiently basic environment for the reaction to proceed. To this end, cations such as phosphonium or imidazolium are paired with basic anions such as acetate, which can act as proton scavengers. The resulting ionic liquid is able to react with CO_2_ when the anion's basicity is sufficient. In contrast, phosphonium or imidazolium ionic liquids formed by weakly coordinating anions such as bistriflimide generally do not react with CO_2_ and show only physisorption.

In this work, we address both strategies in the design of a new CO_2_ absorbent^[^ ^23^ ^]^ – increasing free volume and promoting gas chemisorption while preserving other favorable properties – by seeking to design, for the first time, a porous ionic liquid capable of reacting with carbon dioxide through both its cation and anion. We focus on ionic liquids based on phosphonium cations and basic anions, which are known to react with CO_2_ in the cation via an ylide/ylene mechanism, following a 1:2 stoichiometric ratio and leading to a zwitterion as shown in Figure 1c.^[^ ^20^, ^24^ ^]^ When the phosphonium cations are associated with basic triazolate anions, carbon dioxide reacts mainly with the anion following a 1:1 stoichiometric ratio at temperatures close to ambient.^[^ ^18^ ^]^ We explore herein the possibility of controlling the thermodynamics of these reactions to modulate gas uptake via both the cation and the anion, depending on the temperature and/or gas partial pressure. We anticipate that this system can meet all the key requirements for biogas upgrading as thus holds strong potential for this application.

## 2 Experimental Section

### 2.1 Materials

**Figure 2:**
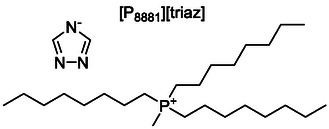
The molecular structure of the trioctylmethylphosphonium 1,2,4‐triazolate ionic liquid used in this work.

The ionic liquid tributylmethylphosphonium 1,2,4‐triazolate, [P_4441_][triaz], was prepared from commercially available precursors, tributylmethylphosphonium methylcarbonate, [P_4441_][MeCO_3_], and triazole, as described in Supporting Information (SI). For the preparation of methyltrioctylphosphonium 1,2,4‐triazolate, [P_8881_][triaz] represented in **Figure** **2**, the commercially available precursors [P_8881_][MeCO_3_] and triazole were used. Details of the synthesis and characterization (NMR, thermal transitions, density, viscosity, heat capacity, diffusion coefficients, X‐ray scattering) can be found in the Supporting Information. [P_8881_][triaz] was obtained in 98% purity based on ^31^P NMR, consistent with the purity of the [P_8881_][MeCO_3_] precursor. This approach was advantageous compared to the typical synthesis route for the more commonly known [P_66614_][triaz], since it avoids the intermediate halide and the ion exchange step.^[^ ^25^, ^26^, ^27^ ^]^

ZIF‐8 (BASF Basolite Z1200 obtained through Sigma‐Aldrich, Lot number STBG5528V) powder was passed through an 11 μm Nitex nylon mesh (1 h sieving, yield 480 mg) and dried for 5 h at 80 °C under dynamic rotary vane pump vacuum.

Carbon dioxide, CO_2_ 4.5, and methane, CH_4_ 4.5, were purchased from Air Liquide with a mole fraction purity of 99.995%. Both gases were used as received.

### 2.2 Gas Absorption Measurements

Gas absorption was measured using an IGA‐001 Intelligent Gravimetric Analyzer (Hiden Isochema) following a previously described procedure; full details are provided in the Supporting Information.^[^ ^15^ ^]^ The density of the samples was necessary for the calculation of the quantity of gas absorbed by the different liquid samples, and so it was measured using a combined viscometer/densitometer (SVM 3001 from Anton Paar) as described in the Supporting Information.

### 2.3 NMR Measurements

The ^1^H, ^13^C, and ^31^P NMR spectra in Figure 4 were collected at room temperature on a Bruker Avance III 400 MHz spectrometer equipped with a 5 mm CryoProbe Prodigy probe. The ionic liquid was degassed under a primary vacuum in a glass NMR tube having 0.38 mm glass wall thickness and an external diameter of 5 mm. Prior to degassing, a sealed glass capillary containing deuterated dimethyl sulfoxide was inserted in the NMR high‐pressure tube to act as an internal reference. After recording the NMR spectra of the pure ionic liquid under vacuum, the liquid was kept at 30 °C or 70 °C under a CO_2_ pressure of ≈10 bar.

## 3 Results and Discussion

It is well known that ionic liquids with low molecular weights generally exhibit higher gas absorption per unit mass. However, these compounds often have higher melting points, which can limit their practical use. This trade‐off is evident in the case of the ionic liquid [P_4441_][triaz], which has a relatively low molecular weight but exhibits a prohibitively high melting point of 162 °C – far too high for applications such as biogas upgrading. As a result, we focused our investigation on [P_8881_][triaz] as an absorbent for CO_2_ and CH_4_.

**Figure 3:**
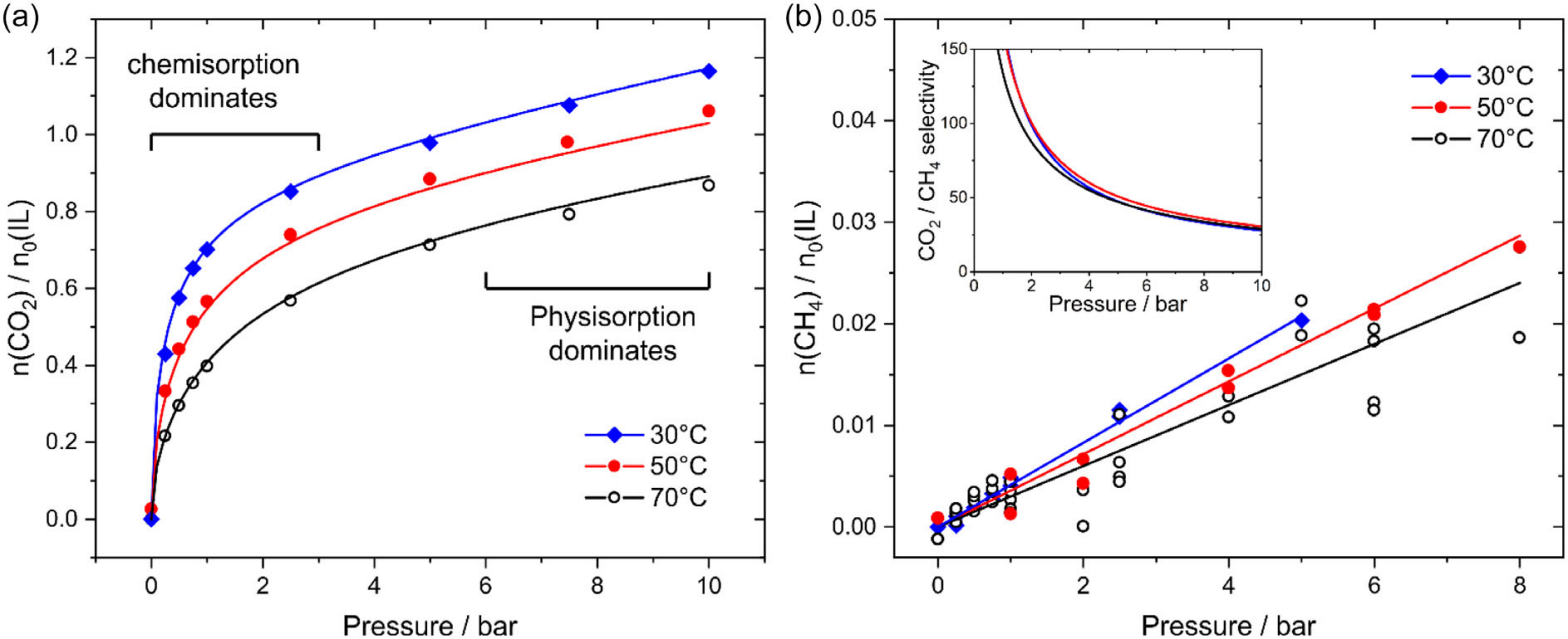
a) CO_2_ absorption isotherms for [P_8881_][triaz]. Symbols represent experimental data points and lines represent the single fit of all data points across the three isotherms. b) CH_4_ absorption isotherms for [P_8881_][triaz]. Symbols represent experimental data points, lines represent separate linear fits. The insert shows the ideal CO_2_/CH_4_ selectivity.

The CO_2_ and CH_4_ absorption in [P_8881_][triaz] are presented in **Figure** **3** and reported in the Supporting Information. The isotherms shown in Figure 3a reveal that CO_**2**_ absorption capacity in [P_8881_][triaz] does not increase linearly with pressure, suggesting the gas is chemically absorbed. Chemisorption seems to quickly saturate at gas pressures around 1 bar, while data at higher pressures mainly corresponding to the physisorption of the gas.

In contrast, Figure 3b clearly shows that CH_4_ does not react with the ionic liquid, its concentration increasing linearly with pressure, following Henry's law. Based on the ratio of gas concentrations in the ionic liquid, we calculated the selectivity of [P_8881_][triaz] for CO_2_ as shown in the insert of Figure 3b. This selectivity exceeds 150 at low pressures, where CO_2_ chemisorption dominates, and gradually decreases to a value of around 30 at gas partial pressure of 10 bar.

**Figure 4:**
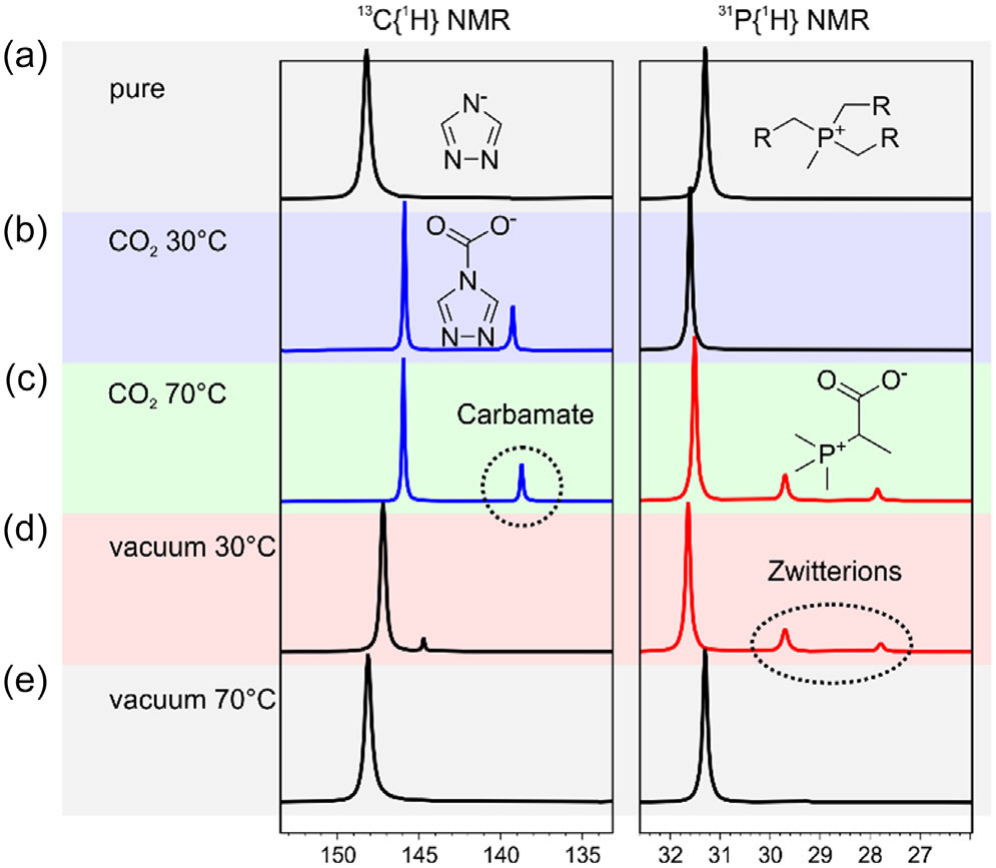
Subsequent NMR experiments on neat [P_8881_][triaz], from top to bottom: a) initial ^13^C{^1^H} and ^31^P{^1^H} signals for anion and cation, respectively; b) After pressurizing with CO_2_ at 10 bar, measured within a few hours; c) After heating to 70 °C until no further changes were observed (^31^P{^1^H} integral ratio ≈25:5:2); d) after degassing in vacuum at 30 °C; e) after degassing in vacuum at 70 °C.

To identify the products of the chemical reaction with CO_2_, we used NMR spectroscopy and conducted a series of experiments with [P_8881_][triaz] exposed to pressurized CO_2_ inside a gas‐tight NMR tube. While temperature and pressure could only be controlled in the tube prior to the NMR measurement (as the sealed tube had to be transferred into the magnet and analyzed at room temperature), and CO_2_ diffusion through the viscous ionic liquid was relatively slow, the chemical insights gained from these semi‐quantitative experiments, illustrated in **Figure** **4**, are the basis for interpreting our gas absorption data.

In the absence of CO_2_, the anion and the cation signals were identified at *δ* = 148.2 ppm in the ^13^C{^1^H} NMR and *δ* = 31.3 ppm in the ^31^P{^1^H} NMR, respectively (Figure 4a). After evacuating and pressurizing the tube to 10 bar of CO_2_ at 30 °C during 3.5 h, the ^13^C{^1^H} NMR reveals a new peak at 139.3 ppm attributed to the carbamate species formed from the anion (Figure 4b) as well as an upfield shift to 145.9 ppm of the carbon of the triazole ring. This is the evidence of the reaction in Figure 1b. Under these conditions, the ^31^P NMR remains largely unchanged, showing only a slight downfield shift of the phosphonium peak to 31.6 ppm. At longer timescales, cation reactivity becomes apparent, see Figure S26 in the Supporting Information.

When the temperature was raised to 70 °C for 12 h, while keeping the CO_2_ pressure at 10 bar, additional signals appeared in the NMR spectra (Figure 4c), revealing a second chemisorption mechanism of the gas. Specifically, two new peaks were observed in the ^31^P{^1^H} NMR at 29.7 and 27.9 ppm, attributed to zwitterionic species formed on the octyl and methyl side chains of the phosphonium cation, respectively, evidence of the reaction in Figure 1c. Under these conditions, ≈20% of the ionic liquid cations were found to have reacted with CO_2_. This value remained unchanged even after extending the reaction time by two days, indicating that the NMR spectrum in Figure 4c corresponds to the equilibrated system.

The two dominant chemisorption mechanisms observed at different temperatures were also evident during gas desorption. After evacuating the NMR tube at 30 °C overnight, most of the carbamate species disappeared, as shown in the ^13^C{^1^H} NMR spectrum (Figure 4d), whereas the phosphorus‐containing reaction products remained unchanged (^31^P{^1^H} NMR, Figure 4d). As in Figure 4b, the system is not at equilibrium, since the reaction kinetics of the gas with the anion are much faster than those in the cation as proven when we leave the system at room temperature overnight and signals of the reaction with the cation appear here. After heating the sample to 70 °C overnight under vacuum, the chemisorbed CO_2_ was released from both cation and anion, and the original ionic liquid was restored as shown in Figure 4e.

Based on these NMR data, we can infer that the reaction of the cation with carbon dioxide (reaction in Figure 1c) proceeds preferentially at higher temperature, while the reaction of the anion with CO_2_ (reaction in Figure 1b) occurs over the temperature range covered. According to literature studies on similar ionic liquids,^[^ ^28^ ^]^ this difference is likely due to reaction kinetics. It has been observed that carbon dioxide reacts with the triazolate anion within minutes at 60 °C and 1 bar, while the reaction with a phosphonium cation takes longer to reach equilibrium–ranging from hours to days.

After identifying the chemical species involved in CO_2_ absorption and quantifying the total amount of gas absorbed, it is interesting to distinguish and quantify the chemical and physical contributions to the overall gas uptake by the liquid. This distinction enables a detailed thermodynamic characterization of the gas absorption mechanisms. By understanding the energetics associated with each process, insights into the driving forces and reversibility of gas absorption can be gained to inform strategies for efficient regeneration and recycling of the absorbent.

We assume that gas dissolution as a function of temperature follows Henry's law, despite the occurrence of chemical reactions in the liquid solution. In other words, we consider that Equation (1), where $K_{H}$ is the Henry's law constant, appropriately describes the physisorption process under the assumptions that both the gas and the solution behave ideally, and that the pressure and composition remain sufficiently low.

The dissolved CO_2_ can then react either with the anion toward carbamates or with the cation toward zwitterionic carboxylate species in a 1:2 stoichiometry. The corresponding equilibrium constants are $K_{A}$ for the anion, Equation (2), and $K_{C}$ for the cation, Equation (3). Here, the extents of reaction $\xi_{A}$ and $\xi_{C}$ are expressed in quantity of substance and are equivalent to the amount of carbamate and carboxylate formed, respectively. $n_{0}$ is the initial amount of ionic liquid used in the experiment. $K_{H}$ has units of pressure if concentrations are expressed in mole fraction or mole ratio, and takes different values in these two cases. $K_{A}$ and $K_{C}$ are dimensionless.

(2) $$K_{A}=\frac{\xi_{A}}{\frac{P}{K_{H}}\left(n_{0}-\xi_{A}-2\xi_{C}\right)}$$

(3) $$K_{C}=\frac{{\left(\xi_{C}\right)}^{2}}{\frac{P}{K_{H}}{\left(n_{0}-\xi_{A}-2\xi_{C}\right)}^{2}}$$

The change in mass of the sample directly measured experimentally results then from the total amount of absorbed CO_2_, Equation (4).

(4) $$n_{{\text{CO}}_{2}}=n_{{\text{CO}}_{2}\left(\text{sol}\right)}+\xi_{A}+\xi_{C}$$

Equation (2) and (3) must be solved for $\xi_{A}$ and $\xi_{C}$ and the resulting expressions substituted into Equation (4). The experimental gas absorption isotherms were then fitted to the resulting symbolic expression using a custom Python script as detailed in the Supporting Information. The curves obtained when fitting the molar ratio $n_{{\text{CO}}_{2}}/n_{0}$ are shown in Figure 3a (mole fraction or molality of absorbed gas can also be used as described in the Supporting Information). The effect of temperature was accounted for by substituting the $K_{H}$, $K_{A}$ and $K_{C}$ with their temperature dependent equivalents from the integrated van't Hoff Equation (5), which allows for the calculation of the enthalpy of CO_2_ absorption ΔH.

(5) $$K=K_{T_{\text{ref}}}\exp\left(\frac{\Delta H}{R}\left(\frac{1}{T_{\text{ref}}}-\frac{1}{T}\right)\right)$$

The enthalpy of physisorption of the gas determined herein is −13 kJ mol^−1^, which is consistent with previously reported values for the physical dissolution of CO_2_ in nonreactive ionic liquids.^[^ ^13^, ^20^, ^29^ ^]^ The reactions with cation or anion have similar experimental values for both enthalpy and entropy. Hence, the overall reaction enthalpy and entropy for the chemisorption of CO_2_ from the gas phase are ≈−37 kJ mol^−1^ and −118 J mol^−1^ K^−1^, respectively. For comparison, Gurkan et al. reported an overall $\Delta H=-43{\text{ kJ mol}}^{-1}$ and $\Delta S=-130{\text{ kJ mol}}^{-1}K^{-1}$ for the ionic liquid trihexyl(tetradecyl) phosphonium 2‐cyanopyrrolide [P_66614_][2‐CNpyr].

The absorption of CO_2_ in the ionic liquid is thus less exothermic than in the commonly used monoethanol amine (ΔH≈−80 kJ mol^−1^ at low loading).^[^ ^30^, ^31^ ^]^ This is advantageous since less energy is required to thermally regenerate the absorber. At 30 °C and 1 bar, we observe a capacity of ≈1.55 mmol CO_2_ for 1 g of [P_8881_][triaz]. Based on the reaction enthalpy, 57 J g^−1^ ionic liquid of thermal energy are required to desorb this amount of CO_2_, and an additional 85 J g^−1^ is required due to the heat capacity of the ionic liquid if the regeneration is performed at 70 °C (*cf.* Supporting Information).

**Figure 5:**
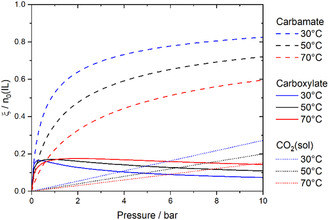
The distribution of the three relevant CO_2_ species as obtained from the fit. This distribution was not measured directly, it was obtained from the model chemistry and fitted parameters (equilibrium constants and enthalpies).

Our fit of the experimental isotherms also yields the extent of reaction of both anion and cation as helper variables, as shown in **Figure** **5**. In other words, $\xi_{A}$ and $\xi_{C}$ are quantities of carbamate and carboxylate formed as predicted using Equation (2) and (3), respectively. As no information about the chemical composition is used as input, the predicted $\xi_{A}$ and $\xi_{C}$ can deviate from their actual values if the model used to fit the experimental isotherm is incorrect or incomplete. However, the general trends are consistent with our NMR experiments. At 70 °C, the model predicts that ≈14% of the initially supplied cations have reacted to form the carboxylate species. This value is slightly lower than the ≈20% estimated from the ^31^P{^1^H} NMR data. Considering the limitations of NMR spectroscopy and the fact that the global fit does not include direct chemical speciation data, this level of agreement is remarkably good.

Our results reveal that physically absorbed CO_2_ becomes a significant contribution at intermediate pressures, particularly at lower temperatures. Notably, at 30 °C and around 3.8 bar, the amount of physically dissolved CO_2_ exceeds that bound as carboxylate, and a similar crossover is observed at ≈9.5 bar at 70 °C. These findings were not captured by Gohndrone et al.^[^ ^28^ ^]^ who did not explore pressures high enough to obtain sufficiently accurate data on the physisorption regime.

Gohndrone et al. previously investigated the competition between carbamate and carboxylate formation in [P_66614_][triaz] and found qualitatively similar reactivity to our system, [P_8881_][triaz]. However, their study focused primarily on low‐pressure conditions, while we adopted a comprehensive approach covering a wide pressure range, including the physisorption region. This broader scope allows us to extract deeper insights into the interplay of CO_2_ absorption using multifunctional ionic liquids.

For example, we observe that anion chemisorption ($\xi_{A}$) dominates across most of the studied pressure and temperature range. Nevertheless, at low CO_2_ pressures, the extent of cation carboxylation ($\xi_{C}$) can exceed that of anion chemisorption. The crossover point ($\xi_{C}=\xi_{A}$) shifts to higher pressures with increasing temperature and reaches about 0.6 bar at 70 °C which is consistent with the ≈0.6 bar crossover observed by Gohndrone et al.^[^ ^28^ ^]^ at 60 °C.

Furthermore, the extent of carboxylate formation ($\xi_{C}$) exhibits a maximum as a function of pressure due to competition with carbamate formation. This maximum shifts from ≈0.3 bar at 30 °C to ≈2 bar at 70 °C, indicating that at low pressures, $\xi_{C}$ decreases with increasing temperature—a trend also noted in the earlier literature. However, since the data reported by Gohndrone et al. only extend to pressures near the maxima observed in our study (*cf*. Figure 4 in reference),^[^ ^28^ ^]^ they could not observe the reversal of trends at higher pressures, where carboxylate formation increases with temperature while carbamate formation declines.

The reactivity of [P_8881_][triaz] and the competition between cation and anion reactivity can be fully explained by considering the “acidity” of the mixture. The proton released for every CO_2_ absorbed through the cation (Figure 1c) is captured by the basic species present in the mixture, specifically the anion. Critically, we observe an increase of the diffusion coefficients upon CO_2_ absorption, as listed in **Table** **1**. This suggests that the viscosity of the ionic liquid decreases during CO_2_ uptake, since fluidity strongly correlates with mass transport. In contrast, amine functionalization commonly leads to a significant increase in viscosity during CO_2_ uptake, sometimes by several orders of magnitude, which poses a serious challenge for liquid handling.^[^ ^16^, ^32^ ^]^ The viscosities of ionic liquid sorbents with aprotic heterocyclic anions such as triazolate are known to be less sensitive to CO_2_ absorption and can even decrease, like in our case.^[^ ^17^, ^19^ ^]^ A possible explanation for this effect is the introduction of neutral species, which act as diluent/momentum buffer, thus relaxing the constraints imposed by electroneutrality and accelerating bulk dynamics.^[^ ^33^ ^]^ Indeed, the acidic proton itself shows the fastest diffusion, and the increased fluidity has also been reported for phosphonium ionic liquids, which chemisorb exclusively via the cation reactivity.^[^ ^20^ ^]^ However, the fluidity of such a complex system with multiple species depends on a balance of several effects, and the exact behavior might change depending on the ionic liquid structure.

**Table 1:** Results of the fit in molar ratio representation, cf. Figure 5. The column labeled Physisorption reports the Henry solubility constant at 30 °C for physical absorption of CO_2_ as well as the enthalpy and entropy for this process. The two columns on the right contain the equilibrium constant, enthalpy and entropy for the chemical reaction of physically dissolved CO_2_ with anion or cation.

	Physisorptiona)	Anion reaction	Cation reaction
$K_{30\;{}^{\circ}C}$	0.027(2) bar^−1^	+112(10)	+27(11)
ΔH/kJ mol^−1^	−13(4)	−26(6)	−22(11)
ΔS/J mol^−1^ K^−1^	−72(14)	−47(20)	−45(37)

a) Using the Henry volatility constant with Equation (5) gives positive values for enthalpy and entropy, i.e., the desorption process; here we show the enthalpy and entropy of dissolution, which are consequently negative. The Henry solubility constant presented here corresponds to a Henry volatility constant of $K_{H}=37\left(3\right)\text{bar}$.

**Figure 6:**
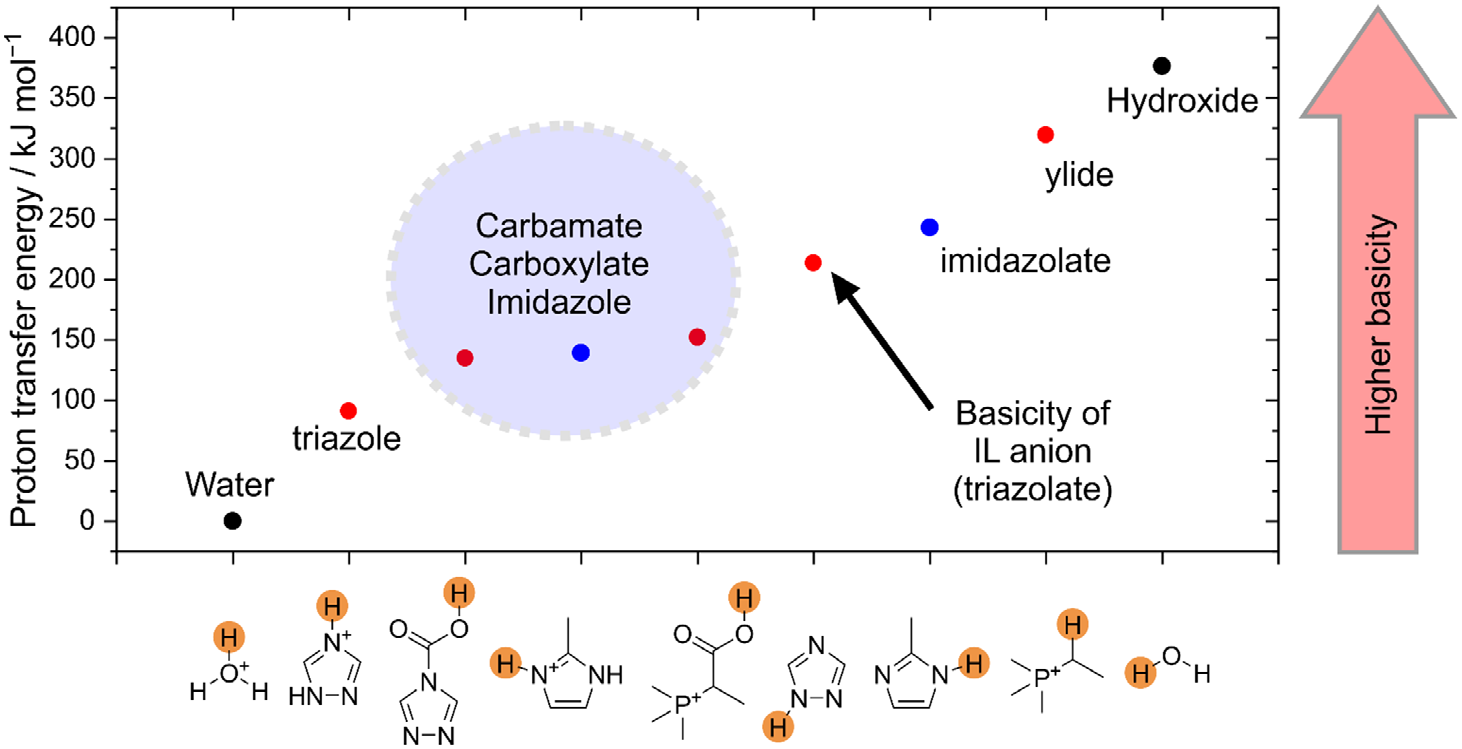
Energy required to transfer the proton highlighted in orange to H_2_O. The higher this energy, the higher the basicity of the corresponding base. The points in the graph are labeled with the names of the corresponding base. Carbamate, carboxylate, and imidazole are within 15 kJ mol^−1^ and can thus be considered equivalent in terms of basicity.

Although the large number of species in the reaction mixture complicates the chemical interpretation of our results, we decided to use the proton transfer energy as approximation to gauge the relative basicity of these species. To this end, we have calculated proton transfer energies of several protonated species as represented in **Figure** **6**. Details can be found in the Supporting Information. These deprotonation energies are conceptually similar to gas‐phase basicity and proton affinity, but were calculated in an implicit ionic liquid environment rather than the gas phase and without thermodynamic corrections. The quantitative values will thus change with the solvent considered and the explicit solvation environment.

Based on the proton transfer energy, the basicity of all considered species was between that of OH^−^ and H_2_O. The highest basicity was predicted for the ylide, which is expected given that it is considered as a fleeting species only present in low concentration. On the other end, the least basic (most acidic) species was triazole (triazolium), hence, protonation of triazole to triazolium can be neglected.

The basicity in neat [P_8881_][triaz] is determined by the triazolate anion. With increasing CO_2_ partial pressure, essentially all of the anions react. They can act either as proton scavengers in the carboxylate pathway (leading to triazole) or react directly with CO_2_ (leading to carbamate). The basicity in the neat ionic liquid after reaction with CO_2_ is thus determined by carbamate and carboxylate, with the liberated protons mostly localized on triazole. This observation is consistent with the experimental diffusion coefficients in **Table** **2**. The acidic protons show the fastest diffusion since they are located on the small and neutral triazole. The diffusion coefficient based on the anion/triazole signal is slightly lower since it is an average over four species: carbamate, triazole, and to a lesser degree remaining triazolate and protonated carbamate.

**Table 2:** Diffusion coefficients D/(10^−9^ cm^2^ s^−1^) which corresponds to D/(10^−13^ m^2^ s^−1^). All values were measured in the neat, dry ionic liquid at 30 °C using NMR diffusometry as described in the Supporting Information.

	Signal	Without CO_2_	With CO_2_
^1^H	Acidic proton	–	30
^1^H	Anion/triazole	8.4	26
^1^H	Alkyl	7.5	17
^31^P	Cation	7.6	17
^31^P	Carboxylate	–	14

**Figure 7:**
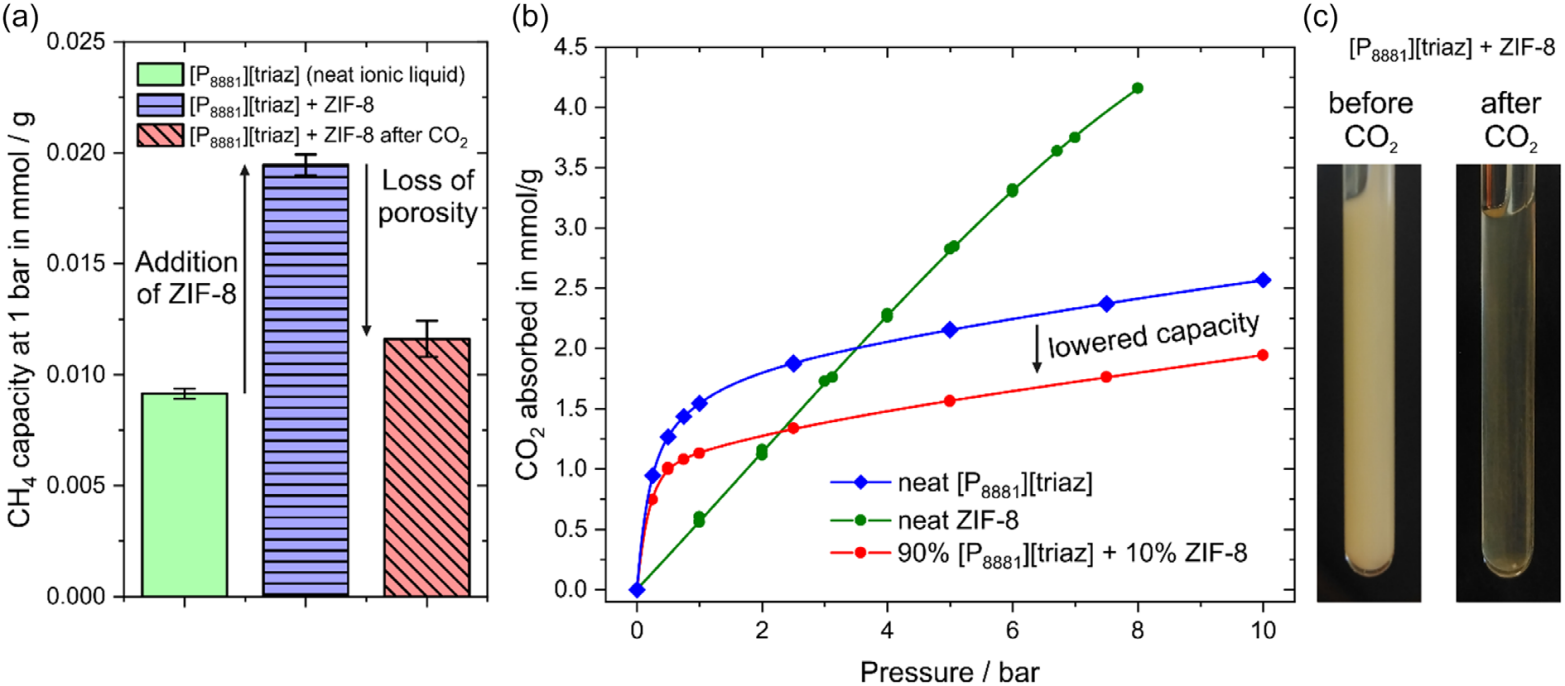
a) Increased physisorption capacity in the porous liquid and loss of porosity after treatment with CO_2_ and b) observed CO_2_ absorption isotherm at 30 °C for the mixture of [P_8881_][triaz] and ZIF‐8 as well as the two isolated components. Lines are a guide for the eye. c) Visual change after exposure to CO_2_.

Temperature, pressure, and time allow us to control the chemical speciation of CO_2_ in the ionic liquid. In order to tune the physisorption capacity of the ionic liquid, we decided to try to prepare porous [P_8881_][triaz] by dispersing 10% (w/w) of ZIF‐8 in the ionic liquid.^[^ ^14^, ^15^, ^34^ ^]^ Gas absorption measurements of CH_4_, which does not react chemically with the ionic liquid, confirmed that a porous liquid was obtained, since the absorption capacity of [P_8881_][triaz] containing ZIF‐8 was much higher than that of the neat ionic liquid, as depicted in **Figure** **7**a.

The CO_2_ absorption behavior was strikingly different as shown in Figure 7b. CO_2_ absorption is reported as molality due to the presence of a solid phase, the corresponding isotherm is reproduced in Figure 7b to facilitate comparison across the different units (blue diamonds in Figure 3a and 7b are the same isotherm). Critically, the observed CO_2_ absorption was even lower than what would be expected for the 90% [P_8881_][triaz] alone. In addition, the added ZIF‐8 did not lead to any significant change in the physisorption behavior. After the CO_2_ absorption experiment, the initially opaque colorless porous ionic liquid had turned transparent, suggesting dissolution of the ZIF‐8 solid phase, Figure 7c. This change in appearance was accompanied by a loss of CH_4_ physisorption capacity, Figure 7a. We did not observe reprecipitation of ZIF‐8 or other solids even after thorough degassing.

**Figure 8:**
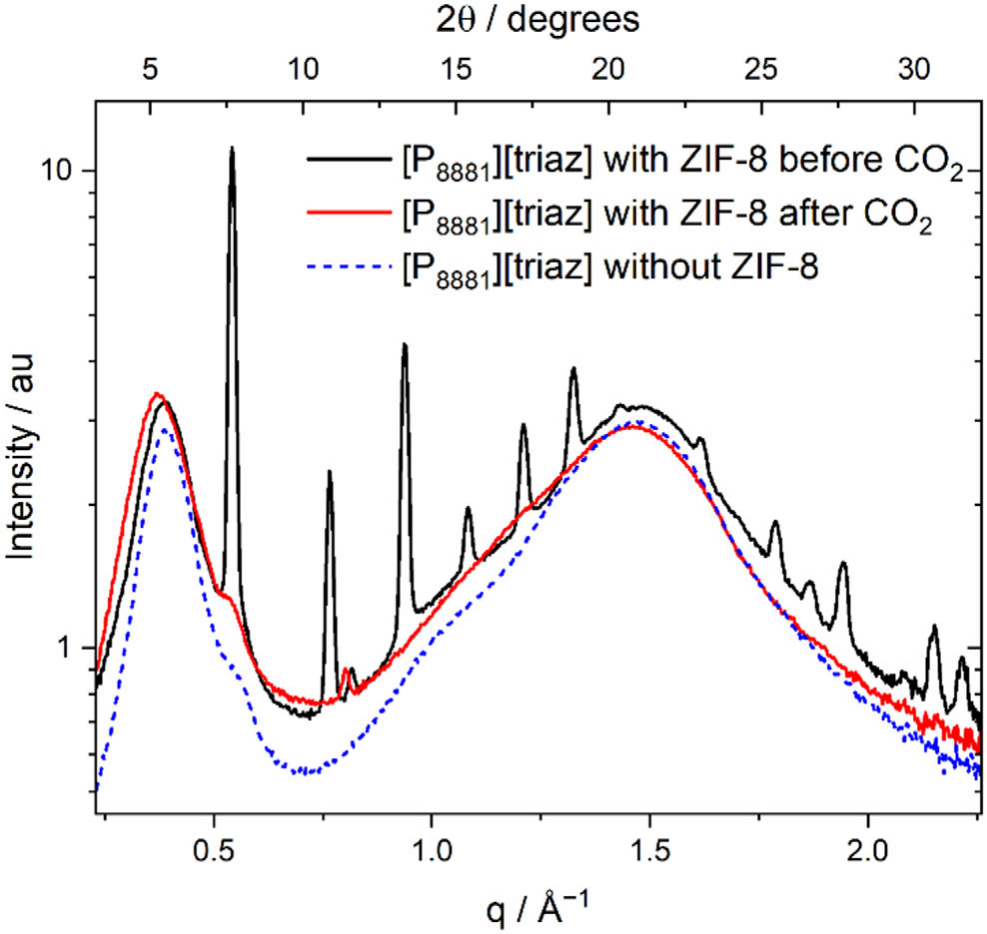
SAXS patterns showing the dissolution of ZIF‐8 in the ionic liquid after treatment with CO2. The small peak at *q* = 0.9 Å is known to result from hydrolysis products.^[^ ^35^ ^]^

The suspected dissolution of ZIF‐8 was confirmed through SAXS measurements depicted in **Figure** **8**. The SAXS pattern of the porous ionic liquid before CO_2_ treatment confirmed the simultaneous presence of both crystalline ZIF‐8 and the ionic liquid. This pattern was recorded several weeks after the preparation of the suspension, proving that dissolution does not occur in absence of CO_2_ on this time scale. In contrast, the peaks corresponding to the ZIF‐8 phase completely disappeared after stirring the sample under pure CO_2_ atmosphere (1 bar) at 70 °C for a few hours.

The degradation of the suspended ZIF‐8 in presence of CO_2_ can be rationalized considering the proton transfer energies represented in Figure 6, specifically the high basicity of isolated 2‐methylimidazolate, which is part of the ZIF‐8 structure together with zinc ions. The imidazolate by itself would be expected to react with CO_2_ analogous to triazolate or act as a proton scavenger. The imidazole formed in the reaction has a basicity similar to carbamate and carboxylate, which are dominant after saturation with CO_2_. Indeed, it is known that dissolution or formation of ZIF‐8 in aqueous media strongly depends on the conditions, first and foremost the pH.^[^ ^35^, ^36^ ^]^ Naturally, the dissolution of ZIF‐8 also requires the zinc ions to be solvated, which is not currently taken into account in our description. The presence of triazolate, which is chemically similar to imidazolate, might be a factor promoting the observed dissolution of ZIF‐8. This would explain why, in some cases, porosity is maintained even in the presence of CO_2_ chemisorption.^[^ ^23^ ^]^

## 4 Conclusions

In this work, we introduced a novel multifunctional ionic liquid, which was prepared without halide intermediates or the need for ion exchange. The ionic liquid exhibits competing pathways for reversible CO_2_ chemisorption either through the cation or through the anion, in addition to physisorption. We developed a holistic model to describe the total CO_2_ absorption and implemented it in a python script to fit experimental data. Our model successfully integrates chemical and physical interactions and explicitly captures their temperature and pressure dependance. The competition between cation and anion reactivity leads to unexpected trends at higher pressures, which went unnoticed to this point.

The addition of ZIF‐8 to this ionic liquid led to a porous ionic liquid, as confirmed by SAXS and CH_4_ uptake measurements. However, an important discovery emerged: the ZIF‐8 phase dissolved completely in the presence of CO_2_. This shows the necessity of considering the complex mixture resulting from the chemical reactions with CO_2_ when a porous solid is to be introduced, rather than just the neat and pure ionic liquid.

Notwithstanding the complexity of the resulting mixtures, we are able to fully rationalize all experimental findings using an approximate basicity scale from an in silico prediction. Specifically, the relative basicity of the species present in our system explains the accelerated diffusive dynamics after CO_2_ uptake, the competition between cation and anion reactivity, and the dissolution of ZIF‐8. This simple measure of acidity/basicity can be used to guide and inform the design of ionic liquids as CO_2_ absorbents.

Finally, our diffusion measurements suggest that the fluidity of our ionic liquid as a sorbent significantly increasing during CO_2_ uptake, which is a key advantage over most amine‐based sorbents. The fastest diffusion was observed for the acidic proton, which further underlines the importance of considering proton transfer processes in such a complex mixture.

## Conflict of Interest

The authors declare no conflict of interest.

## Supporting information

Supplementary Material

## Data Availability

The data that support the findings of this study are available in the Supporting Information of this article.
