# Supplementary material for: Can Gas Absorption be Tuned in a Multifunctional Ionic Liquid?
Source: ChemSusChem. 2025 Sep 2;18(20):e202501347. doi: 10.1002/cssc.202501347 (PMC12548951; doi:10.1002/cssc.202501347)
Supplement: Supplementary file 1 — Supplementary Material [file CSSC-18-e202501347-s001.zip › cssc202501347-sup-0001-SuppData-S1.pdf]

Supporting Information to

Can Gas Absorption be Tuned in a Multifunctional Ionic Liquid?

Frederik Philippi and Margarida Costa Gomes

Laboratoire de Chimie
ENS de Lyon and CNRS
Lyon, France

Contents

1. Numerical values used in graphs 3
2. Syntheses 6
   2.1 $[P_{8881}]$[triaz] 6
   2.2 $[P_{4441}]$[triaz] 6
   2.3 ZIF-8 and porous ionic liquid 6
3. Thermal transitions 7
4. Density and Viscosity 8
5. Gas absorption measurements 10
6. Modelling of CO_2 chemisorption 11
   6.1 Expression for mole ratio 13
   6.2 Expression for molality 14
   6.3 Expression for mole fractions 15
7. Modelling of physical absorption of CO_2 / CH_4 17
8. Heat Capacity 19
9. Diffusion coefficients 21
10. Kinetics of CO_2 absorption *via* NMR 26
11. X-ray Scattering 27
12. *Ab initio* calculations 30
13. References 31

1. Numerical values used in graphs

In Table S1 to Table S5, we provide a summary of the numerical values we used to prepare the figures in the main manuscript. The corresponding raw and processed data and the scripts used to process the data can be found in the accompanying files.

Table S1: Numerical values for data points in Figure 3a in the main manuscript.

Pressure / bar	$n(CO_2)$ / $n_0([P_{8881}][triaz])$		
	30 °C	50 °C	70 °C
0.00	0.00	0.03	0.00
0.25	0.43	0.33	0.22
0.50	0.58	0.44	0.30
0.75	0.65	0.51	0.35
1.00	0.70	0.57	0.40
2.50	0.85	0.74	0.57
5.00	0.98	0.88	0.71
7.50	1.08	0.98	0.79
10.0	1.16	1.06	0.87

Table S2: Numerical values for data points in Figure 3b in the main manuscript. The columns labelled with temperatures contain the data for the isotherm at this temperature as $1000 \cdot n(CH_4)$ / $n0([P_{8881}][triaz])$.

Pressure / bar	30 °C	Pressure / bar	50 °C	Pressure / bar	70 °C	70 °C	Pressure / bar	70 °C	70 °C
0.00	0.0	0.00	0.9	0.00	-1.2	-1.2	0.00	-1.2	-1.2
0.25	1.1	1.00	5.2	0.25	0.5	1.4	1.00	1.8	1.4
0.50	1.9	2.00	6.7	0.50	1.5	3.0	2.00	0.06	3.6
0.75	2.7	4.00	15.4	0.75	2.4	3.8	4.00	10.8	12.8
1.00	4.0	6.00	21.4	1.00	2.7	4.4	6.00	12.3	19.5
2.50	10.9	8.00	27.6	2.50	4.9	11.1	8.00	18.6	27.5
5.00	20.4	8.00	27.6	5.00	22.2	18.8	8.00	18.6	27.5
5.00	20.4	6.00	20.9	5.00	22.2	18.8	6.00	11.5	18.3
2.50	11.5	4.00	13.7	2.50	4.5	6.4	0.00	-1.2	
1.00	4.8	2.00	4.3	1.00	3.5	4.5			
0.75	3.3	1.00	1.3	0.75	3.6	4.6			
0.50	1.9	0.00	0.9	0.50	2.6	3.4			
0.25	0.2			0.25	1.2	1.8			
0.00	0.0			0.00	-1.2	-1.2			

Table S3: Numerical values for data points in Figure 6 in the main manuscript.

Base	Acid	Proton transfer energy / kJ mol^{-1}
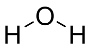	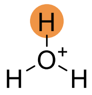	0
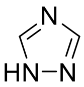	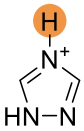	91
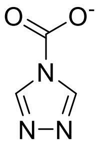	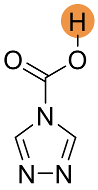	130
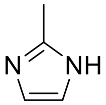	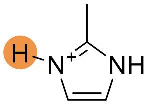	139
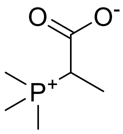	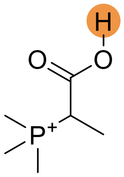	152
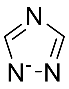	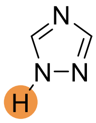	213
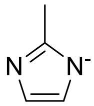	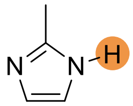	243
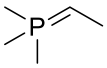	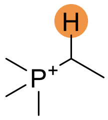	320
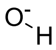	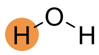	376

Table S4: Numerical values for data points in Figure 7a, calculated using the molality-based $K_H(\mathrm{CH_4})$.

System	CH_4 absorption capacity at 1 bar and 30°C
[P_{8881}][triaz], neat ionic liquid	9.2(2) µmol / g
9.7% ZIF-8 in [P_{8881}][triaz], before exposure to CO_2	19.5(5) µmol / g
10.0% ZIF-8 in [P_{8881}][triaz], before exposure to CO_2	11.6(8) µmol / g

Table S5: Numerical values for data points in Figure 7b.

Pressure / bar	$m(CO_2)$ / mmol g^{-1} neat $[P_{8881}]$[triaz]	Pressure / bar	$m(CO_2)$ / mmol g^{-1} neat ZIF-8	Pressure / bar	$m(CO_2)$ / mmol g^{-1} 9.7% w/w ZIF-8 in $[P_{8881}]$[triaz]
0.00	0.00	0.00	0.00	0.00	0.00
0.25	0.95	1.00	0.56	0.25	0.75
0.50	1.27	2.00	1.12	0.50	1.00
0.75	1.44	3.13	1.76	0.75	1.08
1.00	1.55	4.00	2.26	1.00	1.13
2.50	1.87	5.07	2.85	2.50	1.34
5.00	2.15	6.00	3.30	5.00	1.56
7.50	2.37	7.00	3.75	7.50	1.76
10.00	2.57	8.00	4.16	10.00	1.94
10.00	2.57	6.71	3.64	10.00	1.94
7.50	2.37	6.00	3.32	7.50	1.76
5.00	2.16	5.00	2.82	5.00	1.57
2.50	1.88	4.00	2.29	2.50	1.34
		3.00	1.73	1.00	1.13
		2.00	1.16	0.75	1.08
		1.00	0.60	0.50	1.01
		0.00	0.00		
		0.00	0.00		
		1.00	0.56		
		2.00	1.12		

2. Syntheses

An argon-filled glovebox with < 1 ppm oxygen and water (GP CAMPUS, JACOMEX, Lyon, France) was used to handle dried samples. The sample holder for gas absorption and the aluminium pans for DSC / heat capacity measurements were filled in this glovebox as well. All samples were dried on a vacuum line (≈ 10^{-3} mbar dynamic vaccum) for several days, which leads to a water content < 1000 ppm as determined *via* coulometric Karl-Fisher titration (C20S, Mettler Toledo, Greifensee, Switzerland).

2.1 [P_{8881}][triaz]

To 19.5 mL / 15.873 g / 11.2 mmol of methyltrioctylphosphonium methylcarbonate in methanol (assay 32.43% w/w by titration, IoLiTec Ionic Liquids Technologies, Heilbronn, Germany) were added 774.82 mg / 11.2 mmol 1,2,4-Triazole (99.5% w/w) as well as 1 drop of water. The resulting homogeneous reaction mixture was stirred at 40°C overnight while continuously injecting nitrogen gas into the mixture. The solvent was then removed under reduced pressure and the resulting ionic liquid dried under dynamic vacuum, yielding 4.13 g of the target compound (9.1 mmol / 81% yield).

^{1}H NMR (DMSO-d6, 400 MHz, δ in ppm): 7.60 (s, 2H, triazolate), 2.38-1.95 (m, 6H, P-C$\boldsymbol{H}_2$-), 1.79 (d, $^{2}J_{H/P}$ = 14.1 Hz, 3H, P-C$\boldsymbol{H}_3$), 1.58-1.05 (m, 36H, P-CH_2-(C$\boldsymbol{H}_2$)$_6$-CH_3), 0.87 (t, $^{3}J_{H/H}$ = 6.8 Hz , 9H, P-(CH_2)$_7$-C$\boldsymbol{H}_3$).

^{31}P{^{1}H} NMR (DMSO-d6, 162 MHz, δ in ppm): 32.29 (s).

^{13}C{^{1}H} NMR (DMSO-d6, 101 MHz, δ in ppm): 148.8 (s, triazolate), 31.2 (s), 30.0 (d, $^{3}J_{C/P}$ = 15.4 Hz), 28.4 (s), 28.2 (s), 22.0 (s), 20.5 (d, $^{2}J_{C/P}$ = 4.3 Hz), 19.0 (d, $^{1}J_{C/P}$ = 48.9 Hz), 13.9 (s), 3.0 (d, $^{1}J_{C/P}$ = 51.3 Hz).

2.2 [P_{4441}][triaz]

To 15.4828 g / 29.9 mmol of tributylmethylphosphonium methylcarbonate in methanol (assay 56.4% w/w by titration, IoLiTec Ionic Liquids Technologies, Heilbronn, Germany) were added 2.07265 g / 29.9 mmol 1,2,4-Triazole (99.5% w/w). The resulting homogeneous reaction mixture was stirred overnight. The solvent was then removed under reduced pressure and the resulting solid dried under dynamic vacuum, yielding 8.42 g of the target compound (29.5 mmol / 99% yield). The melting point of the resulting solid was determined to be 162.1 °C at a heating rate of 5 K min^{-1} (MP90 Melting Point System, Mettler Toledo).

^{1}H NMR (DMSO-d6, 400 MHz, δ in ppm): 7.59 (s, 2H, triazolate), 2.41-2.08 (m, 6H, P-C$\boldsymbol{H}_2$-), 1.81 (d, $^{2}J_{H/P}$ = 14.1 Hz, 3H, P-C$\boldsymbol{H}_3$), 1.65-1.28 (m, 12H, P-CH_2-(C$\boldsymbol{H}_2$)$_2$-CH_3), 0.90 (t, $^{3}J_{H/H}$ = 7.1 Hz , 9H, P-(CH_2)$_3$-C$\boldsymbol{H}_3$).

^{31}P{^{1}H} NMR (DMSO-d6, 162 MHz, δ in ppm): 32.46 (s).

^{13}C{^{1}H} NMR (DMSO-d6, 101 MHz, δ in ppm): 149.0 (s, triazolate), 23.3 (d, $^{3}J_{C/P}$ = 16 Hz), 22.6 (d, $^{2}J_{C/P}$ = 4.4 Hz), 18.8 (d, $^{1}J_{C/P}$ = 49.2 Hz), 13.2 (s), 2.9 (d, $^{1}J_{C/P}$ = 51.4 Hz).

2.3 ZIF-8 and porous ionic liquid

ZIF-8 (BASF Basolite Z1200 obtained through Sigma-Aldrich, Lot number STBG5528V) powder was passed through an 11 µm Nitex nylon mesh (1 h sieving, yield 480 mg) and dried for 5 h at 80°C under dynamic rotary vane pump vacuum. Two batches of porous ionic liquid were prepared using this material by adding [P_{8881}][triaz] to ZIF-8 in the glovebox and stirring overnight. Batch 1 was prepared from 60.8 mg ZIF-8 and 564 mg [P_{8881}][triaz] (9.7% w/w ZIF-8) and was used for CH_4 sorption isotherms before CO_2 treatment, the CO_2 sorption isotherm, and the SAXS measurements. Batch 2 was prepared from 231 mg ZIF-8 and 2.080 g [P_{8881}][triaz] (10.0% w/w ZIF-8) and was used for the visual representation in Figure 7c) in the manuscript and, after exposure to CO_2, to measure CH_4 absorption capacity for Figure7a (third bar from the left, red, diagonal pattern).

3. Thermal transitions

Thermal transitions were measured on a differential scanning calorimeter (DSC 8500 equipped with CLN2 liquid nitrogen cooling system, Perkin Elmer). The sample chamber was purged with 20 mL/min helium gas for the duration of the experiment. 6.825 mg of [P_{8881}][triaz] were placed and hermetically sealed in a aluminium pan inside a glovebox. The pan was placed in the instrument together with a reference pan. The sample was kept at 30°C for 1.5 h to allow for the heat flow to stabilise. After that, the temperature was decreased with 10 K/min to −100°C, held for 5 min, and increased with 10 K/min to 65°C, Figure S1. The experiment was repeated with slower temperature change rate of 2 K/min, Figure S2. Neither melting nor crystallisation was observed at any point.

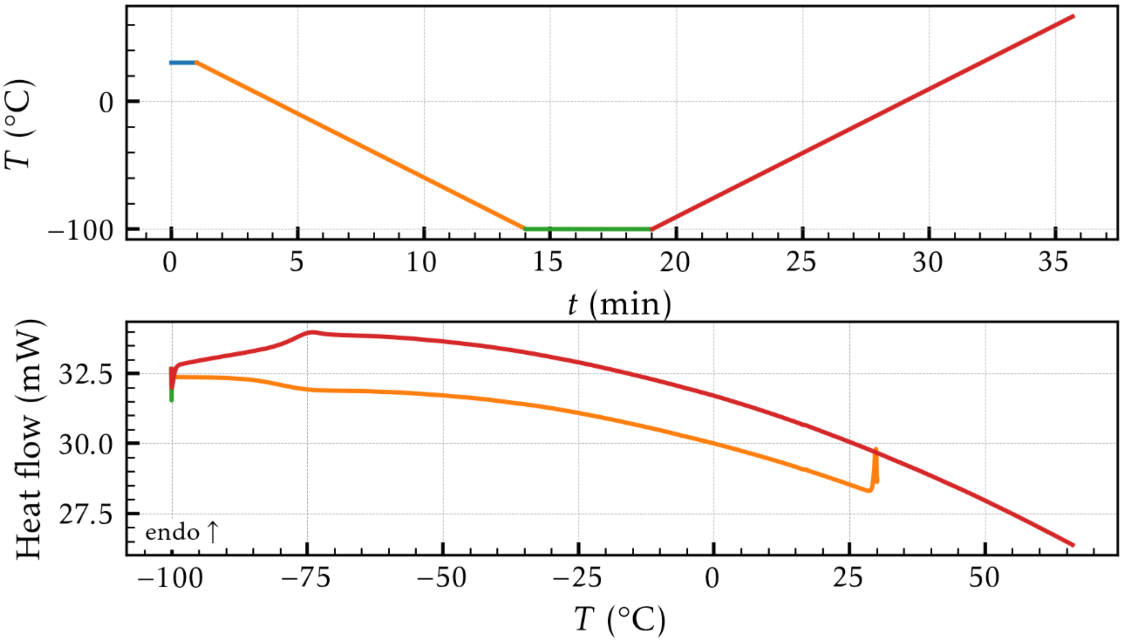

Figure S1: DSC trace of [P_{8881}][1,2,4-triazolate] at 10 K/min heating/cooling rate.

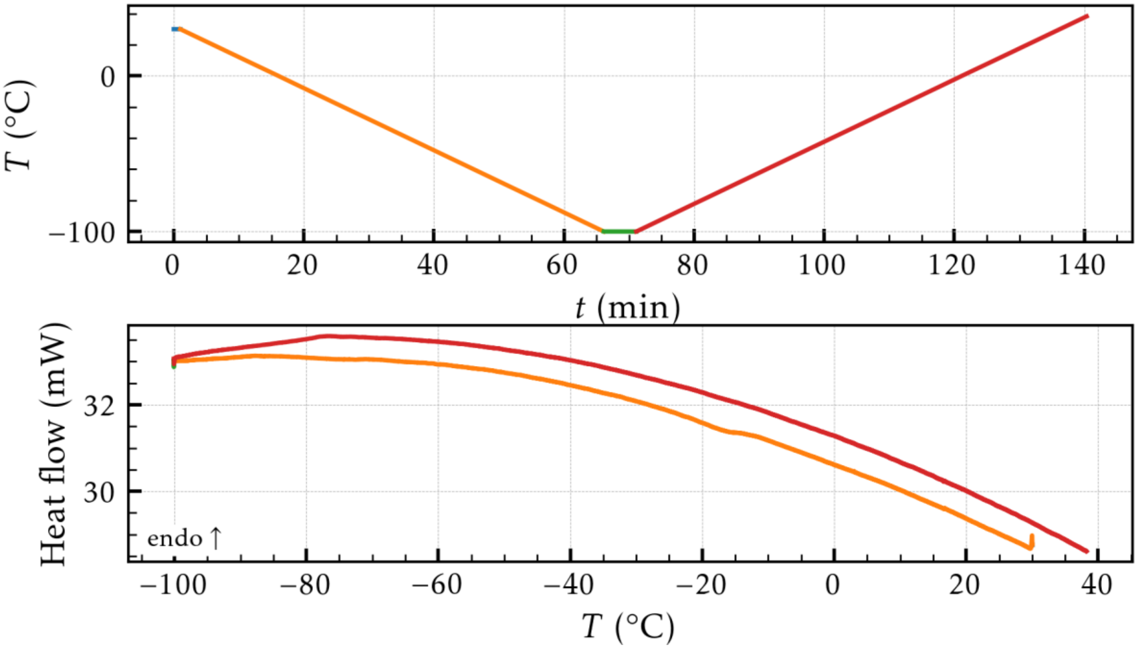

Figure S2: DSC trace of [P_{8881}][1,2,4-triazolate] at 2 K/min heating/cooling rate.

4. Density and Viscosity

Density and viscosity were measured from 20°C to 80°C on a combined viscometer / densitometer (SVM 3001, Anton Paar, les ulis, France). The viscometer / densitometer was not situated in a glovebox, and the sample was transferred while being exposed to air and moisture. After the measurement, a sample of [P_{8881}][1,2,4-triazolate] was recovered under air and the water content determined as 1427 ppm. Thus, we consider the impact of water uptake during these short exposures to atmospheric water as negligible. The results are given in Table S6. Based on the hysteresis (measurements at increasing / decreasing temperature), the estimated uncertainty is 5% of the absolute value for the viscosity and 0.02% of the absolute value for the density. The experimental density is shown in Figure S3 together with a linear fit. The experimental viscosity is shown in Figure S4 together with a Vogel-Fulcher-Tammann fit.[1–3]

Table S6: Viscosity and Density for [P_{8881}][1,2,4-triazolate] as a function of temperature, in order of the measurement.

Temperature / C°	Dynamic Viscosity / cP	Density / g cm^{-3}
20.0	2206	0.9174
25.0	1515	0.9144
30.0	1079	0.9115
40.0	557	0.9059
50.0	312	0.9003
60.0	184	0.8947
70.0	111	0.8890
80.0	73.3	0.8833
70.0	109	0.8889
60.0	176	0.8945
50.0	294	0.9001
40.0	522	0.9057

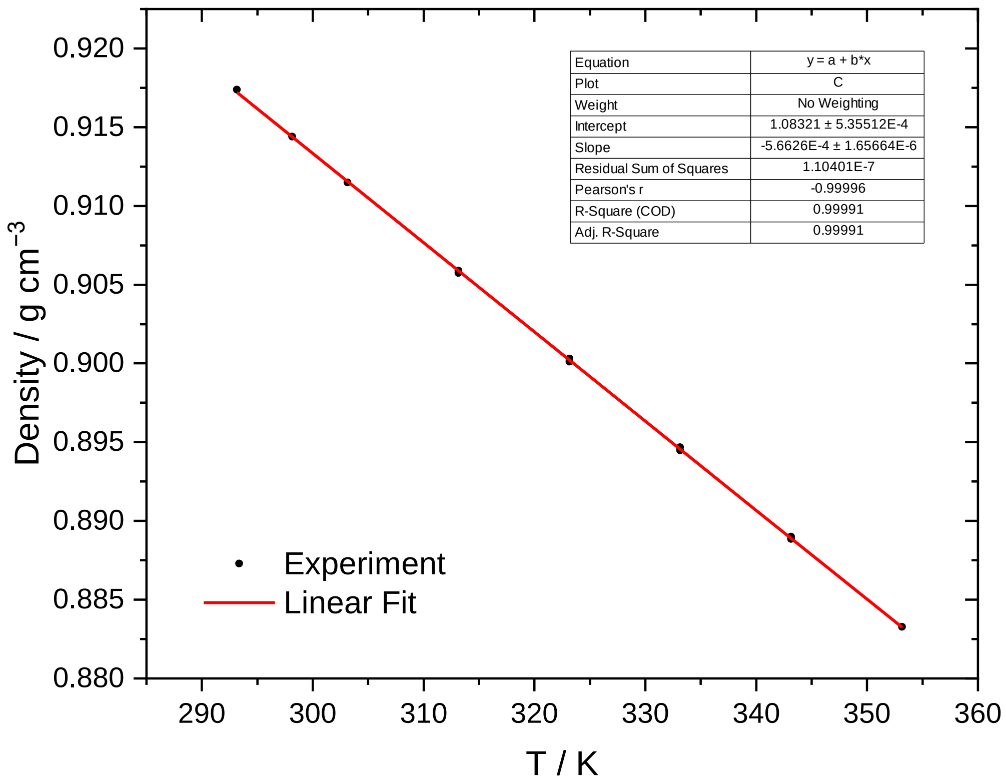

Figure S3: Density of [P_{8881}][1,2,4-triazolate] as a function of temperature and linear fit.

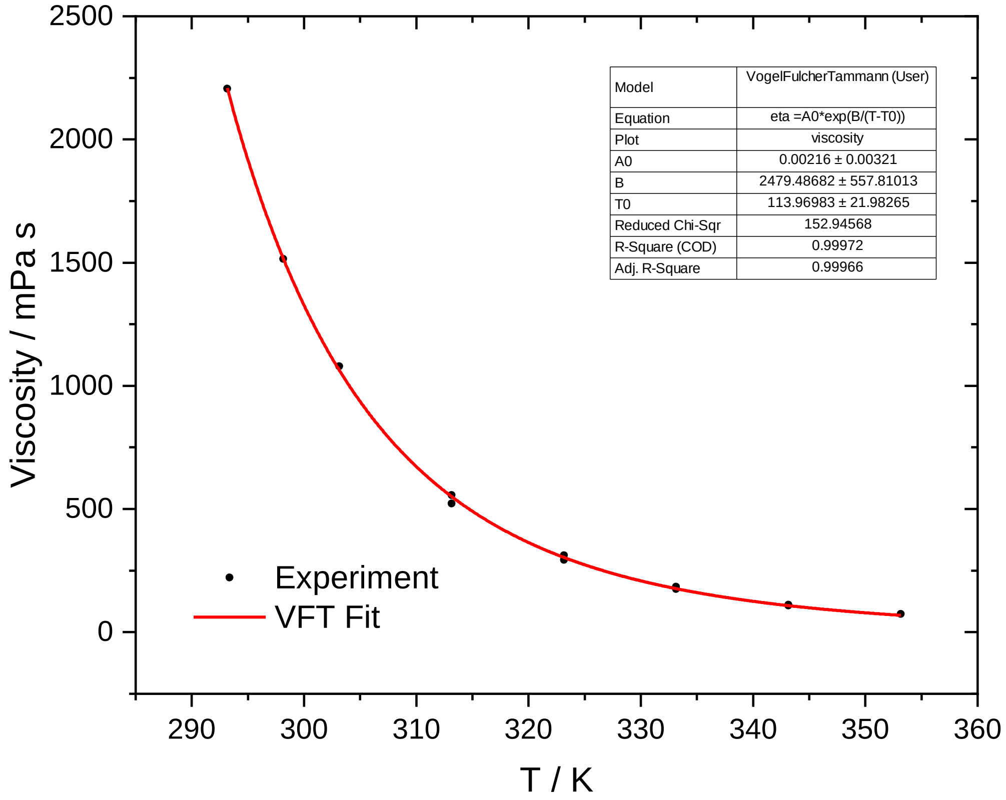

Figure S4: Viscosity of [P_{8881}][1,2,4-triazolate] as a function of temperature and VFT fit.

5. Gas absorption measurements

Gas absorption was measured using an IGA-001 Intelligent Gravimetric Analyzer (Hiden Isochema). The balance components are permanently kept at 55°C. The sample holder is suspended in a jacketed narrow vacuum vessel on a thin wire. The sample temperature was controlled with a GR150 recirculating water bath (Grant Instruments) supplying the jacket. Active cooling was turned off to improve temperature stability. The samples were placed in a platinum sample holder inside a glovebox which was then transferred into the pressure vessel of the gravimetric analyser. The samples were then degassed in turbopump vacuum for 24-72 h (20-100 mbar min^{-1} pressure ramp rate, final vacuum on the order of 10^{-6} mbar). The regime of free molecular flow is reached during degassing, temperature control of suspended balance components is no longer possible due to the insulating effects of the vacuum, and some samples show a small mass loss in vacuum. The otherwise common practise of vacuum bake out is not possible due to technical limitations of the setup. For these reasons, a small offset before and after degassing is regularly observed. The uncertainty of the weight measurements in vacuum are insignificant compared to the weight changes for samples with chemisorption, but are relevant compared to the weight changes due to physisorption. For these reasons, we have decided to exclude vacuum points from the analysis of isotherms without chemisorption.

The typical procedure to record an isotherm was as follows. After degassing, the pressure of the desired gas was increased (sorption scan) and subsequently decreased (desorption scan) stepwise to the desired state. Pressures were changed at a ramp rate of ≈50 mbar min^{-1} depending on the nature of the sample. The very fine ZIF-8 powder was measured with a slow pressure ramp rate 20 mbar min^{-1}.The gravimetric analyser uses a real time processor (RTP) with linear driving force model, which describes the uptake u as a function of time t with $u = \Delta u \cdot (1 - e^{-kt})$, where k is a constant and Δu the desired asymptotic value of the uptake. The time of the beginning of the pressure ramp is considered as $t = 0$. The analysed region ranges from $t/2$ to t. The real-time analysis is performed for a minimum weight change of 1 µg. Recording at each pressure point continues until u has reached 99.5% of the expected uptake Δu and the average deviation of the model from the data is less than 1 µg (7 µg for samples with dominant chemisorption), but at least for t_{min} and no longer than t_{max}. For samples with physisorption, $t_{min} = 2\ \mathrm{h}$ and $t_{max} = 3\ \mathrm{h}$ was used. For samples with chemisorption, $t_{min} = 3\ \mathrm{h}$ and $t_{max} = 15\ \mathrm{h}$ was used.

The total mass $m(\mathrm{CO_2})$ of absorbed CO_2 in the sample, and thus $n(\mathrm{CO_2})$, can be obtained from the raw weight m_{raw} measured by the gravimetric analyser using Equation (S1). To convert the experimental changes in weight during the gravimetric experiment into changes in mass, we correct for the buoyancy of the sample by considering the temperature dependent density of the ionic liquid and for the buoyancy of the balance components by performing an empty measurement.[4]

$$m(\mathrm{CO_2}) = m_{\mathrm{raw}} + m_0 \cdot \frac{\rho_{\mathrm{CO_2}}}{\rho_{\mathrm{sample}}} - A_{CO_2} \cdot P - m_0 \qquad \text{(S1)}$$

Here, m_0 is the initial mass of the sample, $\rho_{\mathrm{CO_2}}$ is the density of the gas at the given temperature and pressure obtained from the virial expansion using a temperature dependent polynomial for the second virial coefficient.[5] Thus, $m_0\, \rho_{\mathrm{CO_2}}/\rho_{\mathrm{sample}}$ is the buoyancy correction for the gas displaced by the sample, and ρ_{sample} is taken from the linear fit in Figure S3. The correction for all other balance components including the sample holder is contained in the correction factor A_{CO_2}, which is obtained from the slope of dm_{raw}/dP of an empty measurement with the sample holder but without sample.

The procedure to obtain $m(\mathrm{CH_4})$ from m_{raw} is entirely analogous, with empty measurements of the sample holder in $\mathrm{CH_4}$ to obtain A_{CH_4} and using $\rho_{\mathrm{CH_4}}$ instead of $\rho_{\mathrm{CO_2}}$.

6. Modelling of CO_2 chemisorption

This section describes how CO_2 absorption in [P_{8881}][triaz] was modelled. All models use as first step Henry's law to describe physical dissolution of CO_2 in the ionic liquid, Figure S5 and Equation (S2).

$$\mathrm{CO_2\ (sol)} \underset{}{\overset{K_H}{\rightleftharpoons}} \mathrm{CO_2\ (gas)}$$

Figure S5: equilibrium between physically dissolved and gas phase CO_2.

Here, we define K_H as the Henry's law volatility constant.[6] In general terms, it is the proportionality constant between the (partial) pressure of CO_2 in the gas phase above the liquid and the concentration c of physically dissolved CO_2 in the liquid phase.

$$K_H = \frac{P}{c\left(\mathrm{CO_2\ (sol)}\right)} \qquad \text{(S2)}$$

The anion reacts according to Figure S6.

$$\mathrm{CO_2\ (sol)} + [\mathrm{P_{8881}}][\mathrm{triaz}] \overset{K_A}{\rightleftharpoons} [\mathrm{P_{8881}}][\mathrm{triaz\text{-}CO_2}]$$

Figure S6: Reaction of the anion with CO_2 considered for the model.

With the equilibrium constant defined in Equation (S3).

$$K_A = \frac{c([\mathrm{P_{8881}}][\mathrm{triaz\text{-}CO_2}])}{c\left(\mathrm{CO_2\ (sol)}\right) \cdot c([\mathrm{P_{8881}}][\mathrm{triaz}])} = \frac{\xi_A}{\frac{P}{K_H}(n_0 - \xi_A \boldsymbol{- 2\xi_C})} \qquad \text{(S3)}$$

We use a 1:2 stoichiometry to describe the reactivity of the cation, Figure S7.

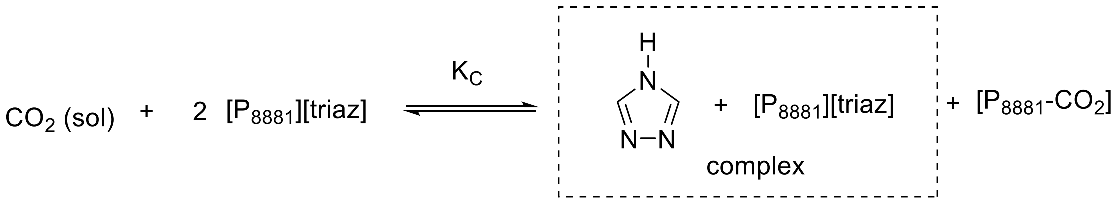

Figure S7: Reaction of the cation with CO_2 considered for the model. The complex between the hydrogen bond donor formed in the reaction (here triazole) and an ionic liquid ion pair as proposed in the literature is treated as single component.[7,8] This gives the equilibrium constant according to (S4).

$$K_C = \frac{c([\mathrm{P_{8881}\text{-}CO_2}]) \cdot c(\mathrm{complex})}{c\left(\mathrm{CO_2\ (sol)}\right) \cdot c([\mathrm{P_{8881}}][\mathrm{triaz}])^2} = \frac{(\xi_C)^2}{\frac{P}{K_H}(n_0 - 2\xi_C \boldsymbol{- \xi_A})^2} \qquad \text{(S4)}$$

The two reactions compete for the triazolate, which leads to the red terms in Equation (S3) and Equation (S4). The reactions of CO_2 with cation and anion have the same number of components on each side, hence the denominator of the concentration cancels out in this case, and the choice of concentration enters indirectly through P/K_H. However, the explicit form of the fitted expression for the total CO_2 concentration and thus the units of the three equilibrium constants depend on how concentrations are defined. For the sake of clarity, the definitions used in this work are given in separate subsections below.

The model parameters need to be adjusted so that the sum of physisorbed CO_2 (estimated *via* Henry's Law) and chemisorbed CO_2 (which is $\xi_A + \xi_C$) from the model follows $n(\mathrm{CO_2})$ from the experiment. The temperature dependence is introduced into Equation (S2)-(S4) using the integrated Van't Hoff equation (S5).

$$K = K_{T_{ref}} \exp\left(\frac{\Delta H}{R}\left(\frac{1}{T_{ref}} - \frac{1}{T}\right)\right) \tag{S5}$$

Finally, the experimental data, *i.e.* $m(\mathrm{CO_2})$ or rather $c(\mathrm{CO_2})$ as a function of temperature and pressure, is fitted using the provided python script. Points at the same pressure and temperature (from multiple repeats) were averaged before the fit to ensure equal weighting.

6.1 Expression for mole ratio

The total CO_2 concentration can be defined in terms of a mole ratio relative to the initial amount of ionic liquid, n_0, Equation (S6).

$$c(\mathrm{CO_2}) \stackrel{\mathrm{def}}{=} \frac{n(\mathrm{CO_2})}{n_0} = \frac{n(\mathrm{CO_2}\ (\mathrm{sol})) + \xi_A + \xi_C}{n_0} = \frac{P}{K_H} + \frac{\xi_A}{n_0} + \frac{\xi_C}{n_0} \tag{S6}$$

Here, Equation (S2) becomes

$$K_H = \frac{P}{n(\mathrm{CO_2}\ (\mathrm{sol}))} \cdot n_0 \tag{S7}$$

Thus, K_H has units of pressure, K_A and K_C are unitless. The fit results are summarised in Table S7. The fit is plotted together with the data in Figure S8.

Table S7: Fit results using Equation (S6) with $T_{ref} = 303.15$ K.

	Henry	Cation	Anion
K	37(3) bar	27(11)	112(11)
ΔH	13(4) kJ mol^{-1}	−22(11) kJ mol^{-1}	−26(6) kJ mol^{-1}

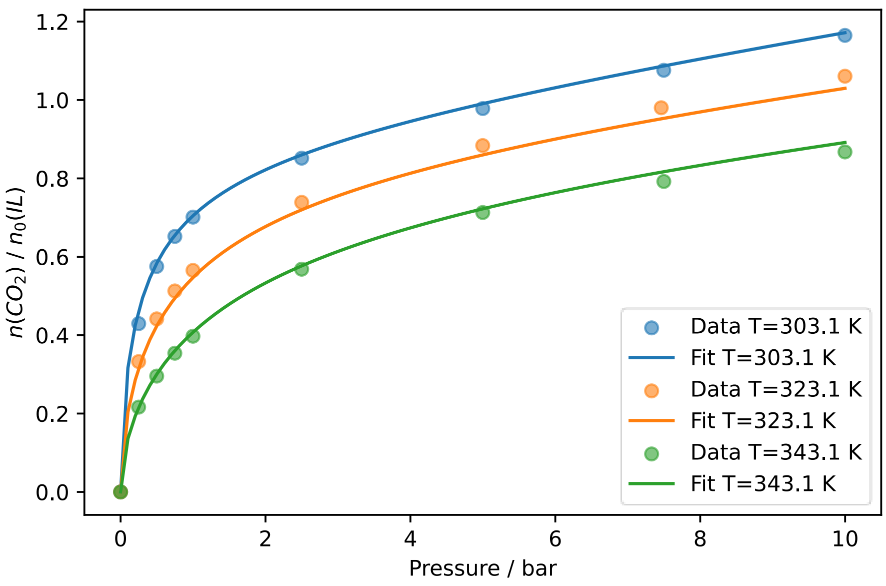

Figure S8: Data and fit using mole ratio.

6.2 Expression for molality

The total CO_2 concentration can be defined as molality, *i.e.* relative to the initial mass of ionic liquid, m_0, Equation (S8).

$$c(\mathrm{CO_2}) \stackrel{\mathrm{def}}{=} \frac{n(\mathrm{CO_2})}{m_0} = \frac{n(\mathrm{CO_2}\ (\mathrm{sol})) + \xi_A + \xi_C}{m_0} = \frac{P}{K_H} + \frac{\xi_A}{m_0} + \frac{\xi_C}{m_0} \quad \text{(S8)}$$

Here, Equation (S2) becomes

$$K_H = \frac{P}{n(\mathrm{CO_2}\ (\mathrm{sol}))} \cdot m_0 \quad \text{(S9)}$$

Thus, K_H has units of pressure times mass divided by amount of substance. K_A and K_C have units of mass divided by amount of substance. The fit results are summarised in Table S8. The fit is plotted together with the data in Figure S9.

Table S8: Fit results using Equation (S8) with $T_{ref} = 303.15$ K.

	Henry	Cation	Anion
K	17(1) bar kg mol^{-1}	12(5) kg mol^{-1}	51(5) kg mol^{-1}
ΔH	13(4) kJ mol^{-1}	−22(11) kJ mol^{-1}	−26(6) kJ mol^{-1}

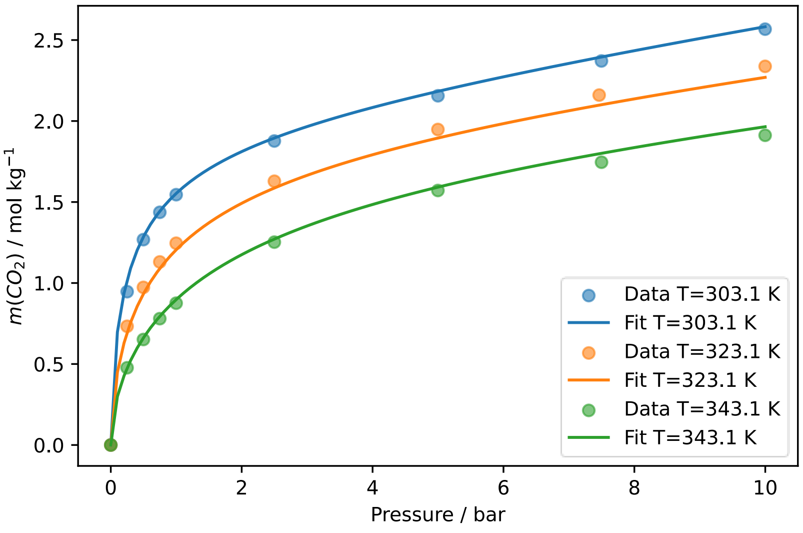

Figure S9: Data and fit using molality.

6.3 Expression for mole fractions

The total CO_2 concentration can be defined as mole fraction x, *i.e.* relative to the total amount of substance, Equation (S10). It is worth noting that we use a simplified mole fraction which is conceptually close to the definition of the mole ratio.

$$c(\mathrm{CO_2}) \stackrel{\mathrm{def}}{=} \frac{n(\mathrm{CO_2})}{n(\mathrm{CO_2}) + n_0} = \frac{1}{1 + \frac{n_0}{n(\mathrm{CO_2})}} \quad \text{(S10)}$$

Here, Equation (S2) becomes

$$K_H = \frac{P}{x\big(\mathrm{CO_2\ (sol)}\big)} = \frac{P}{n\big(\mathrm{CO_2\ (sol)}\big)} \cdot \left(n(\mathrm{CO_2}) + n_0\right) \quad \text{(S11)}$$

The total amount of absorbed CO_2 is thus given by Equation (S12).

$$n(\mathrm{CO_2}) = n\big(\mathrm{CO_2\ (sol)}\big) + \xi_A + \xi_C = \frac{P}{K_H}\left(n(\mathrm{CO_2}) + n_0\right) + \xi_A + \xi_C \quad \text{(S12)}$$

It is then convenient to solve for $n(\mathrm{CO_2})$, which yields Equation (S13).

$$n(\mathrm{CO_2}) = \frac{\frac{P}{K_H} n_0 + \xi_A + \xi_C}{1 - \frac{P}{K_H}} \quad \text{(S13)}$$

Finally, substituting Equation (S13) into Equation (S10) gives Equation (S14).

$$x(\mathrm{CO_2}) = \frac{\frac{P}{K_H} n_0 + \xi_A + \xi_C}{\xi_A + \xi_C + n_0} \quad \text{(S14)}$$

Thus, K_H has units of pressure, K_A and K_C are unitless. The fit results are summarised in Table S9. The fit is plotted together with the data in Figure S10.

Table S9: Fit results using Equation (S14) with $T_{ref} = 303.15\ \mathrm{K}$.

	Henry	Cation	Anion
K	77(7) bar	56(20)	239(26)
ΔH / kJ mol^{-1}	13(5)	−24(9)	−25(6)
ΔS / J mol^{-1} K^{-1}	78(16)	−45(31)	−37(20)

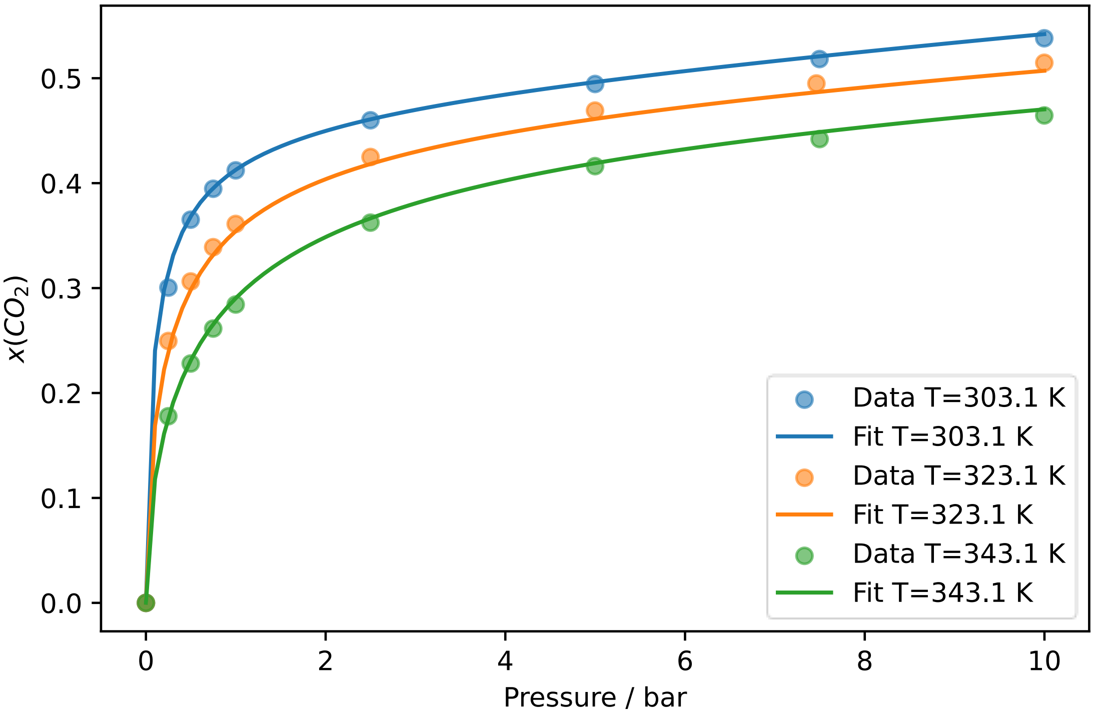

Figure S10: Data and fit using mole fraction.

7. Modelling of physical absorption of CO_2 / CH_4

Physical absorption of CO_2 and CH_4 in inert media, *i.e.* ionic liquids and ZIF-8 which are not reacting / chemisorbing, was described with Henry's Law, Equation (S9). The molality was used to be able to compare the neat ionic liquid with the porous liquid and the porous solid. The fits can be found in the accompanying files. A summary of the Henry volatility constants for methane is given in Table S10. In addition, physisorption of CO_2 in ZIF-8 was measured, and a $K_H(\mathrm{CO_2}) = 1.89(3)$ bar kg mol^{-1} was obtained.

Table S10: $K_H(\mathrm{CH_4})$ obtained from absorption isotherms.

		molality	Mole fraction	Mole ratio
Sample	Temperature	$K_H(\mathrm{CH_4})$ / bar kg mol^{-1}	$K_H(\mathrm{CH_4})$ / bar	$K_H(\mathrm{CH_4})$ / bar
[P_{8881}][triaz]	30°C	109(3)	246(6)	241(6)
	50°C	127(6)	287(15)	279(14)
	70°C	152(9)	342(20)	333(20)
[P_{8881}][triaz] + 10% ZIF-8	30°C	51(1)		
ZIF-8	30°C	5.03(4)		
	50°C	6.82(5)		
	70°C	8.7(2)		

The dissolution enthalpy was $\Delta H = -12.2(3)$ kJ mol^{-1} for CH_4 in ZIF-8, Figure S11, and $\Delta H = -6.9(4)$ kJ mol^{-1} for CH_4 in neat [P_{8881}][triaz], Figure S12. This value (expectedly) matches the dissolution enthalpy for CH_4 in neat [P_{8881}][triaz] calculated based on mole fractions within experimental error, Figure S13, $\Delta H = -7.1(2)$ kJ mol^{-1}.

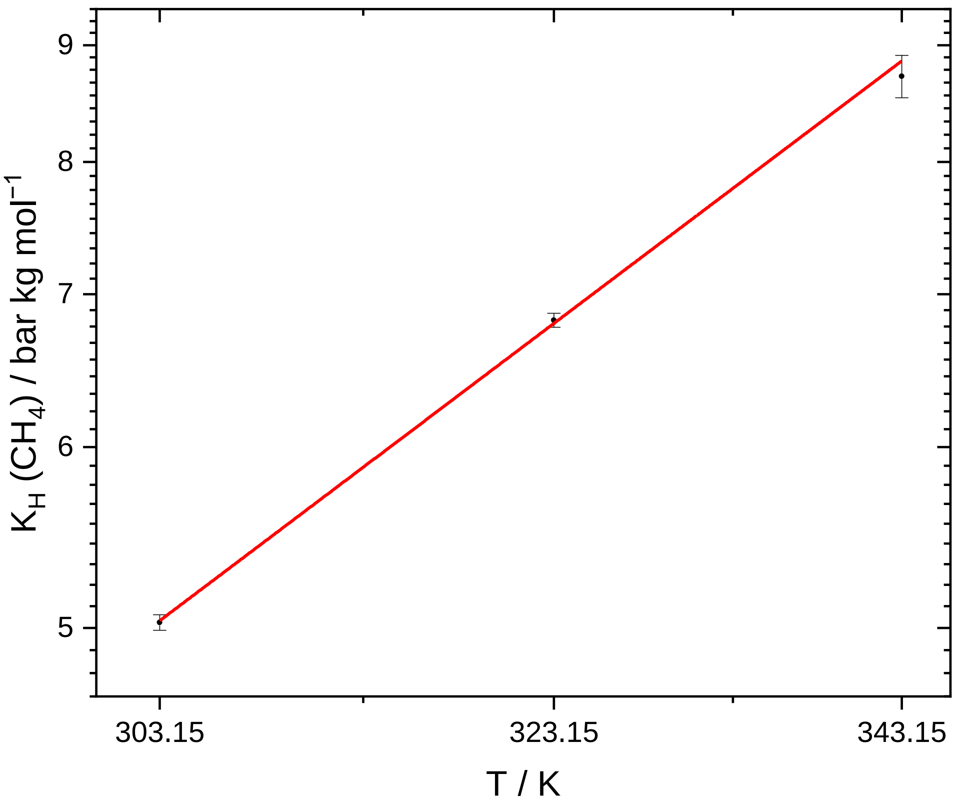

Figure S11: van't Hoff plot of the molality based Henry's law volatility constant $K_H(\mathrm{CH_4})$ for ZIF-8.

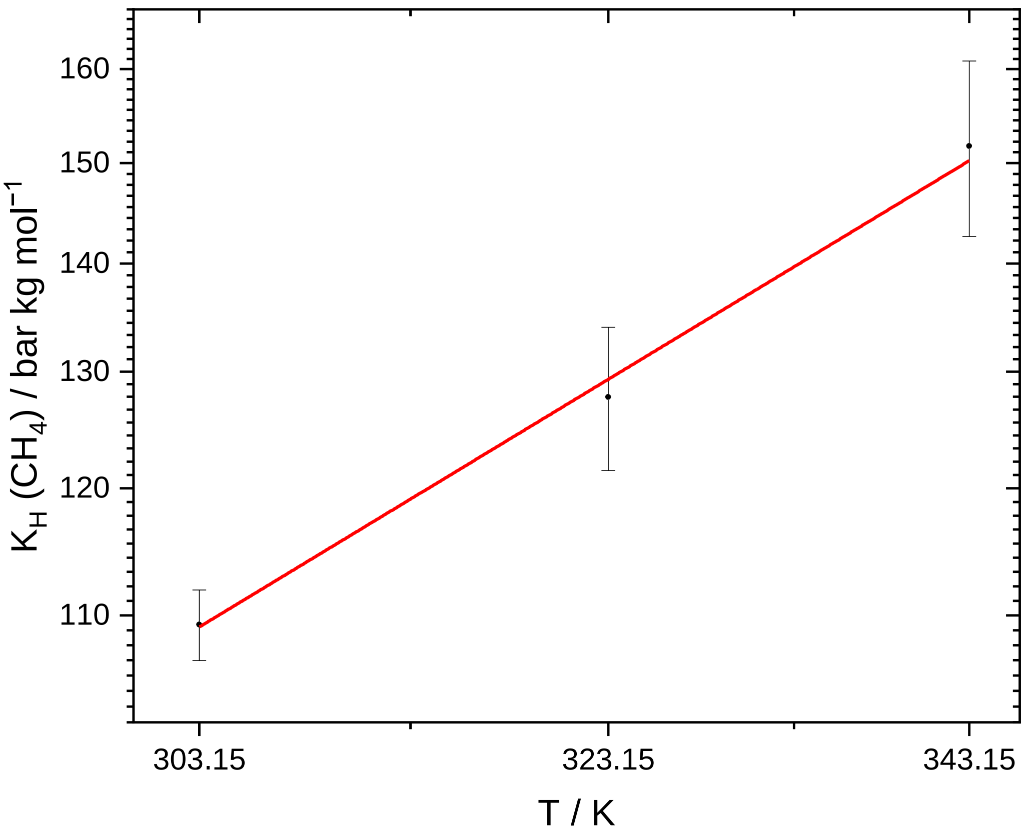

Figure S12: van't Hoff plot of the molality based Henry's law volatility constant $K_H(\mathrm{CH_4})$ for [P_{8881}][triaz].

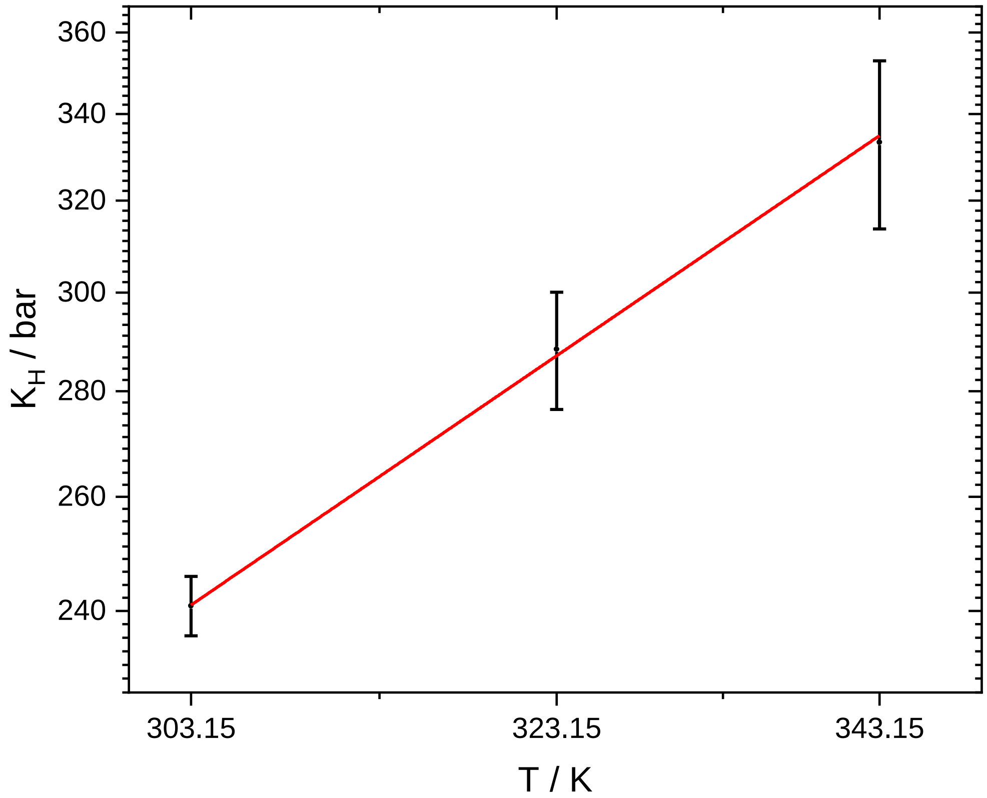

Figure S13: van't Hoff plot of the mole fraction based Henry's law volatility constant $K_H(\mathrm{CH_4})$ for [P_{8881}][triaz].

8. Heat Capacity

Heat capacities were measured on a thermal analyser (SENSYS evo with calvet 3D sensor, SETARAM / KEP technologies, France) under Argon flow (50 mL/min). Before the measurement, the sample was kept at 22.5 °C for 30 min. The heat capacity as a function of temperature was then measured in 16 steps of 5 °C with a ramp rate of 10 K/min up to 102.5 °C and calculated as the average over each 5 °C range. The steps were separated by an isothermal hold for 20 min. The samples were filled and sealed into 270 µL aluminium crucibles (6.7 mm x 11.3 mm, SETARAM / KEP technologies, France) inside a glovebox. Two measurements with empty pans were performed to be able to calculate the absolute heat capacity. Sapphire and [C_2C_1Im][NTf_2] were measured for verification and comparison to literature data, *cf*. Figure S14 and Figure S15.[9,10] Two independently synthesised samples of [P_{8881}][1,2,4-triazolate] were measured, the resulting heat capacity as a function of temperature is shown in Figure S16 together with a linear fit.

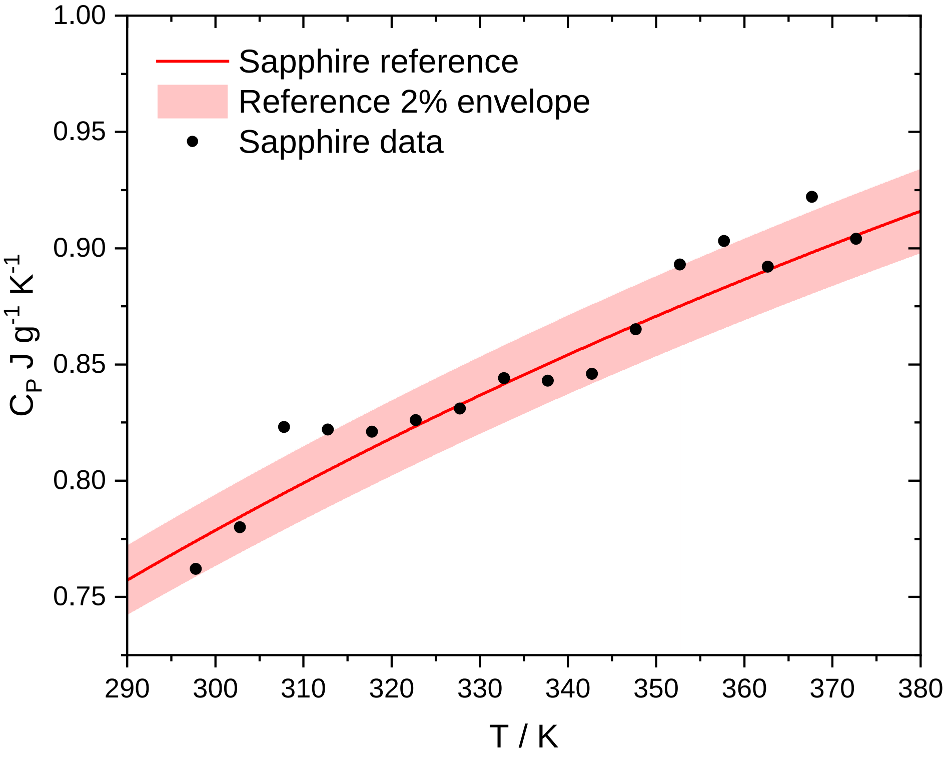

Figure S14: Heat capacity of sapphire reference material as a function of temperature. Reference curve from the literature.[10] The average absolute deviation from the reference curve was 1.2%.

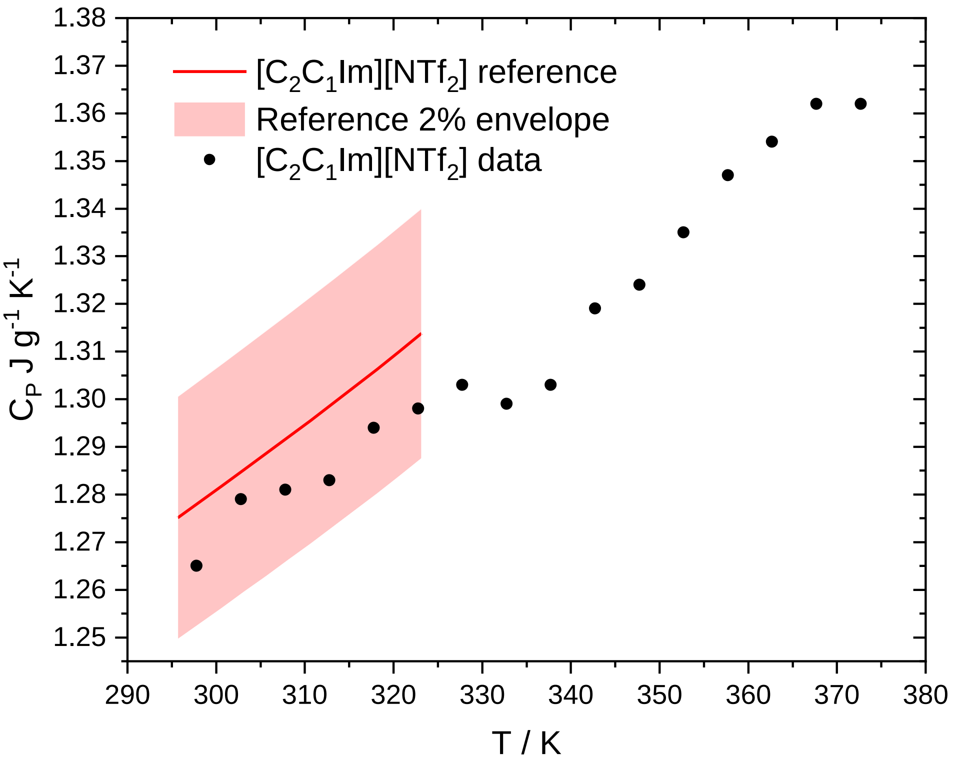

Figure S15: Heat capacity of as a function of temperature. Reference curve from the literature.[9] The average absolute deviation from the reference curve was 1.9%.

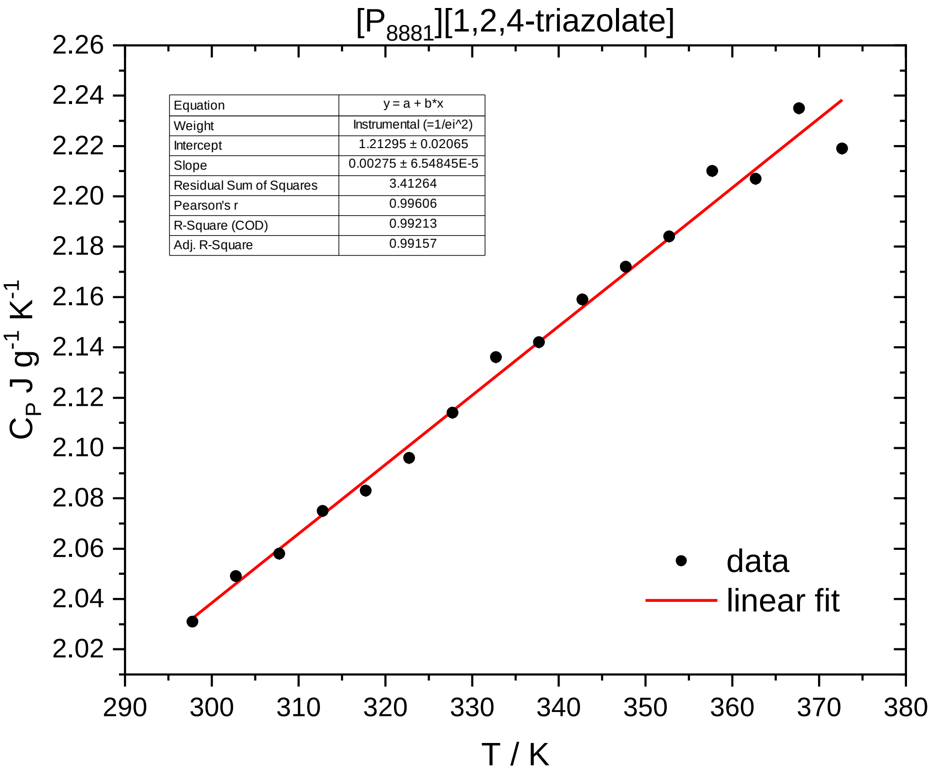

Figure S16: Heat capacity of $[P_{8881}]$[1,2,4-triazolate] as a function of temperature.

9. Diffusion coefficients

NMR diffusometry was performed on a Bruker 400 Avance III spectrometer equipped with BBIDiff probe. A pulsed field gradient stimulated echo (PFGSTE) pulse sequence with longitudinal eddy current delay and bipolar magnetic field gradients was used (ledbpgp2s in the Bruker library).[11] A smoothed square shape was used for the magnetic field gradients (SMSQ10.100), with a maximum effective magnetic field gradient of ≈2.6 T/m. Spoiler gradients of 0.8 ms duration (p19) were used, with a strength of −17.13% and −13.17% after the first/encoding bipolar pulse pair and the second/decoding bipolar pulse pair, respectively, and a gradient recovery delay τ (d16) of 1.2 ms. The LED delay was set to 5 ms (d21). 4 dummy scans were used for each experiment. For $^{31}P\{^1H\}$ experiments, 32 scans were used for each gradient strength. For ^1H experiments, 16 scans were used for each gradient strength. 40 gradients of increasing strength were used to generate the required arrayed data, keeping diffusion time Δ (d20) and pulse duration δ (2×p30) constant. The relaxation delay was 1 s for ^1H and 5 s for $^{31}P\{^1H\}$ experiments. The diffusion coefficients D were obtained using the Stejskal-Tanner equation (S15), more specifically from the slope of a linear fit as given by equation (S16).

$$\ln\left(\frac{I}{I_0}\right) = -D\gamma^2 g^2 \delta^2 \left(\Delta - \frac{\delta}{3} - \frac{\tau}{2}\right) = -DQ \qquad \text{(S15)}$$

$$D = \frac{d(-\ln(I))}{d(Q)} \qquad \text{(S16)}$$

Here, γ is the gyromagnetic ratio, g the gradient strength, and I the intensity of the NMR signal.

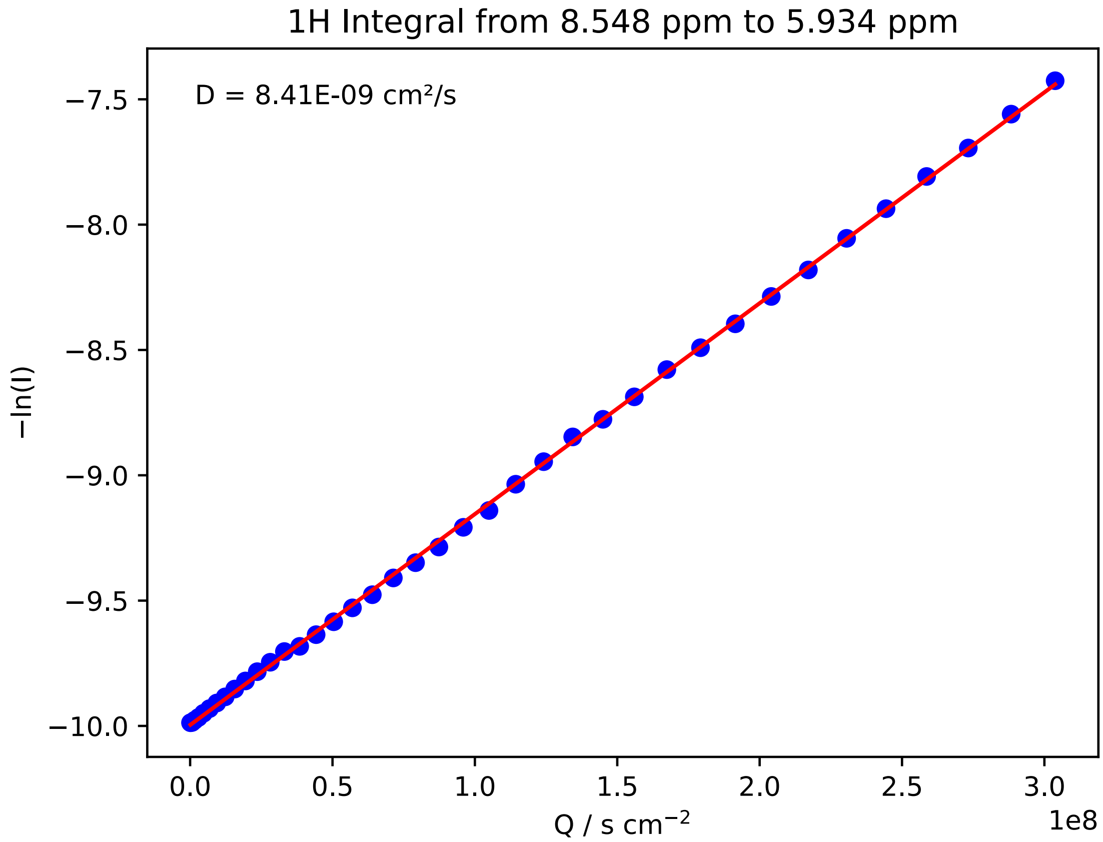

Figure S17: $[P_{8881}]$[1,2,4-triazolate], ^1H PFGSTE experiment, anion signal.

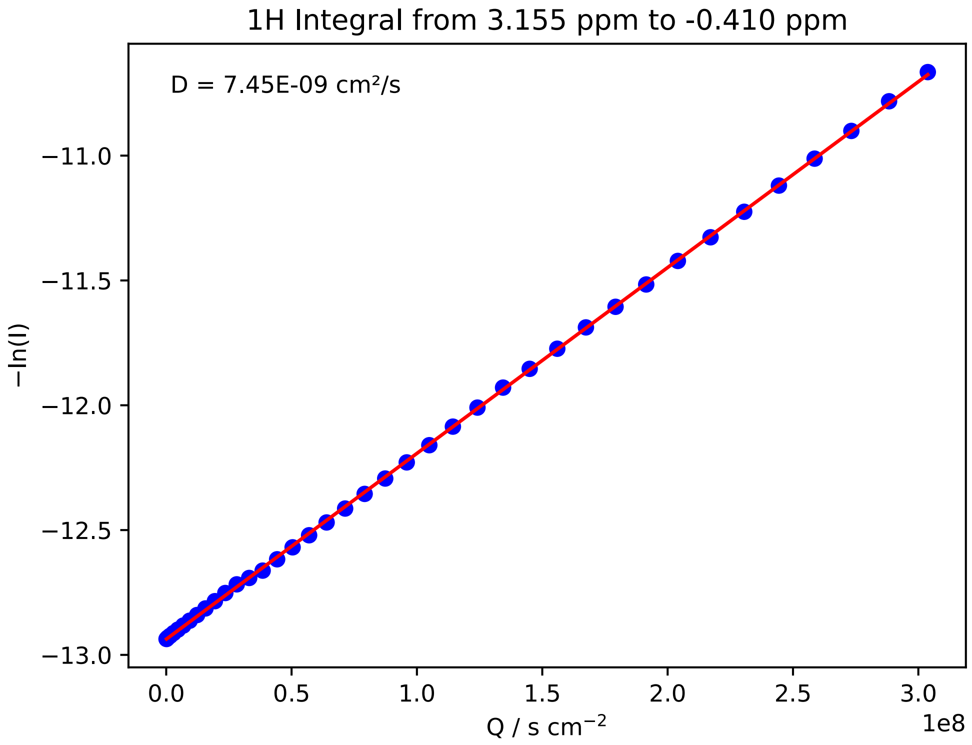

Figure S18: [P_{8881}][1,2,4-triazolate], ^{1}H PFGSTE experiment, remaining signals

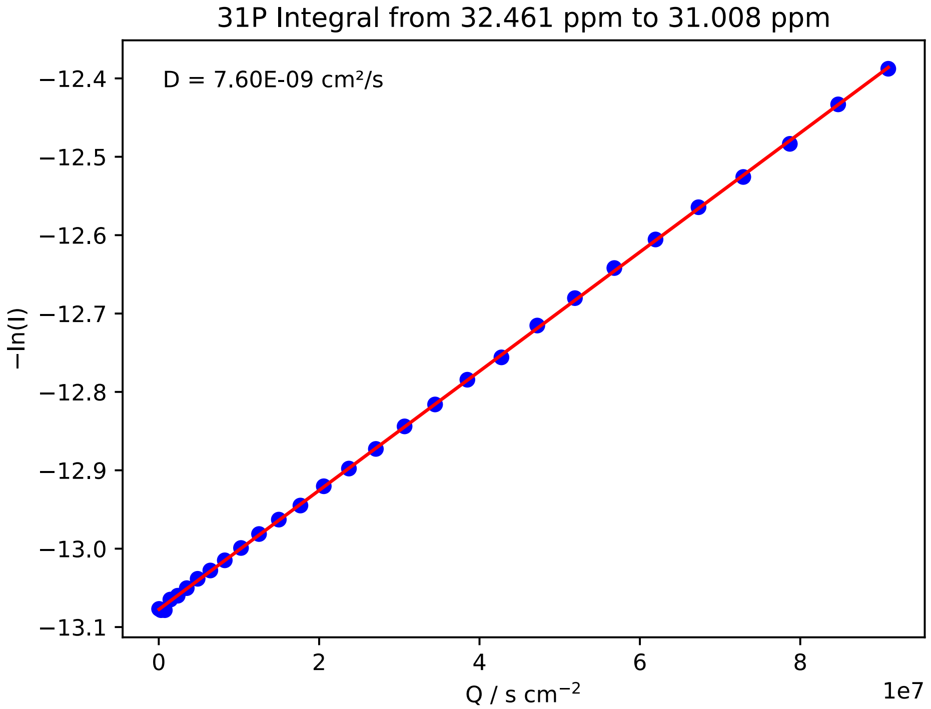

Figure S19: [P_{8881}][1,2,4-triazolate], ^{31}P PFGSTE experiment, cation signal.

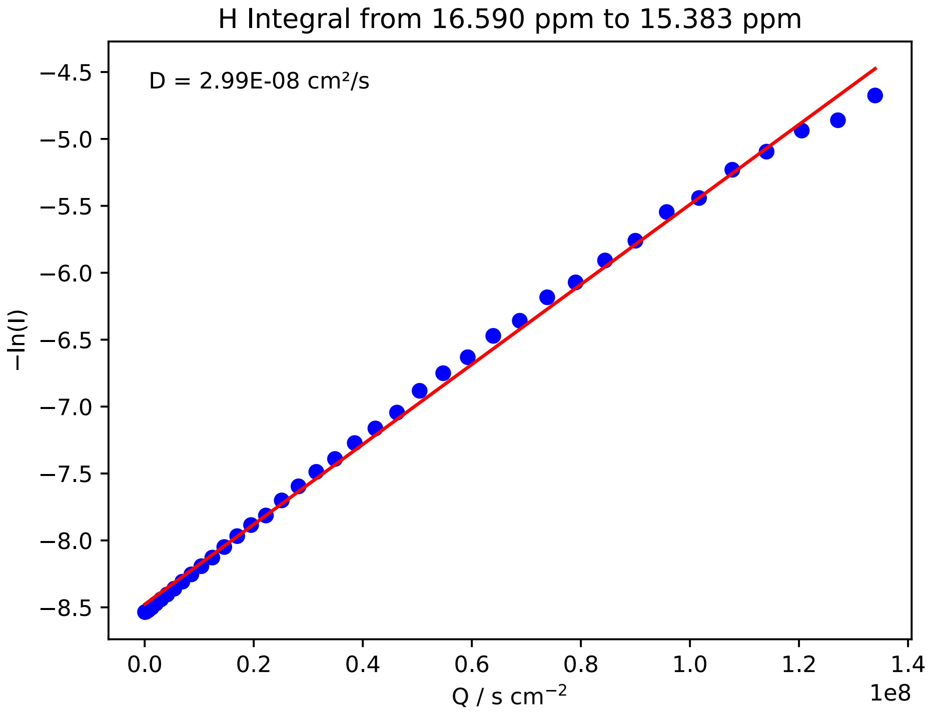

Figure S20: $[P_{8881}]$[1,2,4-triazolate] after saturation with CO_2, ^1H PFGSTE experiment, acidic proton.

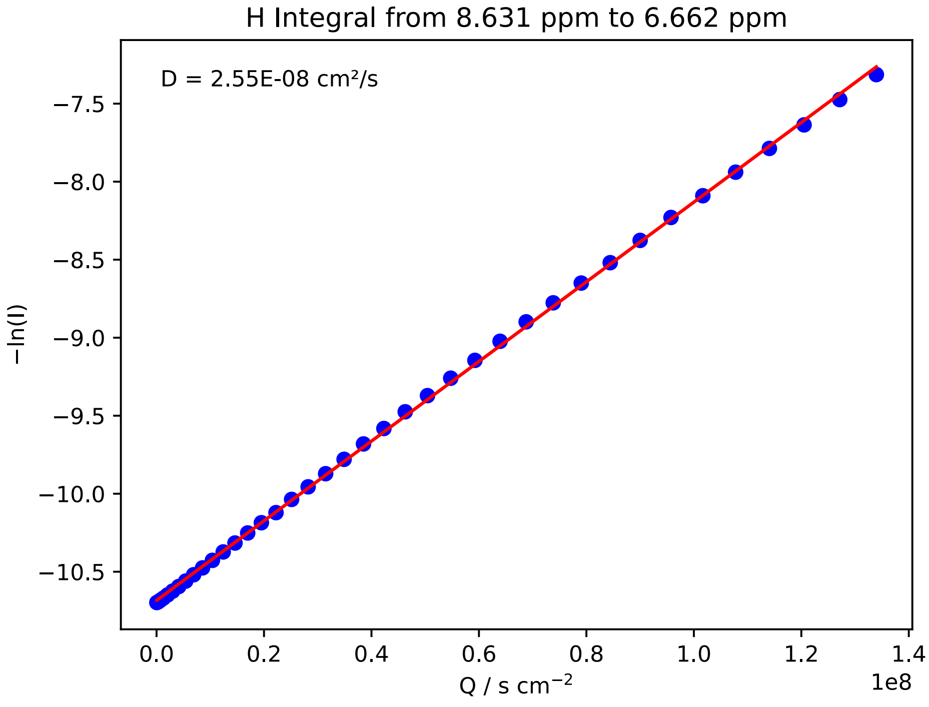

Figure S21: $[P_{8881}]$[1,2,4-triazolate] after saturation with CO_2, ^1H PFGSTE experiment, anion signal.

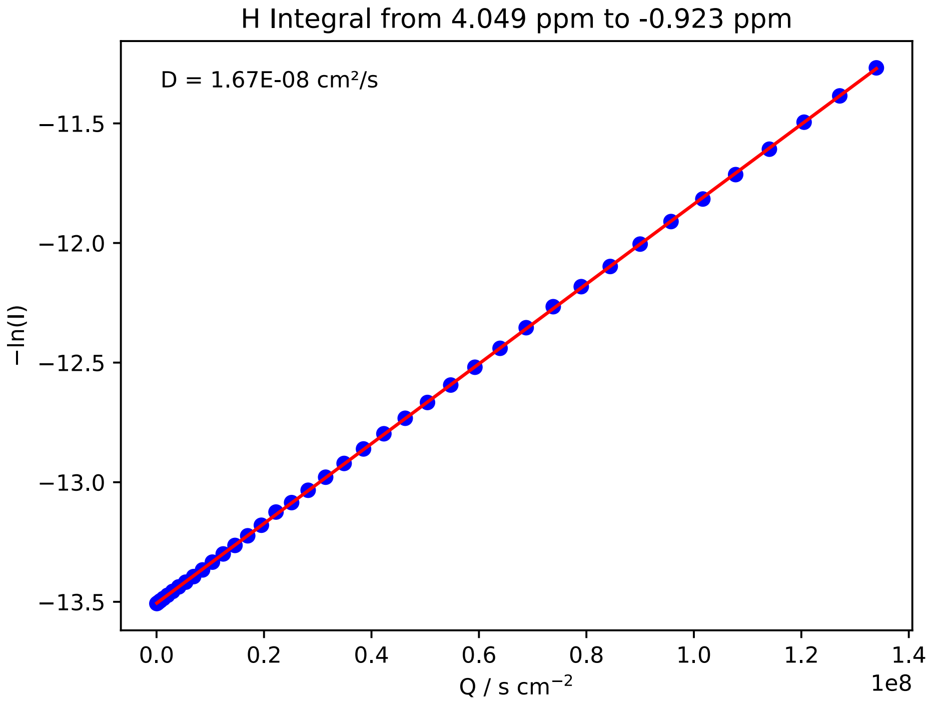

Figure S22: $[P_{8881}]$[1,2,4-triazolate] after saturation with CO_2, ^{1}H PFGSTE experiment, remaining signals.

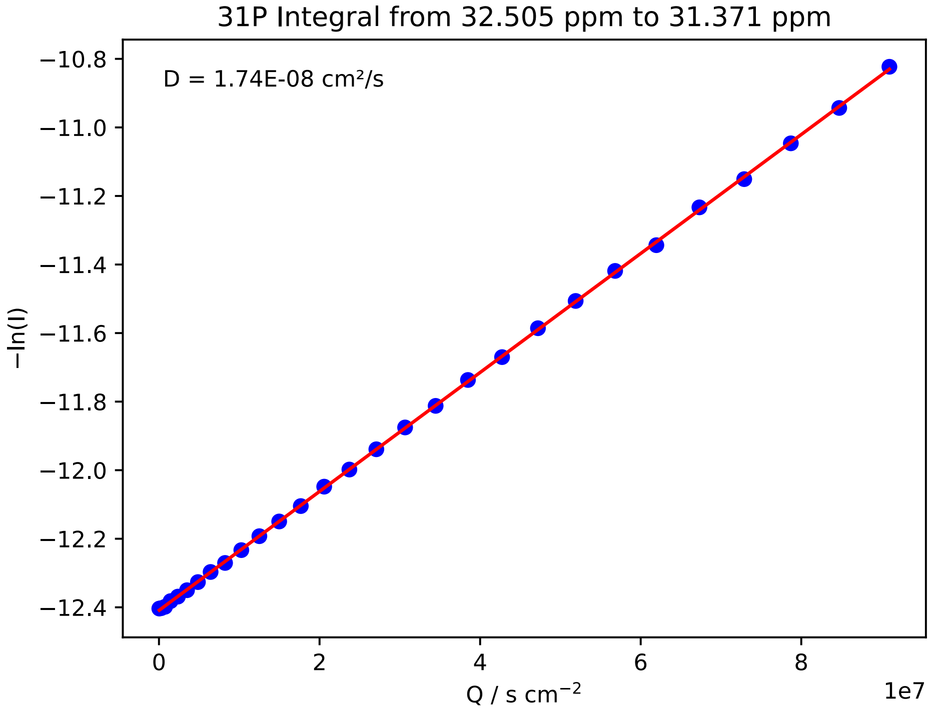

Figure S23: $[P_{8881}]$[1,2,4-triazolate] after saturation with CO_2, ^{31}P PFGSTE experiment, cation signal.

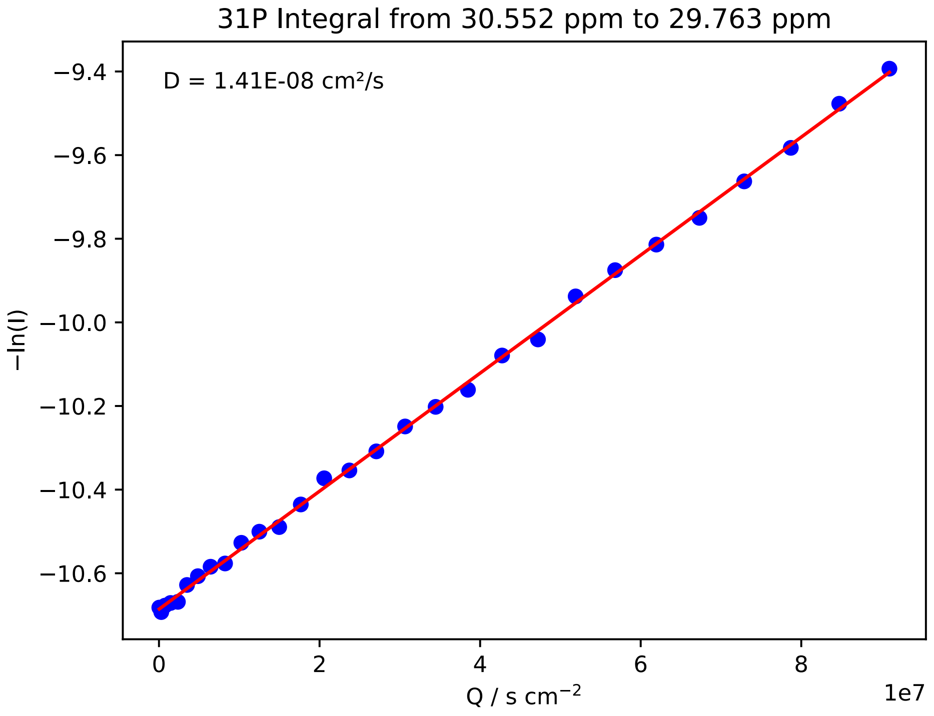

Figure S24: $[P_{8881}]$[1,2,4-triazolate] after saturation with CO_2, ^{31}P PFGSTE experiment, carboxylate species (octyl chain).

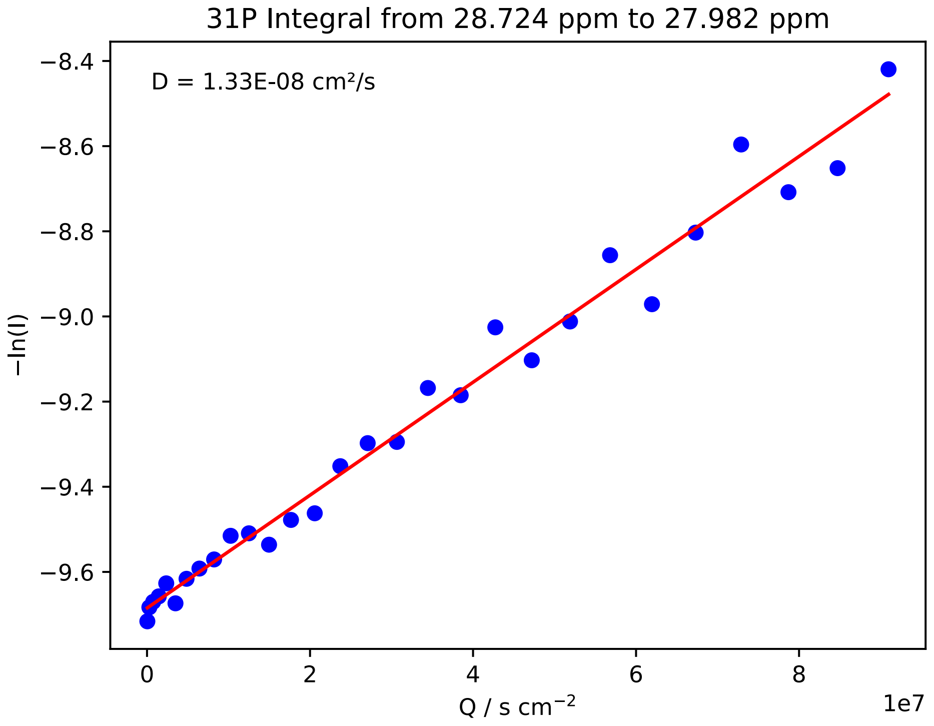

Figure S25: $[P_{8881}]$[1,2,4-triazolate] after saturation with CO_2, ^{31}P PFGSTE experiment, carboxylate species (methyl group).

10. Kinetics of CO_2 absorption *via* NMR

The formation of the zwitterionic carboxylate is slow at 30°C. To demonstrate this, we have kept a sample of neat [P_{8881}][triaz] at 30°C in an NMR tube pressurised with 2 bar CO_2, Figure S26. After three hours, the peaks in the phosphorus NMR corresponding to the two possible zwitterions begin to appear. However, the peak integrals suggest that only about 0.5% of zwitterions are present, even though the reaction of the anion to the carbamate reaches equilibrium at this time scale. Over the course of 24 h, the content of zwitterionic carboxylate species increases to 8-9%, and stabilises at ≈12% after several days. For comparison, the extent of reaction obtained from the fit of the isotherms suggests a carboxylate content of 13% under these conditions, hence this NMR measurement is in excellent agreement with the composition deduced from the gravimetric gas absorption measurement.

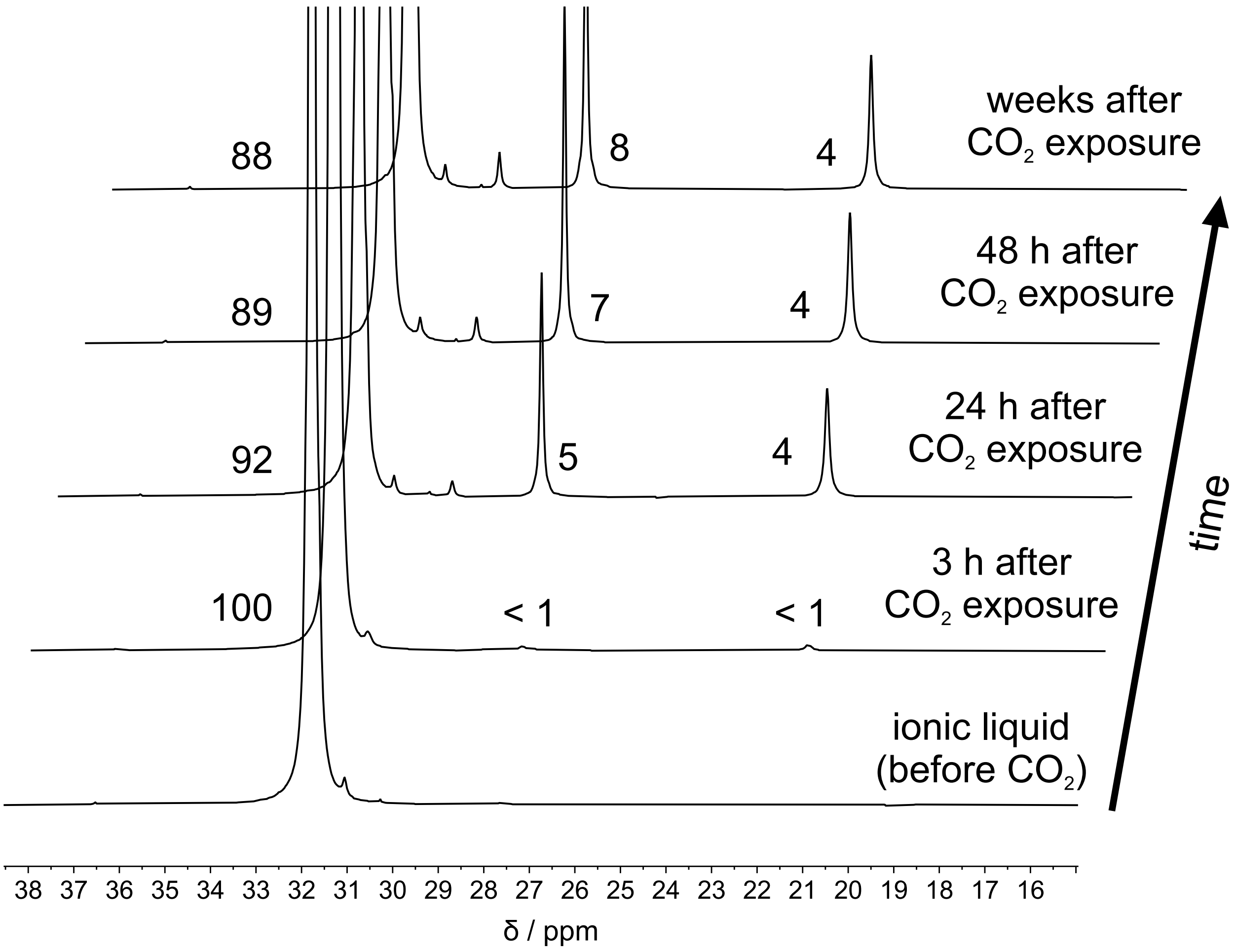

Figure S26: $^{31}P\{^1H\}$ NMR of neat [P_{8881}][triaz], inverse gated decoupling with 5 s relaxation delay plus 2 s acquisition time, before and after exposure to 2 bar of CO_2. The numbers give the relative integrals of the peaks for the three species shown here.

11. X-ray Scattering

Small-angle X-ray scattering experiments were performed on a Xeuss 3.0 diffractometer (Xenocs, Grenoble, France) with an X-ray wavelength of 1.54189 Å and an EIGER2 Si 1M detector (Dectris, Baden-Daettwil, Switzerland). The exposure time was 1 hour, and the sample-detector distance was determined by calibration with a silver behenate reference. The detector distance was 100 mm for all samples except the ZIF-8 / ionic liquid mixture after treatment with CO2, for which the detector distance was 102 mm. Borosilicate glass capillaries with outer diameter of 1.5 mm, 80 mm length and 0.01 mm wall thickness (WJM-Glas, Berlin, Germany) were used to contain the samples. Each capillary was filled with the sample and sealed with hot glue inside a glovebox. The resulting SAXS patterns are given in Figure S27 to Figure S30.

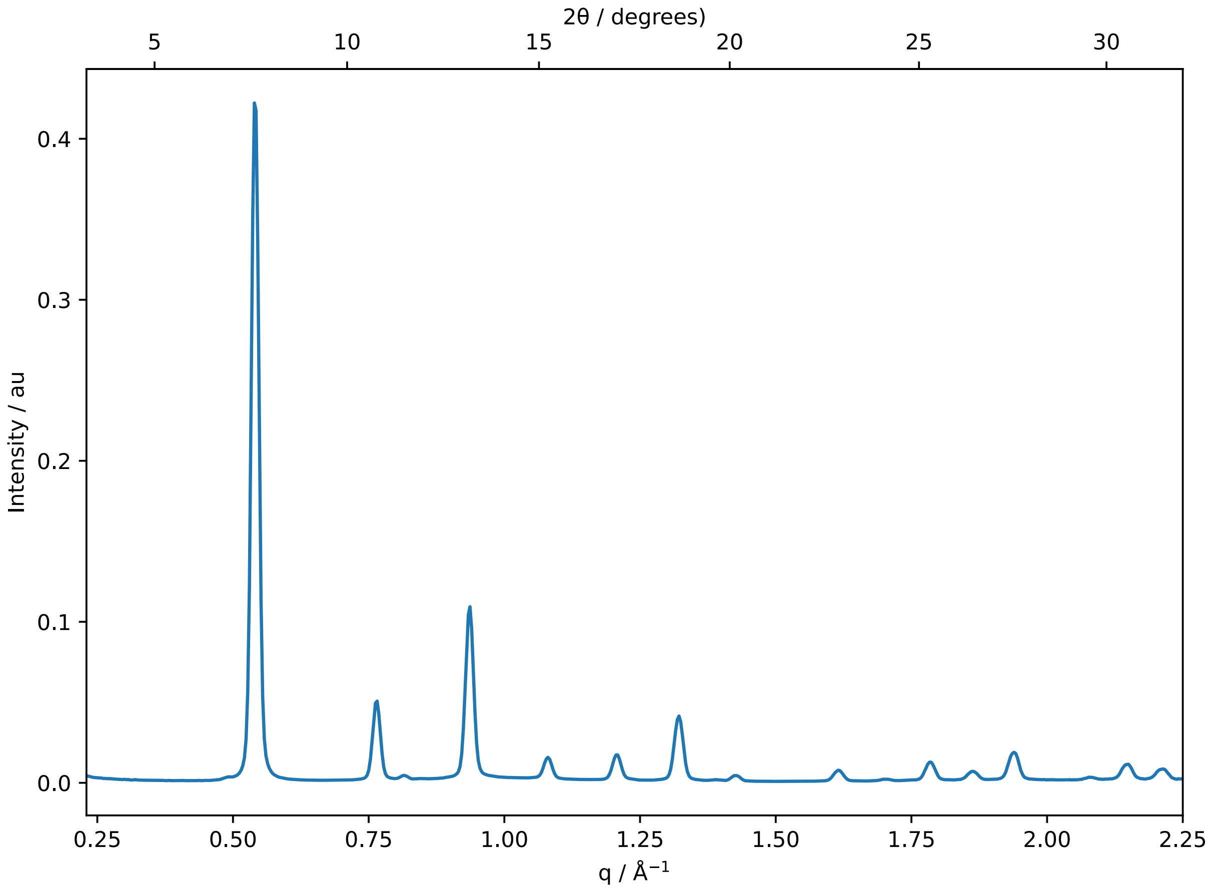

Figure S27: Radially averaged SAXS data for the ZIF-8 powder processed as described in the synthesis section.

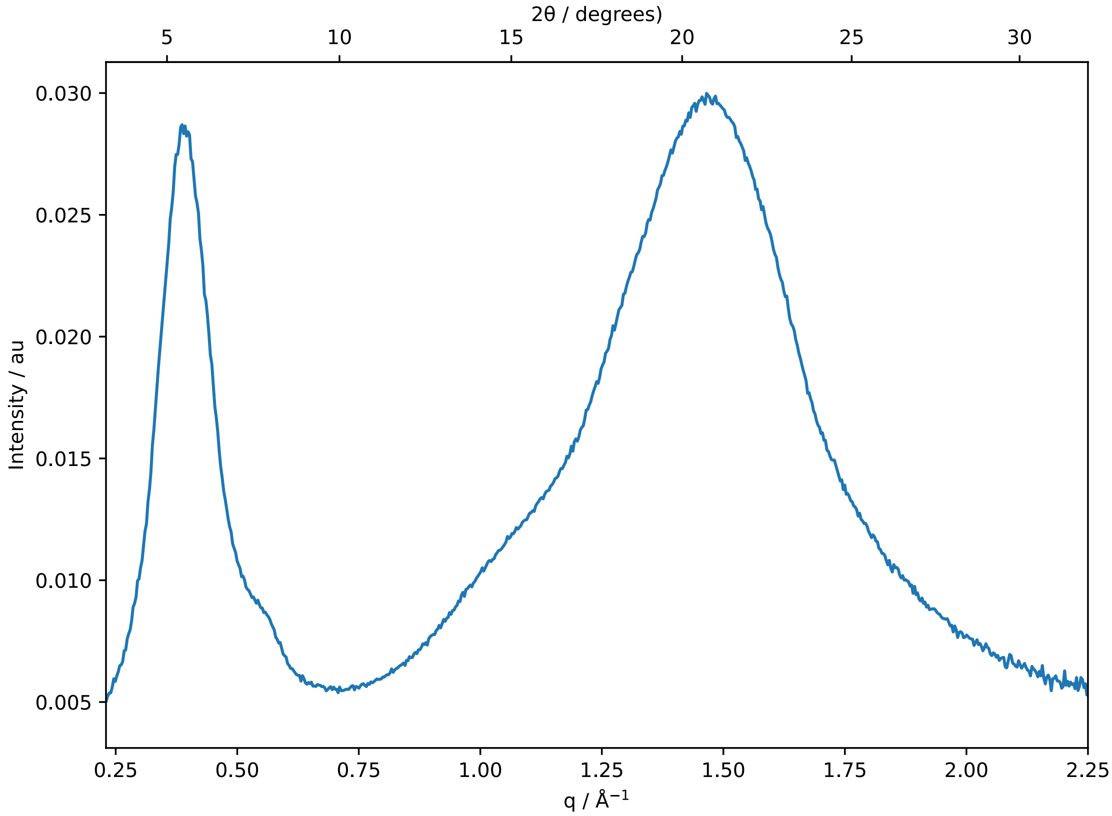

Figure S28: Radially averaged SAXS data for the [P_{8881}][triazolate] ionic liquid without added ZIF-8.

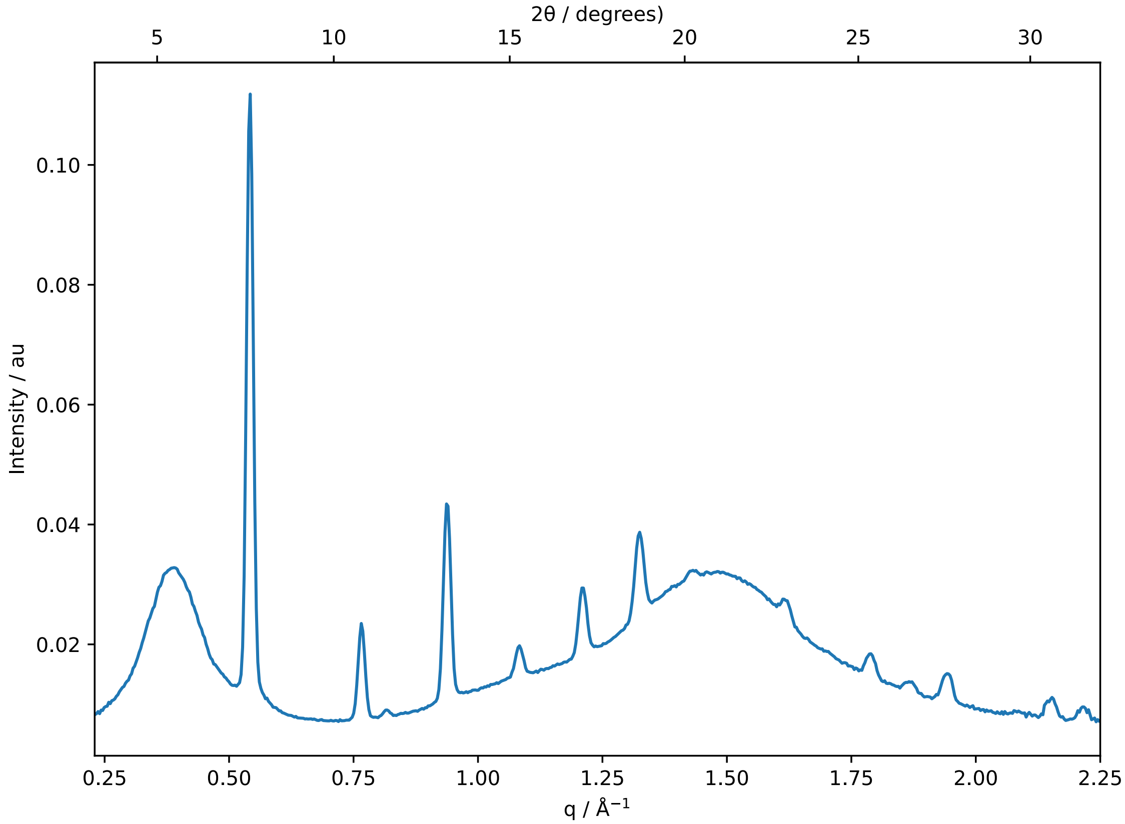

Figure S29: Radially averaged SAXS data for the [P_{8881}][triazolate] ionic liquid with added ZIF-8 (porous ionic liquid PIL) before treatment with CO_2.

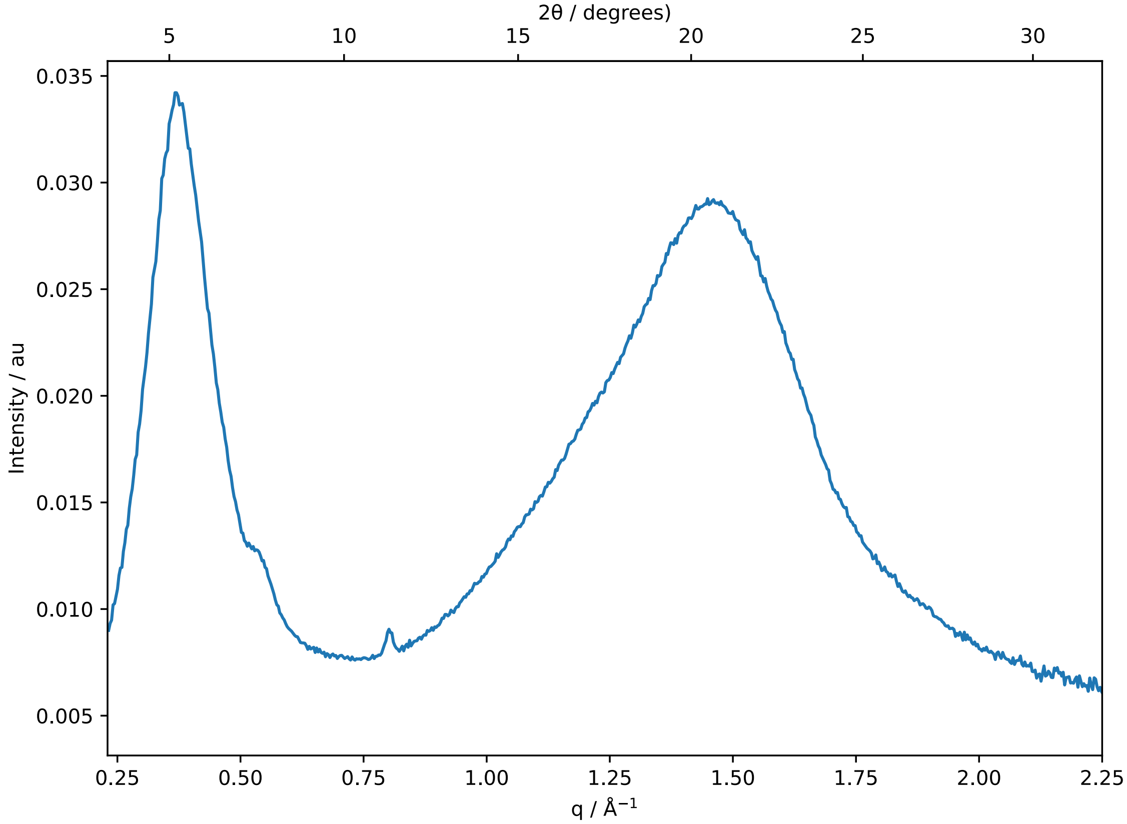

Figure S30: Radially averaged SAXS data for the [P_{8881}][triazolate] ionic liquid with added ZIF-8 after treatment with CO_2. The ZIF-8 solid phase has dissolved completely. The small peak around 2θ=11.4° is due to a small amount of impurity resulting from partial hydrolysis of the original ZIF-8 surface.

12. *Ab initio* calculations

Ab initio calculations were performed using the Gaussian 16 software package, revision C.01.[12] All calculations were performed in a [C_4C_1Im][NTf_2] SMD environment.[13] We used a pruned integration grid ('int=ultrafine' in gaussian) with 99 radial shells and 690 angular points per shell. Initial geometry optimisations were performed at the B3LYP-GD3BJ/6-311+G(d,p) level of theory with geometry convergence criteria (in atomic units, 'verytight' in gaussian) of 2×10^{-6} on maximum force, 1×10^{-6} on RMS force, 6×10^{-6} on maximum displacement, and 4×10^{-6} on RMS displacement.[14,15] Stationary points were confirmed as minima through frequency analysis. MP2(full)/cc-pVTZ single point energies were calculated using the optimised geometry. SCF convergence criteria were 10^{-10} / 10^{-11} RMS change in the density matrix and 10^{-8} / 10^{-9} maximum change in the density matrix for the DFT/MP2 levels of theory, respectively. Optimised geometries can be found in the accompanying files in xyz format. The resulting reaction energies are summarised in Table S11.

Table S11: Reaction energies in kJ mol^{-1}. The column labelled MP2 contains the internal electronic energy at the MP2(full)/cc-pVTZ// B3LYP-GD3BJ/6-311+G(d,p) level of theory without any thermal corrections. The last two columns contain the thermal free energies and enthalpies, respectively, at the B3LYP-GD3BJ/6-311+G(d,p) level of theory within the rigid rotator / harmonic oscillator approximations and including zero point energies.

Reaction	MP2	ΔG	ΔH
1-COOH-triazolate → H^+ + [1-CO_2-triazolate]$^-$	674	597	630
[1H-triazolium]$^+$ → H^+ + triazole	591	537	568
[4H-triazolium]$^+$ → H^+ + triazole	630	571	603
triazole → H^+ + [triazolate]$^-$	753	685	716
[P1112]$^+$ → H^+ + P1112-ylide	859	795	829
[P1112-COOH]$^+$ → H^+ + P1112-COO	692	609	644
[P1111]$^+$ → H^+ + P1111-ylide	842	779	811
[P1111-COOH]$^+$ → H^+ + P1111-COO	692	608	643
4-COOH-triazolate → H^+ + [4-CO_2-triazolate]$^-$	669	592	624
[2-methylimidazolium]$^+$ → H^+ + 2-methylimidazole	679	622	653
2-methylimidazole → H^+ + [2-methylimidazolate]$^-$	782	719	751
1-CO_2-2-methylimidazolium → H^+ + [1-CO_2-2-methylimidazolate]$^-$	715	655	685
[H_3O]$^+$ → H^+ + H_2O	540	464	493
H_2O → H^+ + [HO]$^-$	916	776	803
CO_2 +[P1112-ylide] → [P1112-COO]	−155	−119	−167
CO_2 +[P1111-ylide] → [P1111-COO]	−130	−106	−148
CO_2 +[triazolate]$^-$ → [1-CO_2-triazolate]$^-$	−48	−14	−58
CO_2 +[triazolate]$^-$ → [4-CO_2-triazolate]$^-$	−38	−5.9	−50
CO_2 +[2-methylimidazolate]$^-$ → [1-CO_2-2-methylimidazolate]$^-$	−73	−35	−84

13. References

1 D. H. Vogel, *Phys. Zeitschrift*, 1921, **22**, 645.

2 G. Tammann and W. Hesse, *Zeitschrift für Anorg. und Allg. Chemie*, 1926, **156**, 245–257.

3 G. S. Fulcher, *J. Am. Ceram. Soc.*, 1925, **8**, 339–355.

4 J. Avila, C. Červinka, P. Dugas, A. A. H. Pádua and M. Costa Gomes, *Adv. Mater. Interfaces*, 2021, **8**, 2001982.

5 M. Frenkel and K. N. Marsh, Eds., *Virial Coefficients of Pure Gases*, Springer-Verlag, Berlin/Heidelberg, 2002, vol. 21A.

6 R. Sander, W. E. Acree, A. De Visscher, S. E. Schwartz and T. J. Wallington, *Pure Appl. Chem.*, 2022, **94**, 71–85.

7 L. F. Lepre, J. Szala-Bilnik, L. Pison, M. Traïkia, A. A. H. Pádua, R. A. Ando and M. F. Costa Gomes, *Phys. Chem. Chem. Phys.*, 2017, **19**, 12431–12440.

8 N. Scaglione, J. Avila, A. A. H. Padua and M. Costa Gomes, *Faraday Discuss.*, 2024, **178**, 178.

9 E. Zorębski, M. Zorębski, M. Dzida, P. Goodrich and J. Jacquemin, *Ind. Eng. Chem. Res.*, 2017, **56**, 2592–2606.

10 D. A. Ditmars, S. Ishihara, S. S. Chang, G. Bernstein and E. D. West, *J. Res. Natl. Bur. Stand. (1934).*, 1982, **87**, 159.

11 D. H. Wu, A. D. Chen and C. S. Johnson, *J. Magn. Reson. Ser. A*, 1995, **115**, 260–264.

12 Gaussian 09, Revision E.01, M. J. Frisch, G. W. Trucks, H. B. Schlegel, G. E. Scuseria, M. A. Robb, J. R. Cheeseman, G. Scalmani, V. Barone, B. Mennucci, G. A. Petersson, H. Nakatsuji, M. Caricato, X. Li, H. P. Hratchian, A. F. Izmaylov, J. Bloino, G. Zheng, J. L. Sonnenberg, M. Hada, M. Ehara, K. Toyota, R. Fukuda, J. Hasegawa, M. Ishida, T. Nakajima, Y. Honda, O. Kitao, H. Nakai, T. Vreven, J. A. Montgomery, Jr., J. E. Peralta, F. Ogliaro, M. Bearpark, J. J. Heyd, E. Brothers, K. N. Kudin, V. N. Staroverov, T. Keith, R. Kobayashi, J. Normand, K. Raghavachari, A. Rendell, J. C. Burant, S. S. Iyengar, J. Tomasi, M. Cossi, N. Rega, J. M. Millam, M. Klene, J. E. Knox, J. B. Cross, V. Bakken, C. Adamo, J. Jaramillo, R. Gomperts, R. E. Stratmann, O. Yazyev, A. J. Austin, R. Cammi, C. Pomelli, J. W. Ochterski, R. L. Martin, K. Morokuma, V. G. Zakrzewski, G. A. Voth, P. Salvador, J. J. Dannenberg, S. Dapprich, A. D. Daniels, O. Farkas, J. B. Foresman, J. V. Ortiz, J. Cioslowski, and D. J. Fox, Gaussian, Inc., Wallingford CT, 2013.

13 V. S. Bernales, A. V. Marenich, R. Contreras, C. J. Cramer and D. G. Truhlar, *J. Phys. Chem. B*, 2012, **116**, 9122–9129.

14 S. Grimme, J. Antony, S. Ehrlich and H. Krieg, *J. Chem. Phys.*, 2010, **132**, 154104.

15 S. Grimme, S. Ehrlich and L. Goerigk, *J. Comput. Chem.*, 2011, **32**, 1456–1465.
