# Supplementary figures and images for: Can Gas Absorption be Tuned in a Multifunctional Ionic Liquid?
Source: ChemSusChem. 2025 Sep 2;18(20):e202501347. doi: 10.1002/cssc.202501347 (PMC12548951; doi:10.1002/cssc.202501347)

# Empty Pan Data at T = 30.00 °C

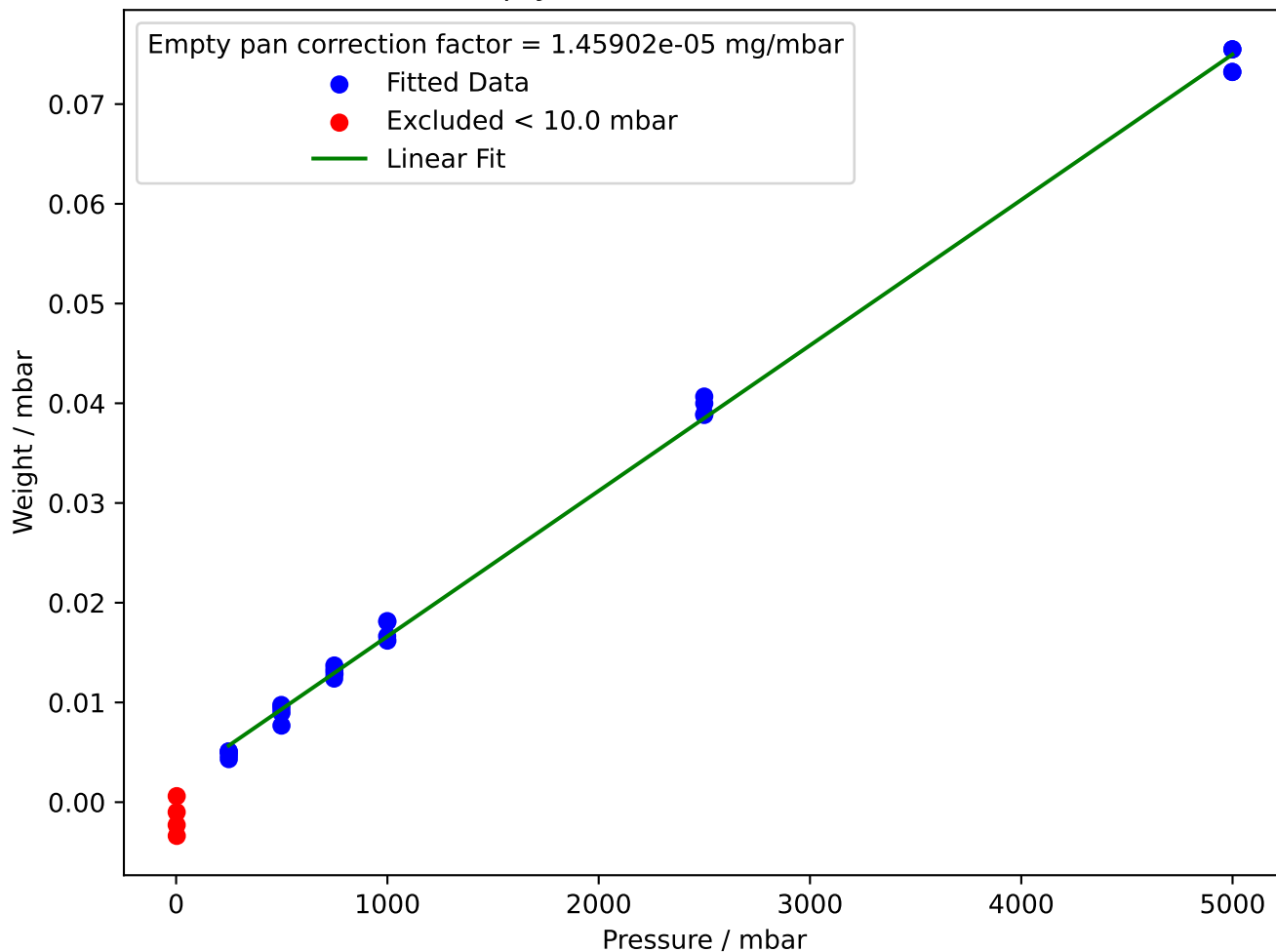

Supplement: Supplementary file 1 — Supplementary Material [file CSSC-18-e202501347-s001.zip › Microbalance_data_analysis/P8881triaz+ZIF8-CH4-after-CO2-treatment/empty_pan_plot_30.0C.pdf]

Absorbed gas at  $T = 30.0^{\circ}\text{C}$

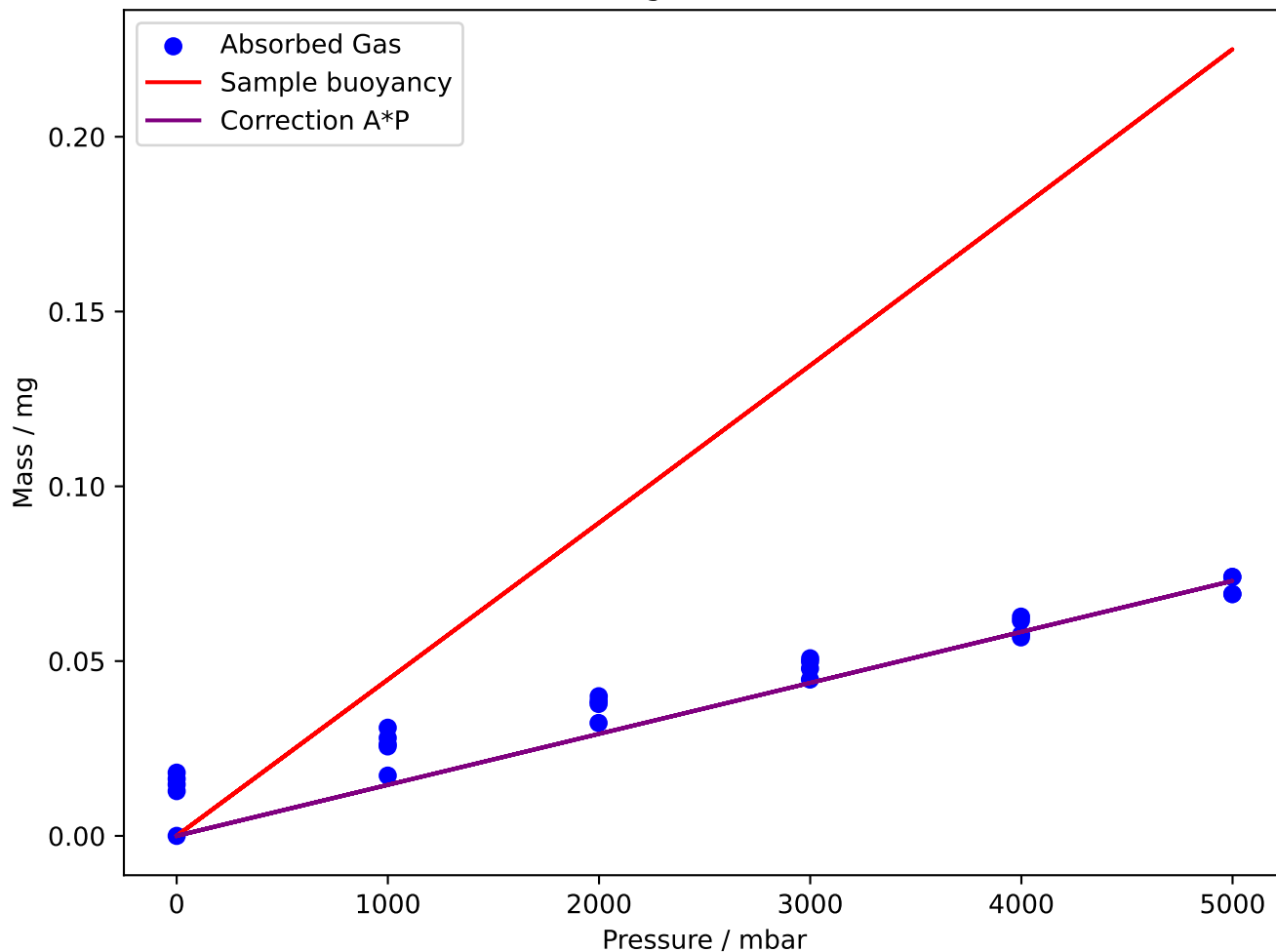

Supplement: Supplementary file 1 — Supplementary Material [file CSSC-18-e202501347-s001.zip › Microbalance_data_analysis/P8881triaz+ZIF8-CH4-after-CO2-treatment/measurement_plot_30.0C.pdf]

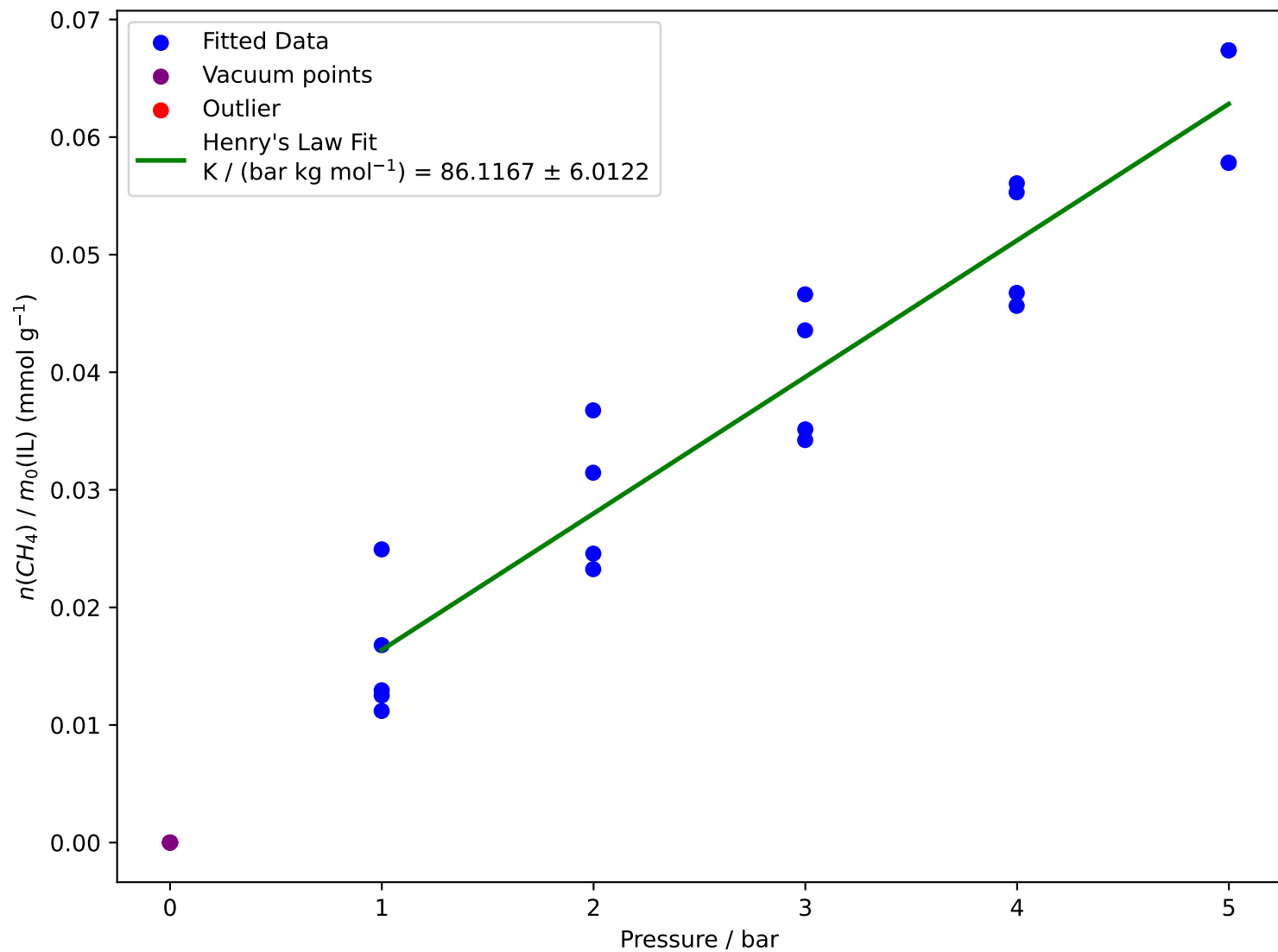

Supplement: Supplementary file 1 — Supplementary Material [file CSSC-18-e202501347-s001.zip › Microbalance_data_analysis/P8881triaz+ZIF8-CH4-after-CO2-treatment/molality_fit_30C.pdf]

# Empty Pan Data at T = 30.00 °C

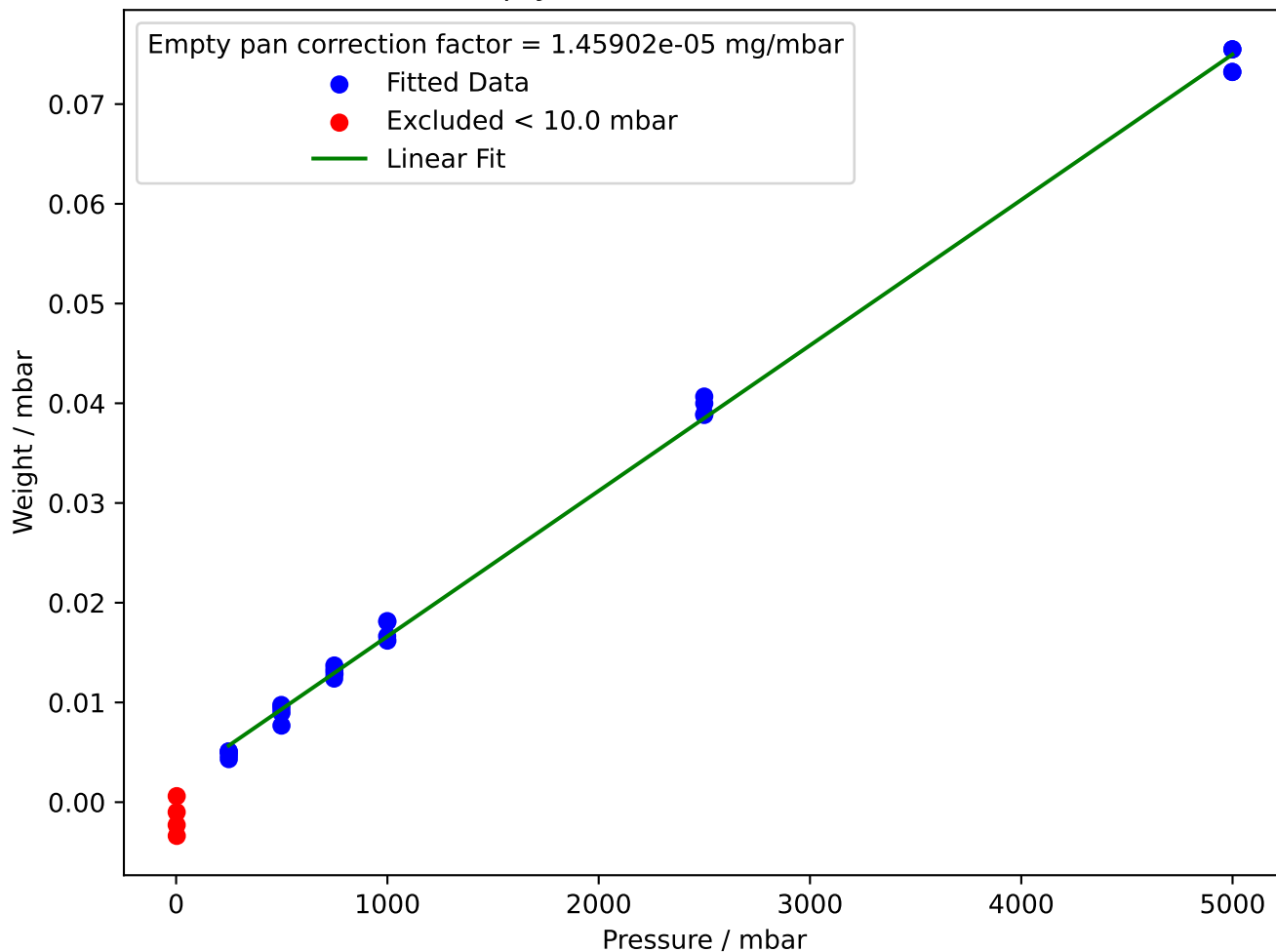

Supplement: Supplementary file 1 — Supplementary Material [file CSSC-18-e202501347-s001.zip › Microbalance_data_analysis/P8881triaz+ZIF8-CH4/empty_pan_plot_30.0C.pdf]

# Absorbed gas at $T = 30.0^{\circ}\text{C}$

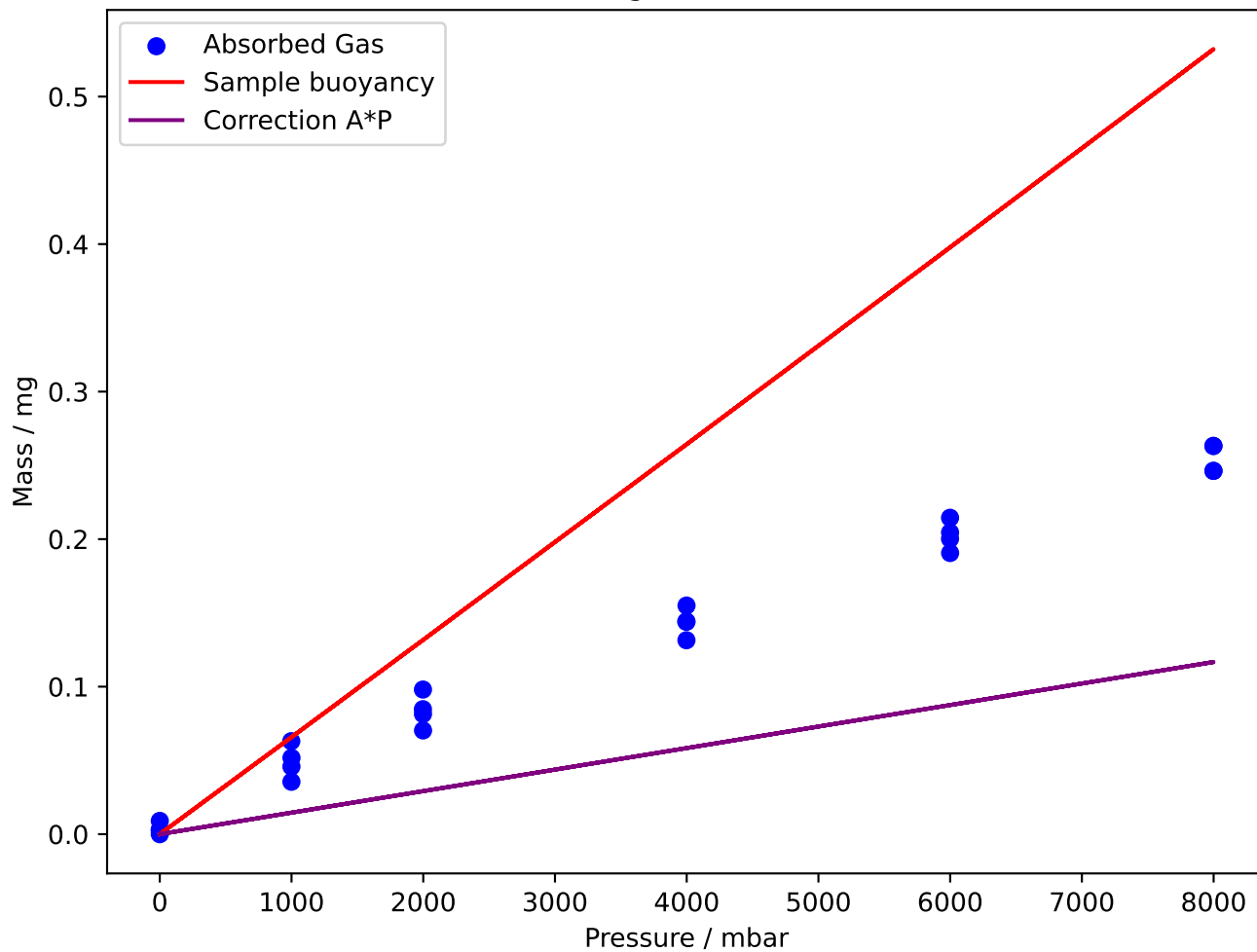

Supplement: Supplementary file 1 — Supplementary Material [file CSSC-18-e202501347-s001.zip › Microbalance_data_analysis/P8881triaz+ZIF8-CH4/measurement_plot_30.0C.pdf]

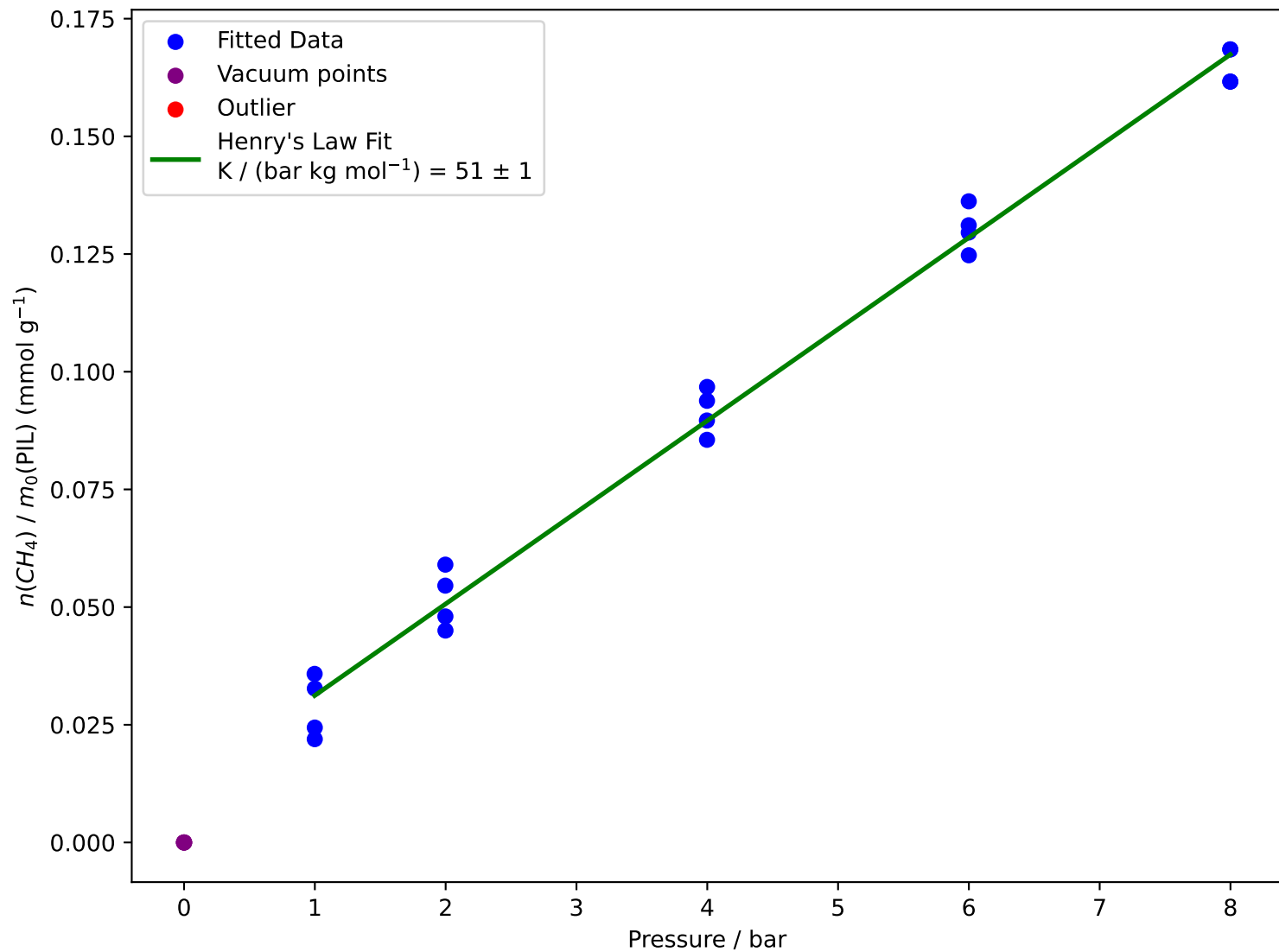

Supplement: Supplementary file 1 — Supplementary Material [file CSSC-18-e202501347-s001.zip › Microbalance_data_analysis/P8881triaz+ZIF8-CH4/molality_fit_30C.pdf]

# Empty Pan Data at T = 29.98 °C

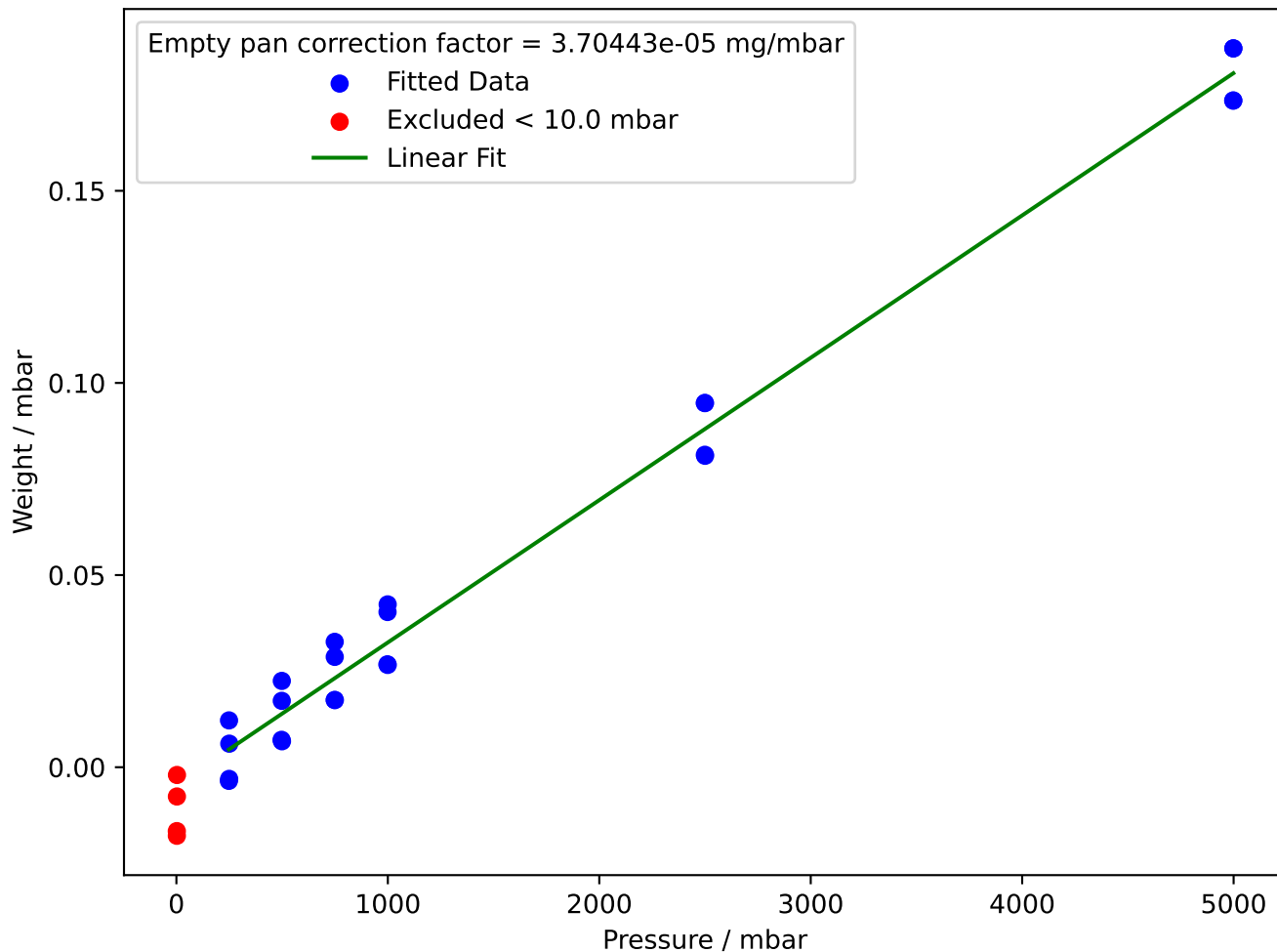

Supplement: Supplementary file 1 — Supplementary Material [file CSSC-18-e202501347-s001.zip › Microbalance_data_analysis/P8881triaz+ZIF8-CO2/empty_pan_plot_30.0C.pdf]

# Absorbed gas at T = 30.0°C

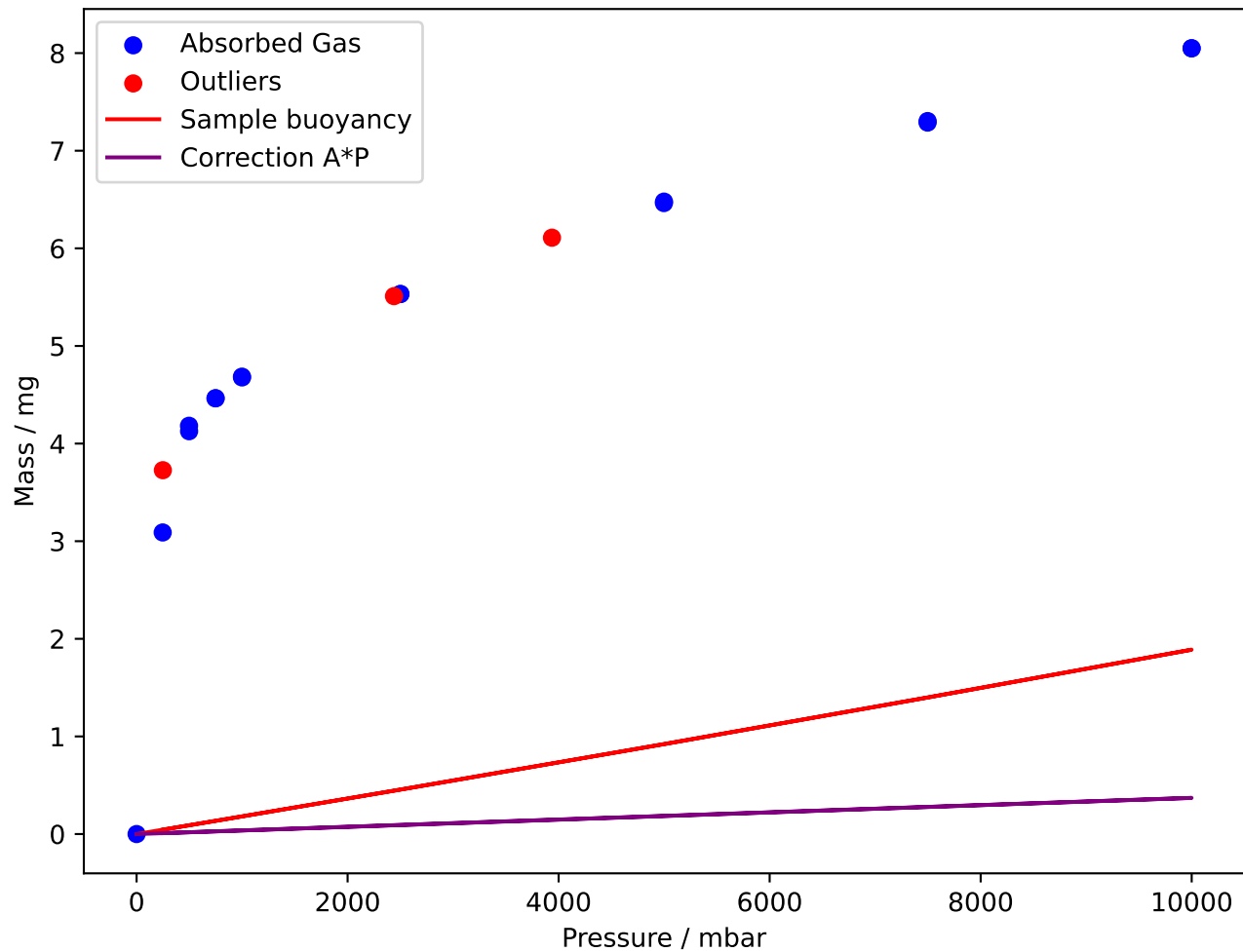

Supplement: Supplementary file 1 — Supplementary Material [file CSSC-18-e202501347-s001.zip › Microbalance_data_analysis/P8881triaz+ZIF8-CO2/measurement_plot_30.0C.pdf]

# Empty Pan Data at T = 30.00 °C

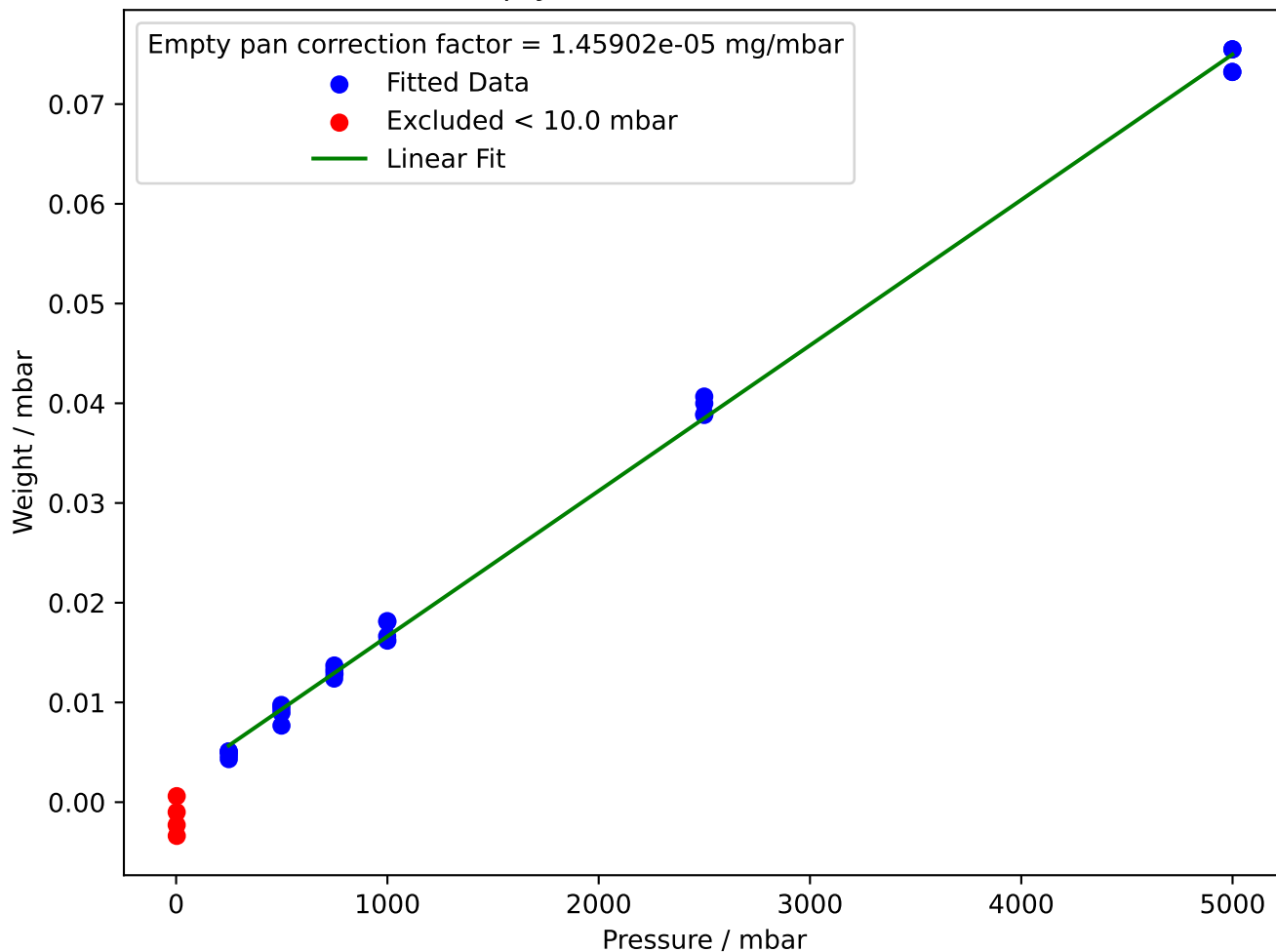

Supplement: Supplementary file 1 — Supplementary Material [file CSSC-18-e202501347-s001.zip › Microbalance_data_analysis/P8881triaz-CH4_1/empty_pan_plot_30.0C.pdf]

Absorbed gas at  $T = 30.0^{\circ}\text{C}$

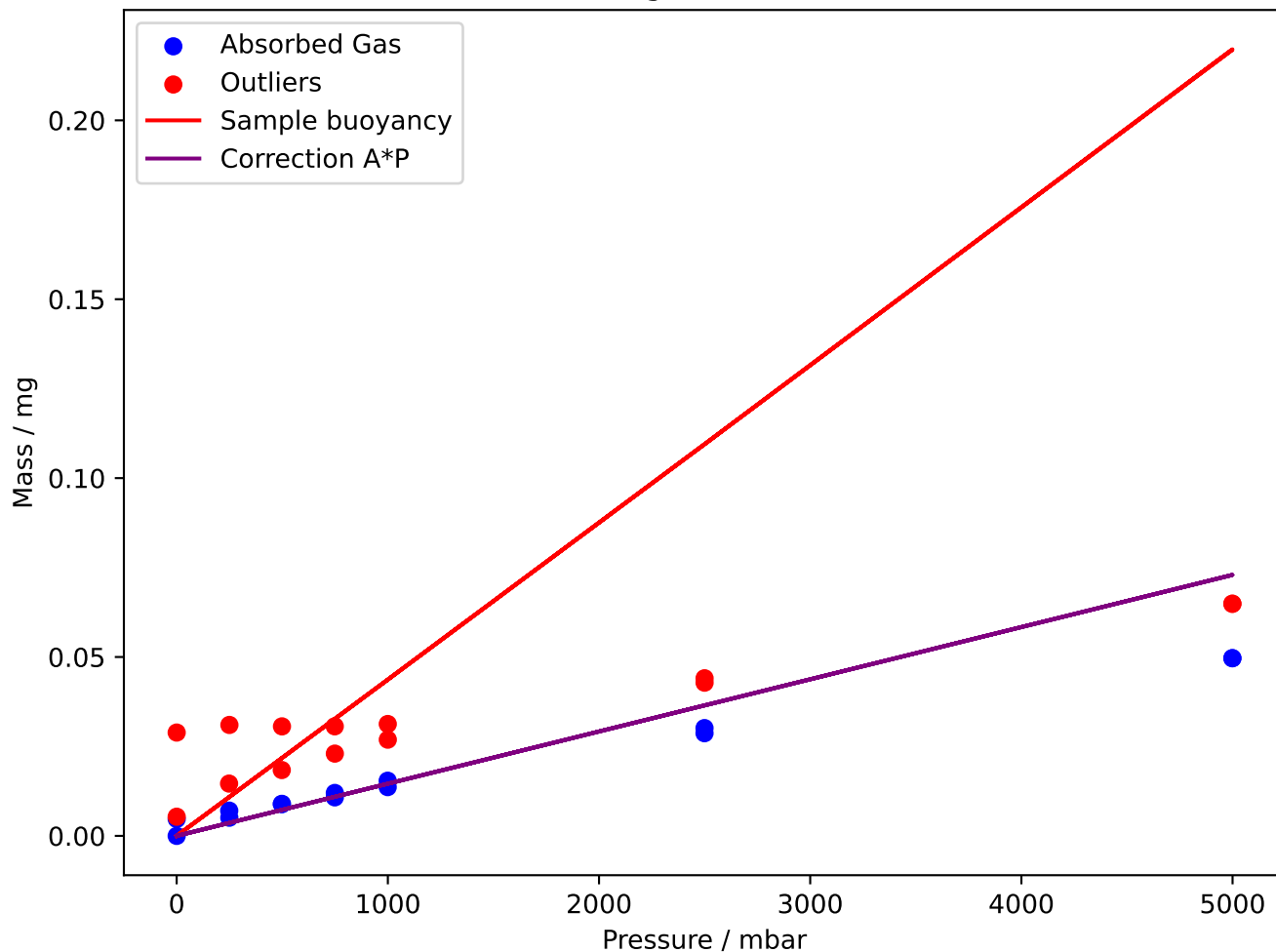

Supplement: Supplementary file 1 — Supplementary Material [file CSSC-18-e202501347-s001.zip › Microbalance_data_analysis/P8881triaz-CH4_1/measurement_plot_30.0C.pdf]

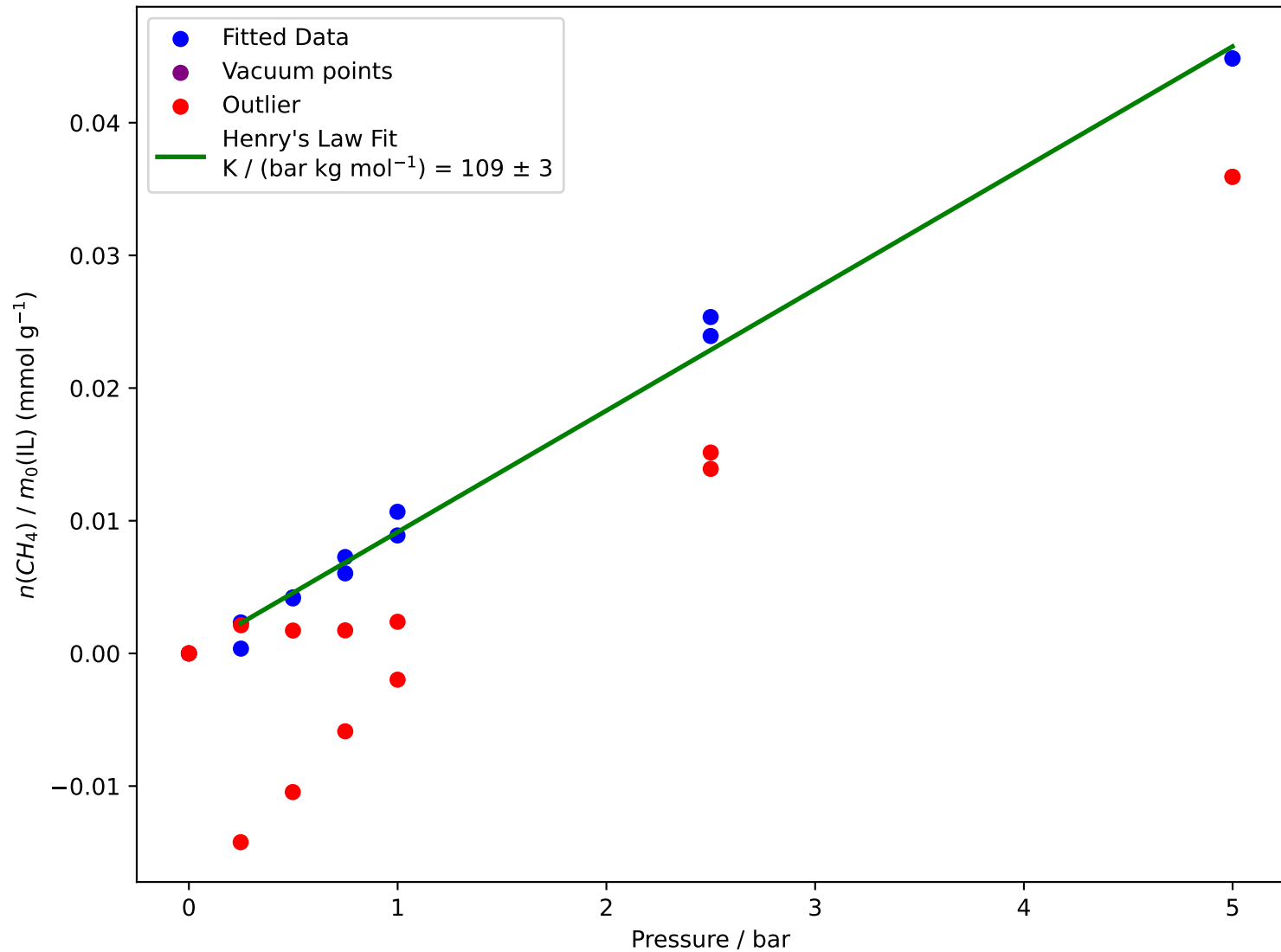

Supplement: Supplementary file 1 — Supplementary Material [file CSSC-18-e202501347-s001.zip › Microbalance_data_analysis/P8881triaz-CH4_1/molality_fit_30C.pdf]

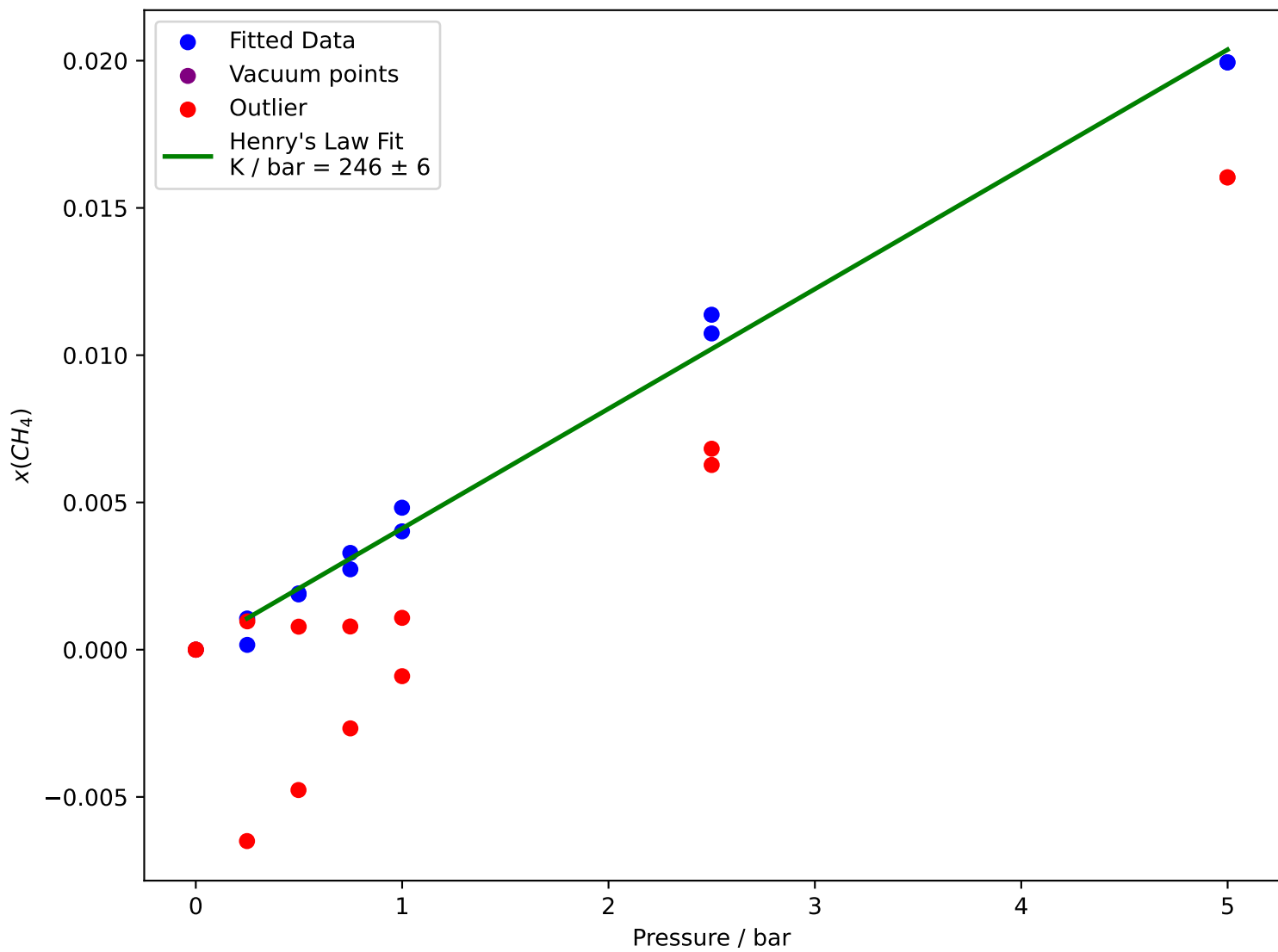

Supplement: Supplementary file 1 — Supplementary Material [file CSSC-18-e202501347-s001.zip › Microbalance_data_analysis/P8881triaz-CH4_1/molefraction_fit_30C.pdf]

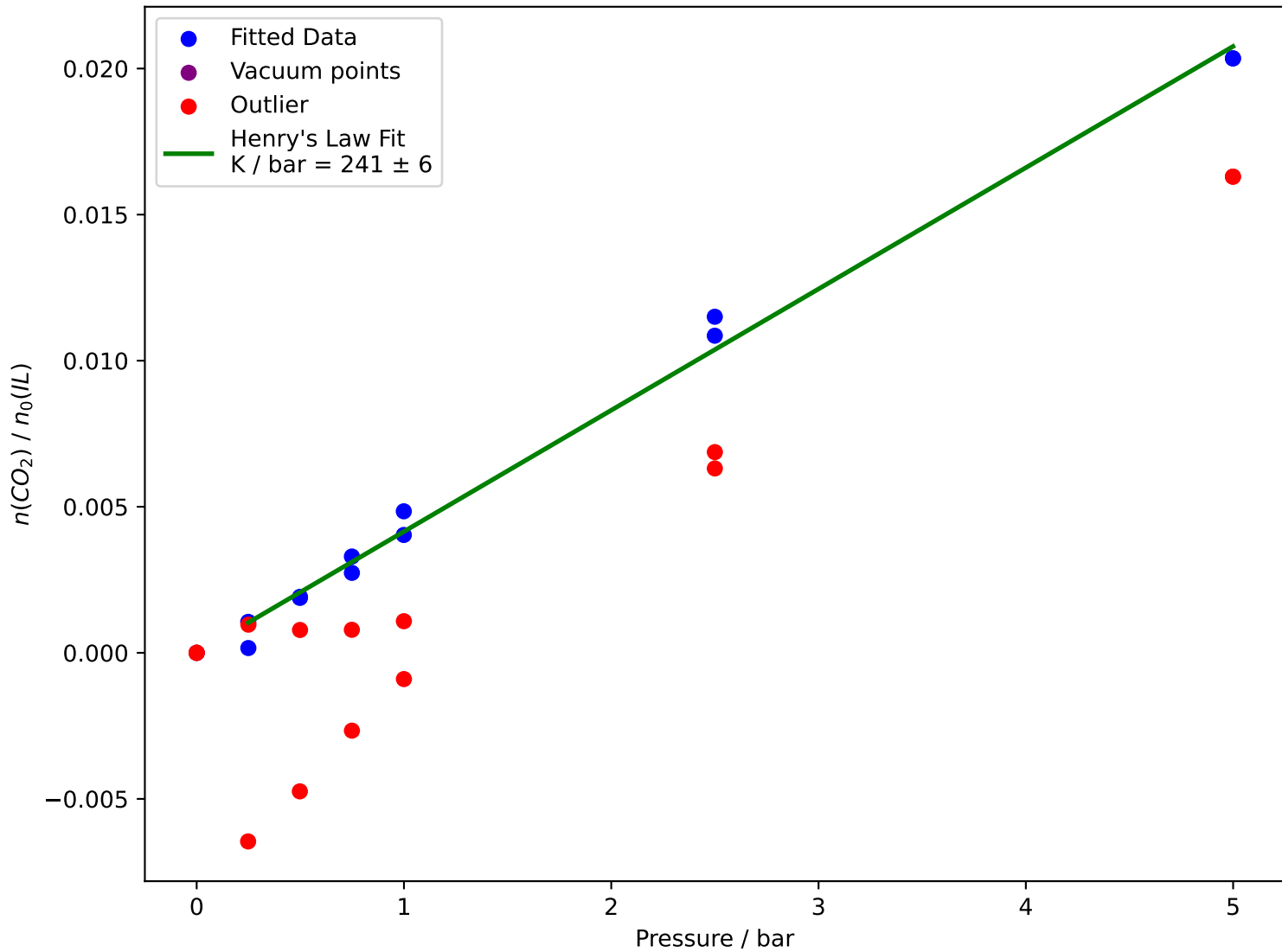

Supplement: Supplementary file 1 — Supplementary Material [file CSSC-18-e202501347-s001.zip › Microbalance_data_analysis/P8881triaz-CH4_1/moleratio_fit_30C.pdf]

# Empty Pan Data at T = 70.15 °C

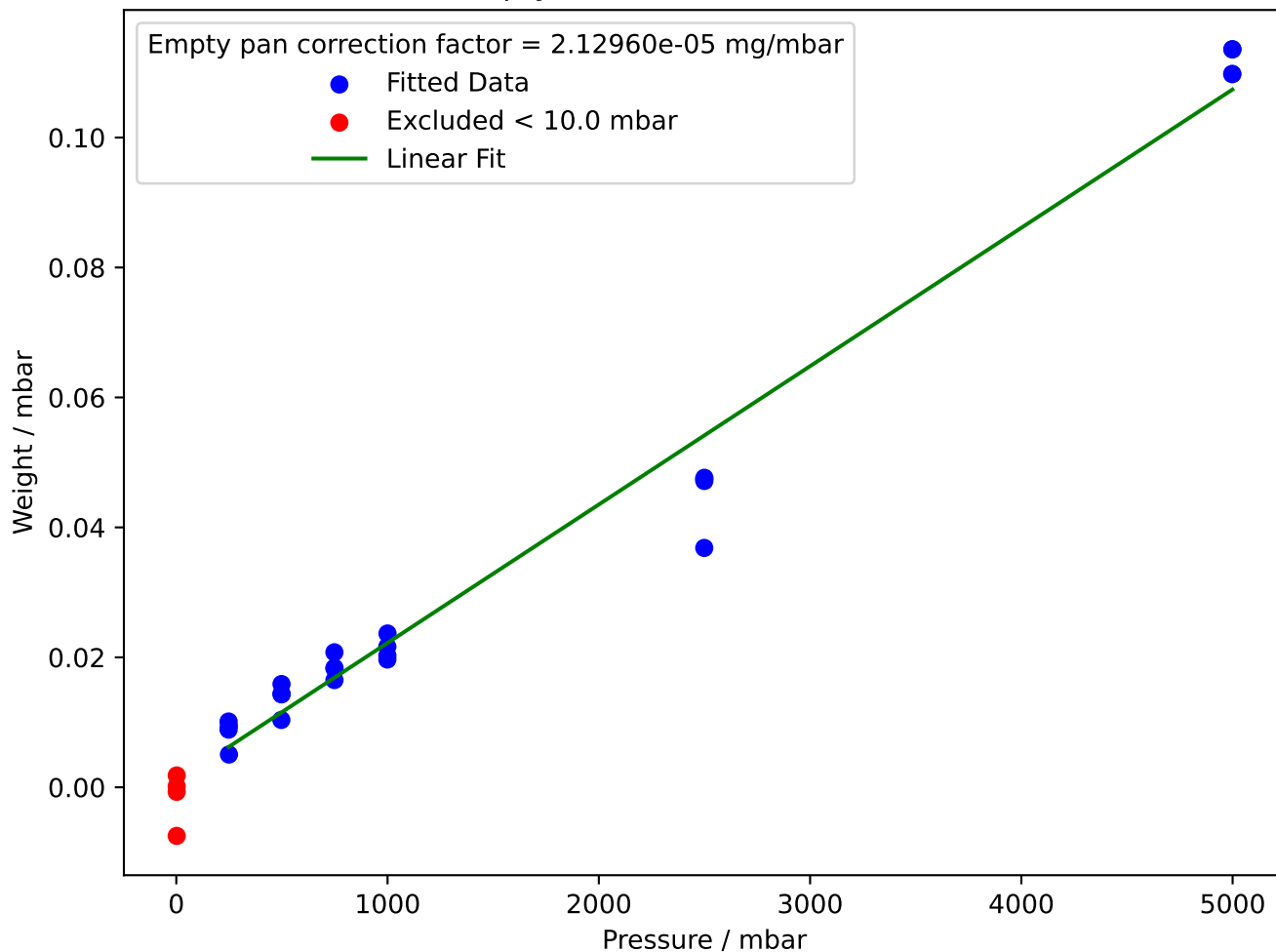

Supplement: Supplementary file 1 — Supplementary Material [file CSSC-18-e202501347-s001.zip › Microbalance_data_analysis/P8881triaz-CH4_2/empty_pan_plot_70.0C.pdf]

# Absorbed gas at T = 70.0°C

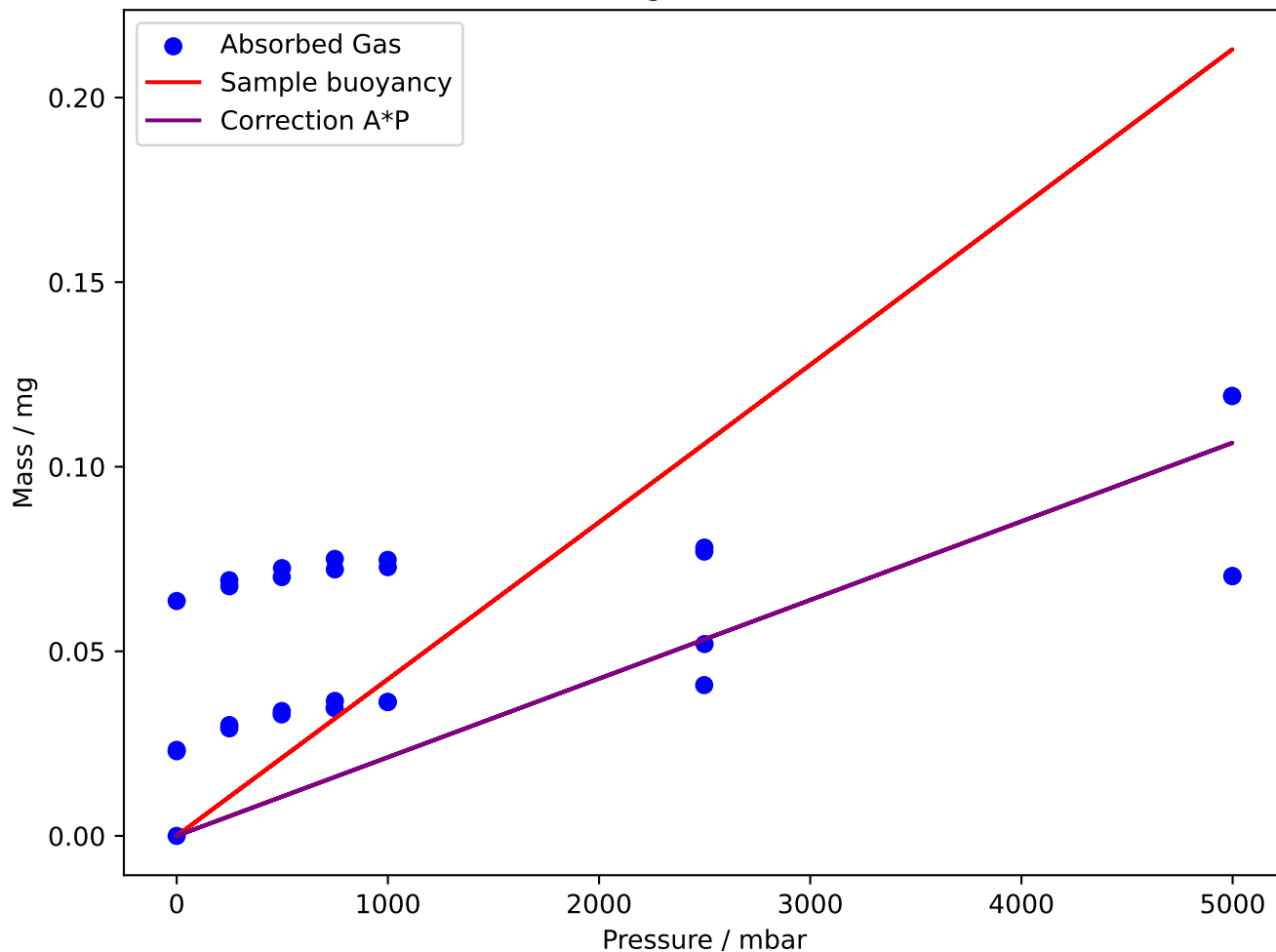

Supplement: Supplementary file 1 — Supplementary Material [file CSSC-18-e202501347-s001.zip › Microbalance_data_analysis/P8881triaz-CH4_2/measurement_plot_70.0C.pdf]

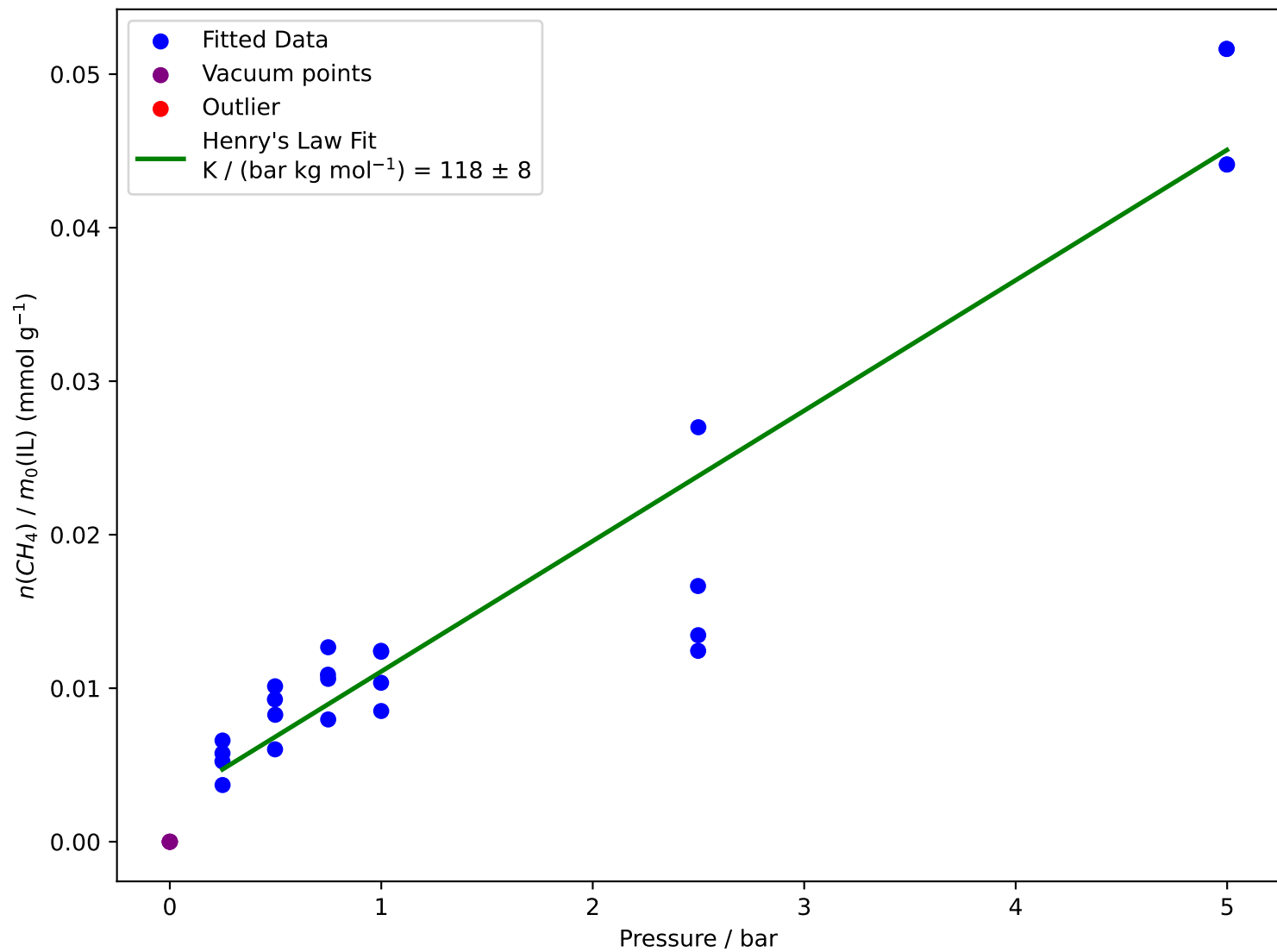

Supplement: Supplementary file 1 — Supplementary Material [file CSSC-18-e202501347-s001.zip › Microbalance_data_analysis/P8881triaz-CH4_2/molality_fit_70C.pdf]

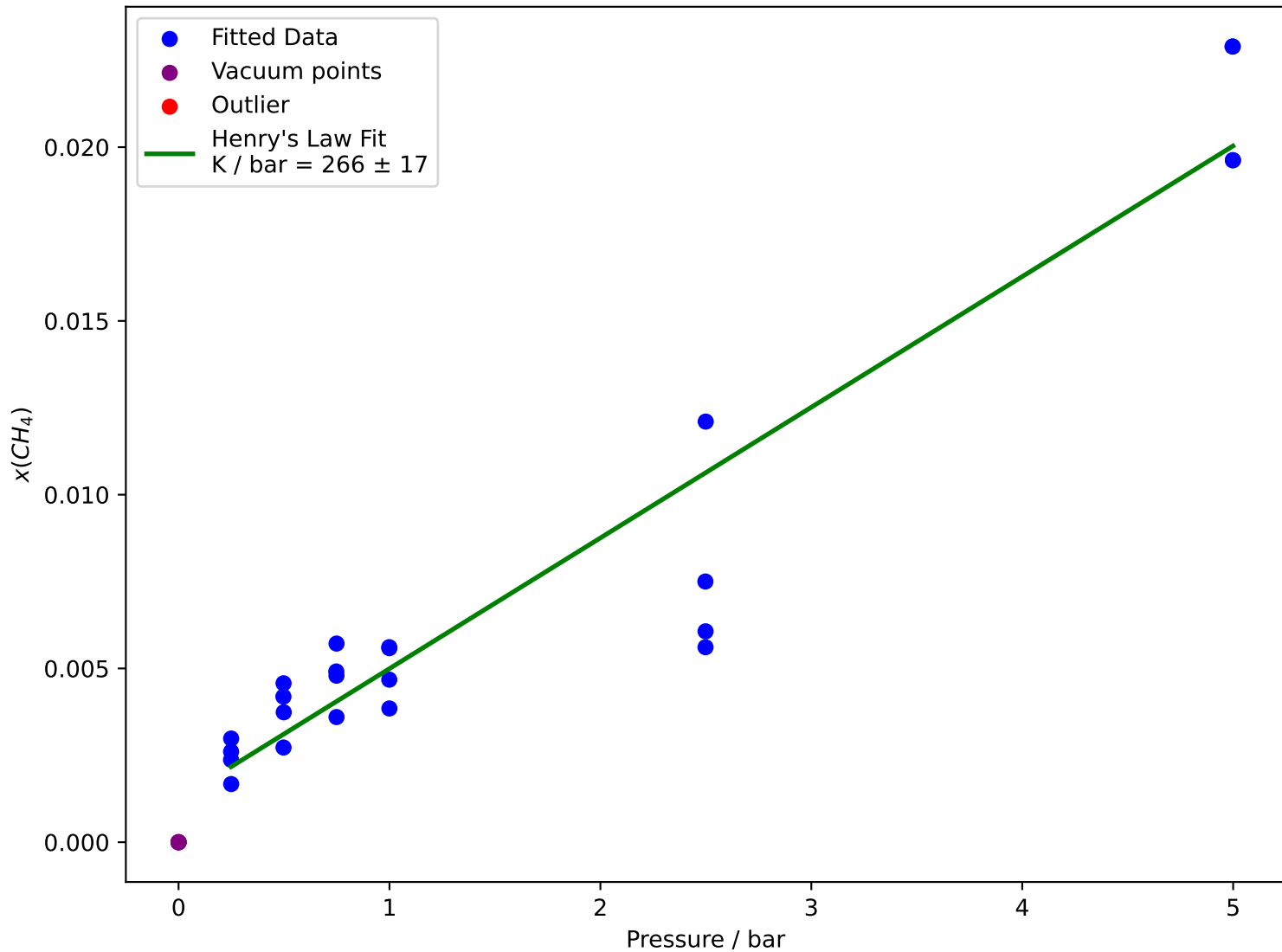

Supplement: Supplementary file 1 — Supplementary Material [file CSSC-18-e202501347-s001.zip › Microbalance_data_analysis/P8881triaz-CH4_2/molefraction_fit_70C.pdf]

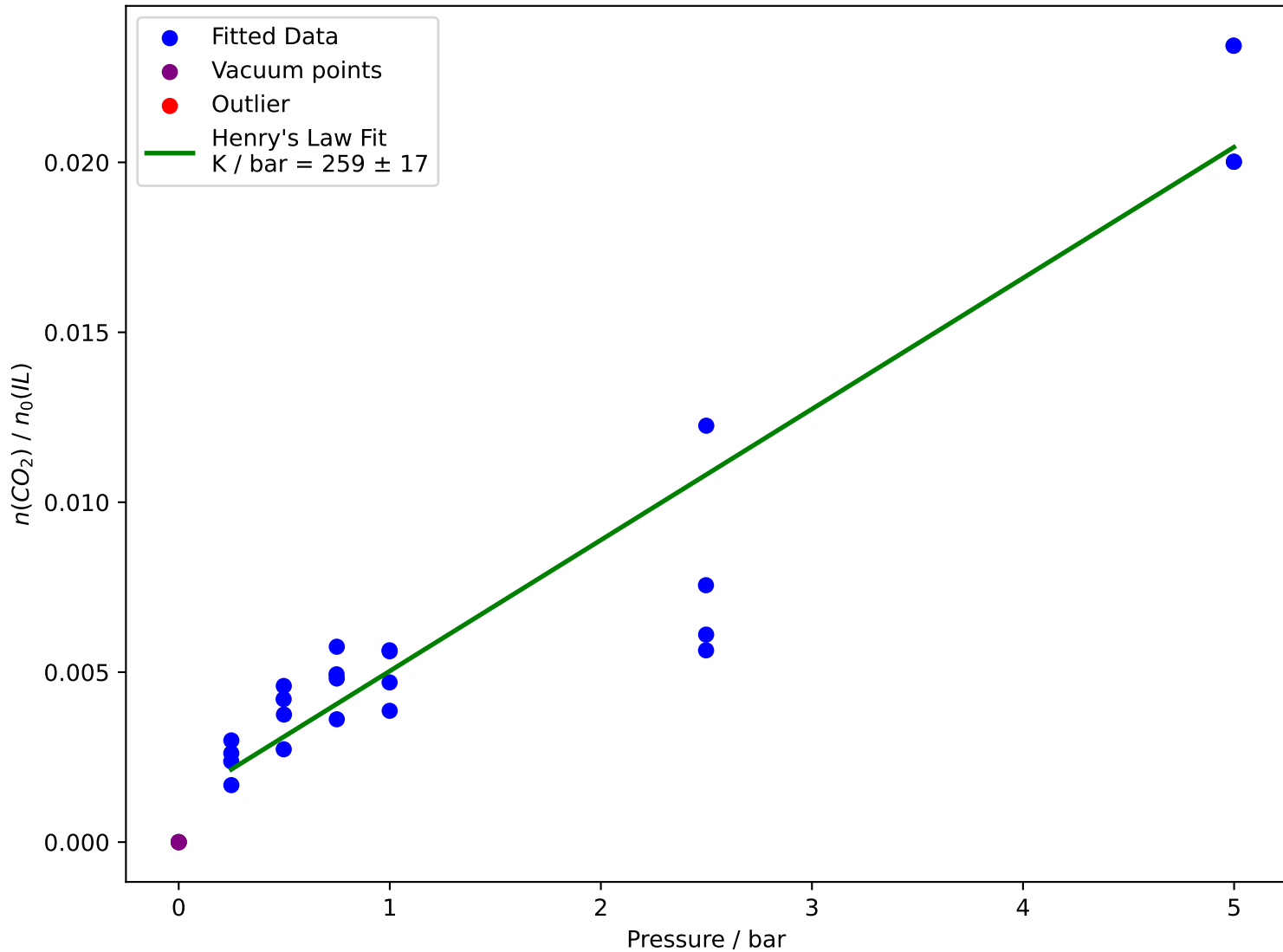

Supplement: Supplementary file 1 — Supplementary Material [file CSSC-18-e202501347-s001.zip › Microbalance_data_analysis/P8881triaz-CH4_2/moleratio_fit_70C.pdf]

# Empty Pan Data at T = 50.18 °C

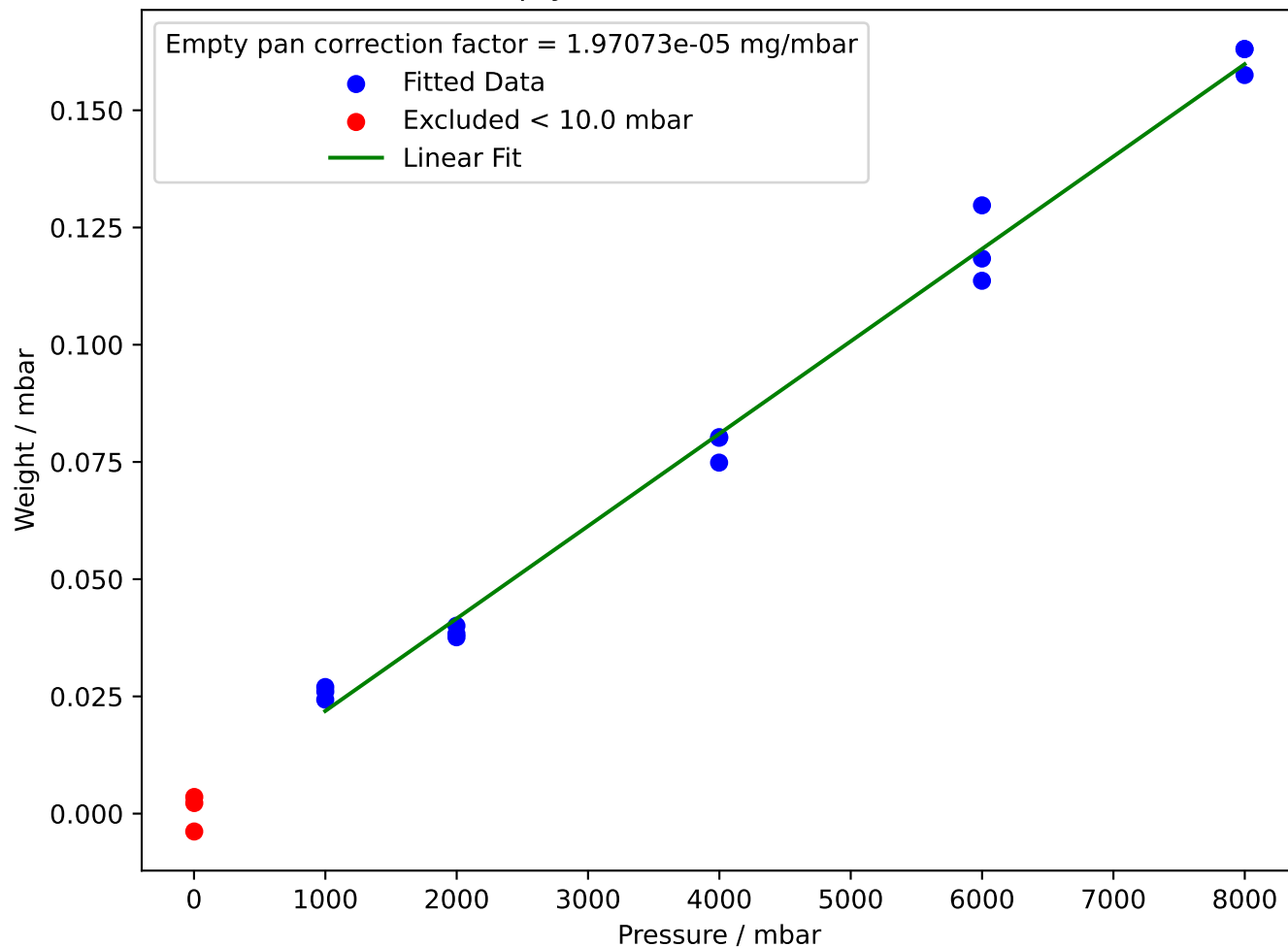

Supplement: Supplementary file 1 — Supplementary Material [file CSSC-18-e202501347-s001.zip › Microbalance_data_analysis/P8881triaz-CH4_3/empty_pan_plot_50.0C.pdf]

# Empty Pan Data at T = 70.12 °C

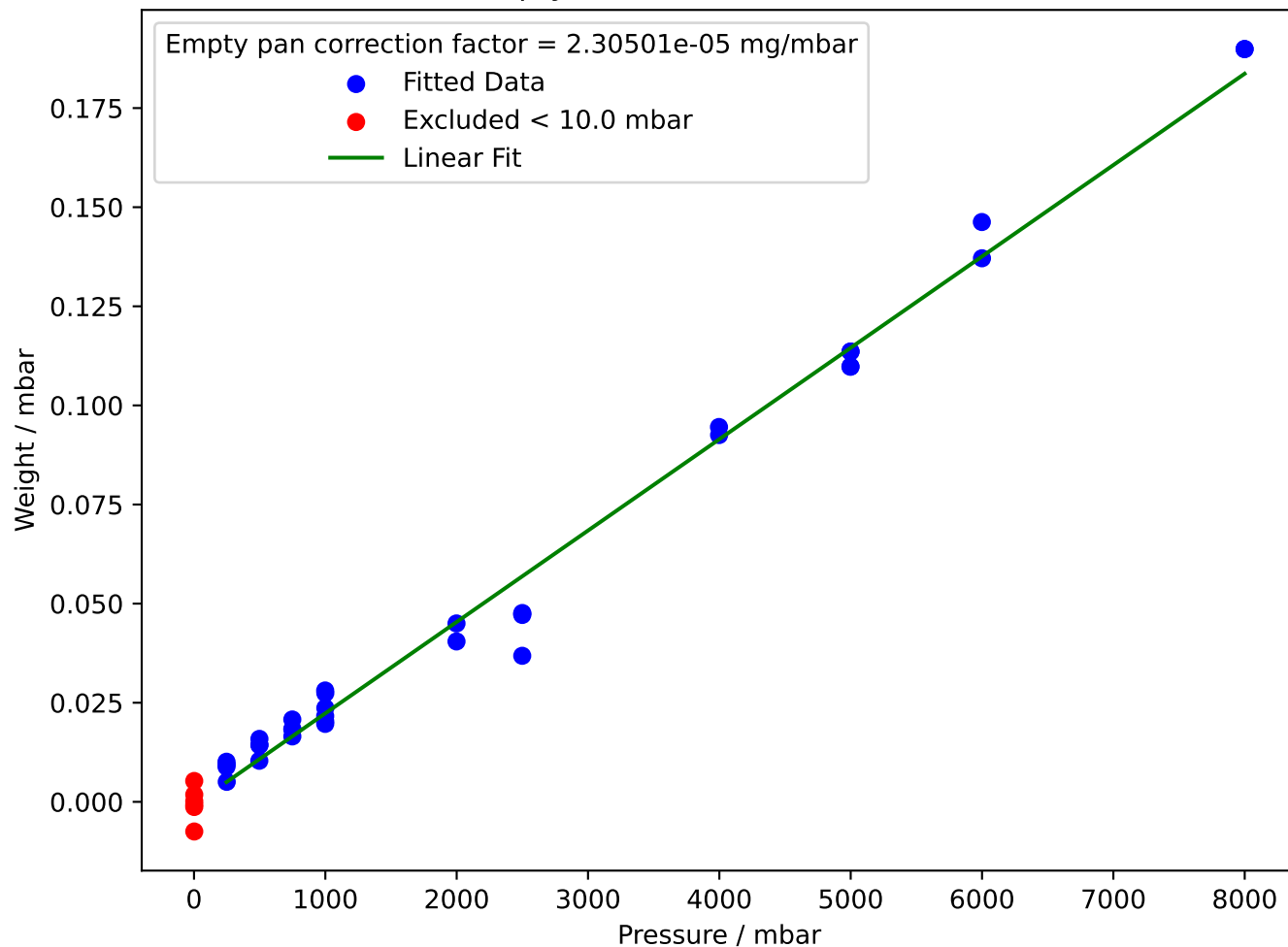

Supplement: Supplementary file 1 — Supplementary Material [file CSSC-18-e202501347-s001.zip › Microbalance_data_analysis/P8881triaz-CH4_3/empty_pan_plot_70.0C.pdf]

# Absorbed gas at T = 50.0°C

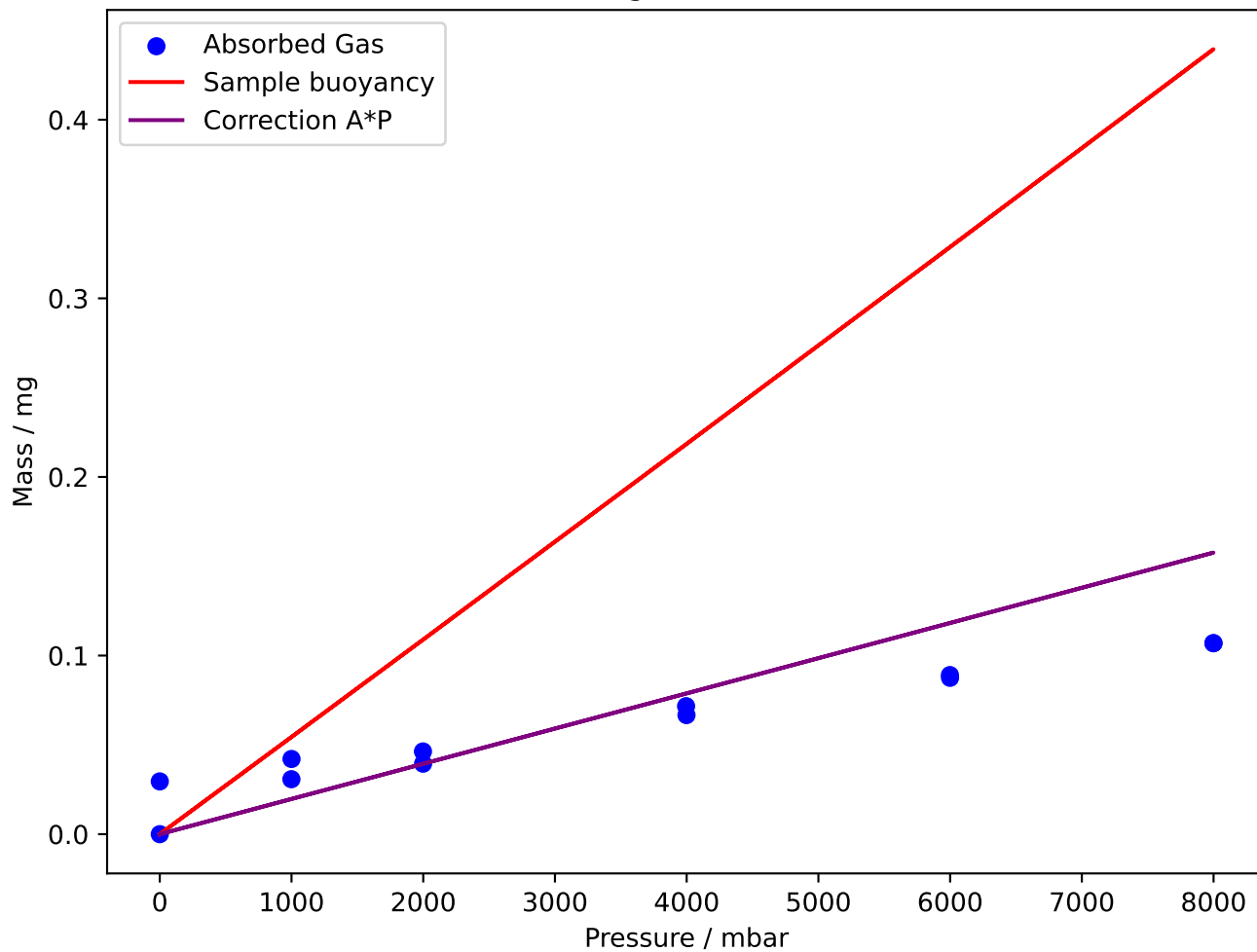

Supplement: Supplementary file 1 — Supplementary Material [file CSSC-18-e202501347-s001.zip › Microbalance_data_analysis/P8881triaz-CH4_3/measurement_plot_50.0C.pdf]

# Absorbed gas at T = 70.0°C

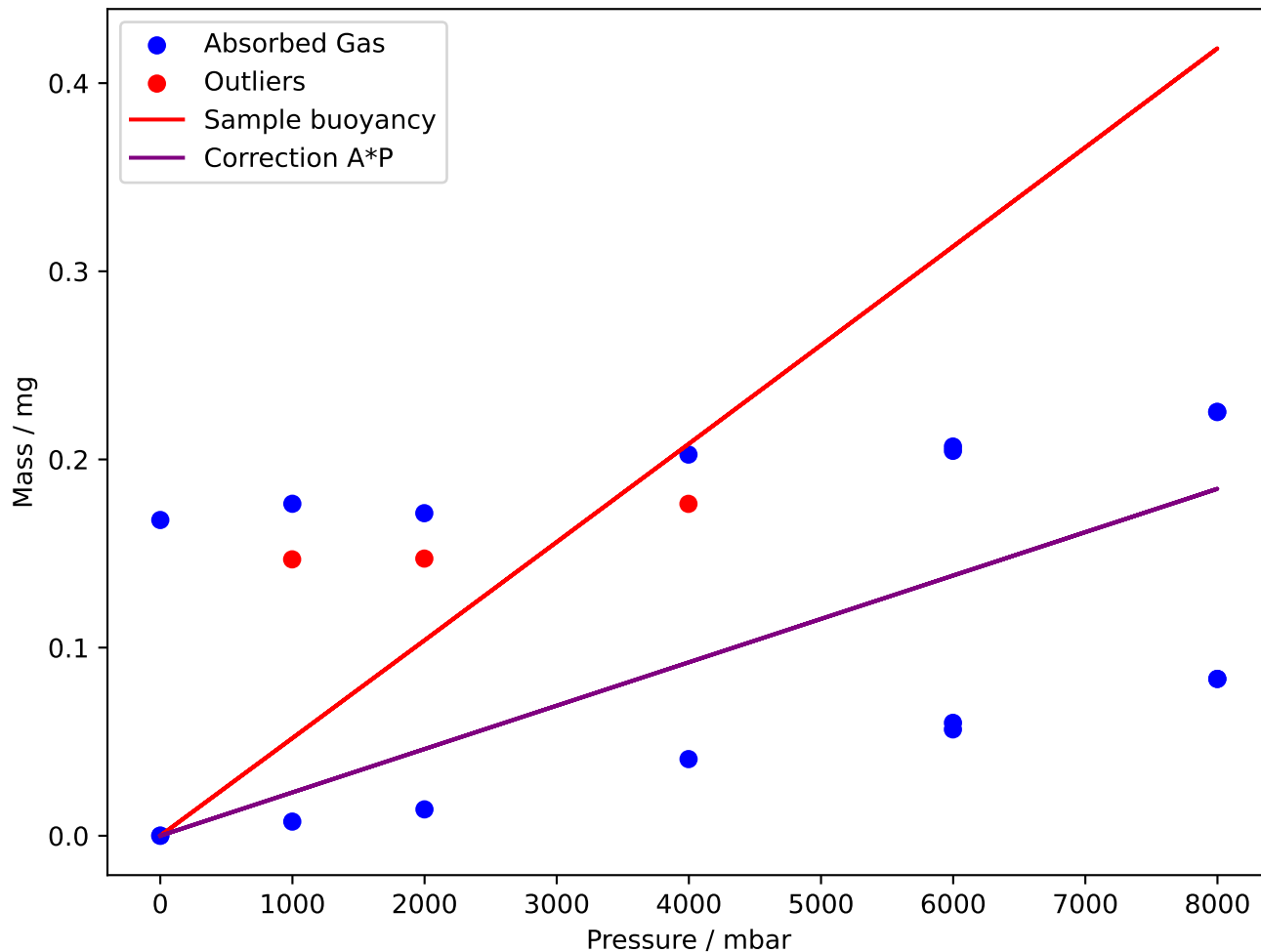

Supplement: Supplementary file 1 — Supplementary Material [file CSSC-18-e202501347-s001.zip › Microbalance_data_analysis/P8881triaz-CH4_3/measurement_plot_70.0C.pdf]

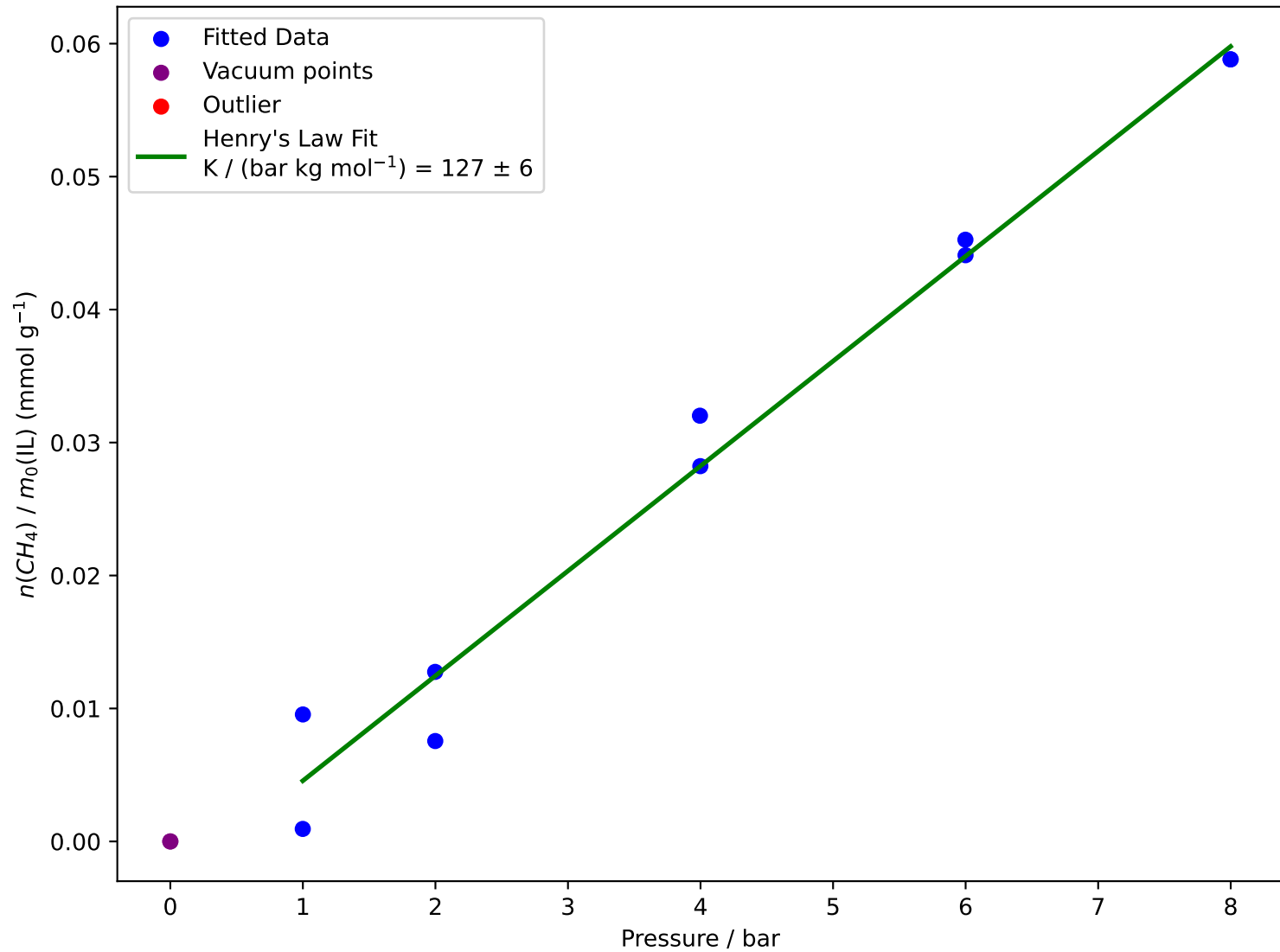

Supplement: Supplementary file 1 — Supplementary Material [file CSSC-18-e202501347-s001.zip › Microbalance_data_analysis/P8881triaz-CH4_3/molality_fit_50C.pdf]

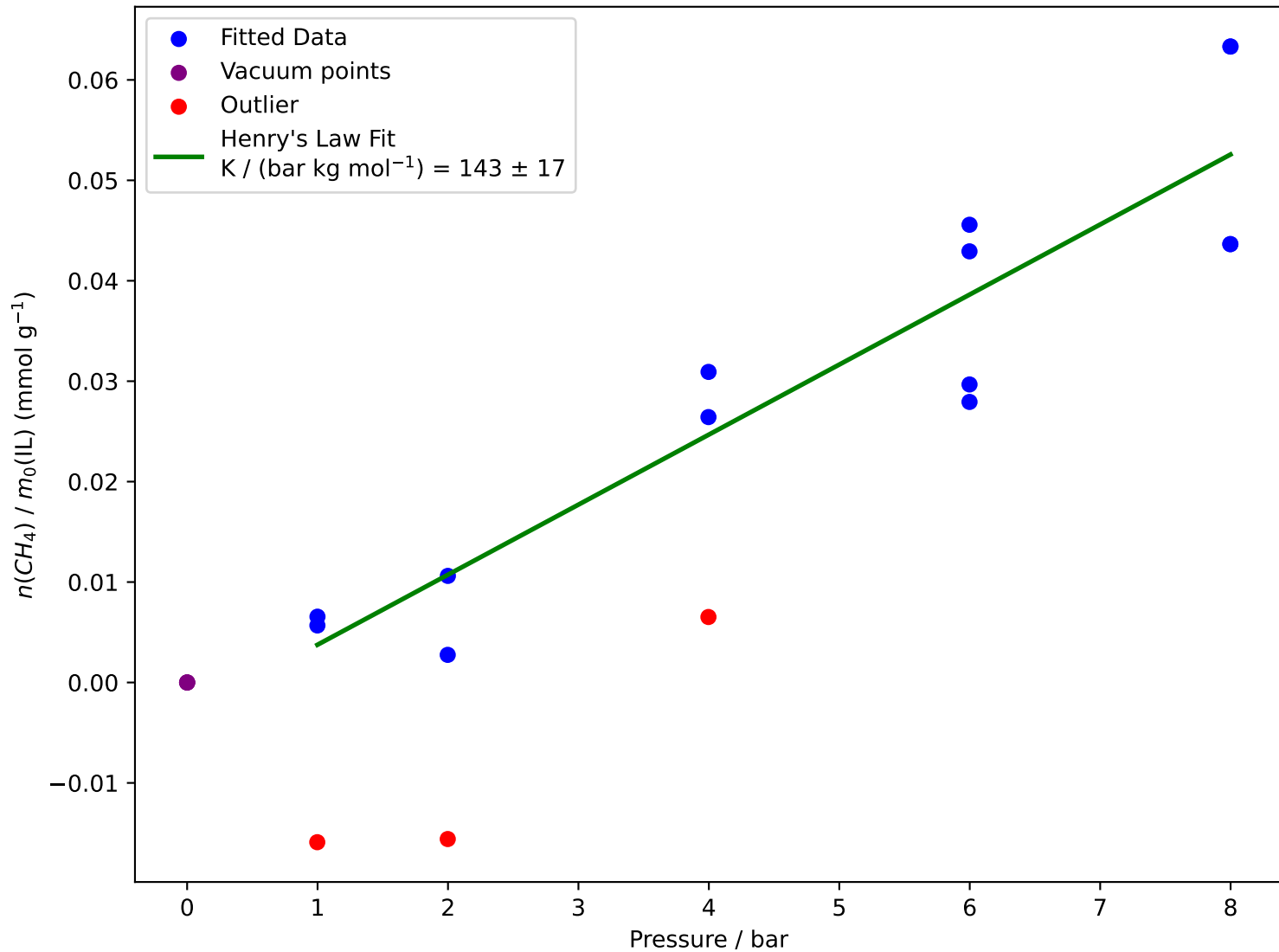

Supplement: Supplementary file 1 — Supplementary Material [file CSSC-18-e202501347-s001.zip › Microbalance_data_analysis/P8881triaz-CH4_3/molality_fit_70C.pdf]

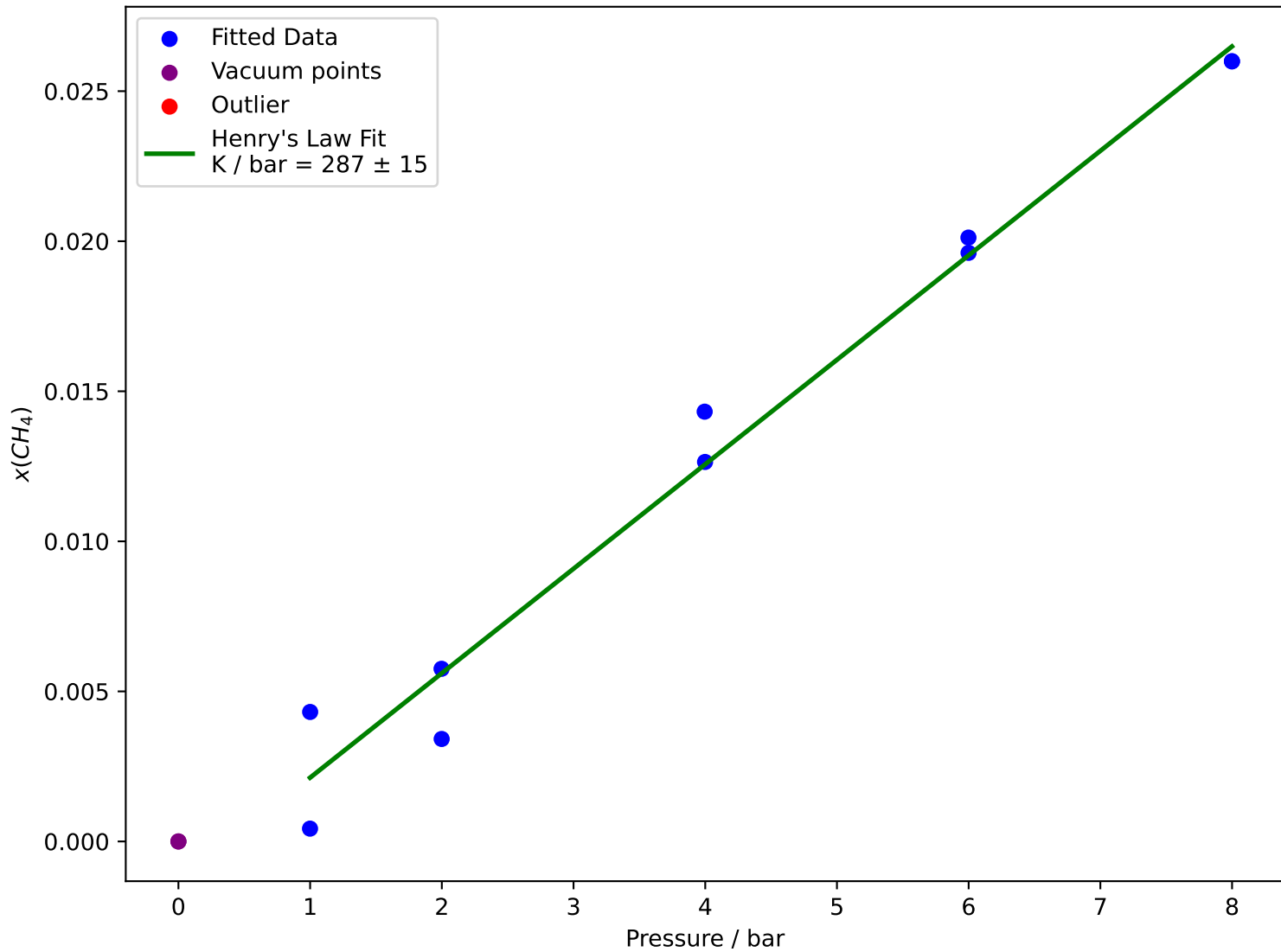

Supplement: Supplementary file 1 — Supplementary Material [file CSSC-18-e202501347-s001.zip › Microbalance_data_analysis/P8881triaz-CH4_3/molefraction_fit_50C.pdf]

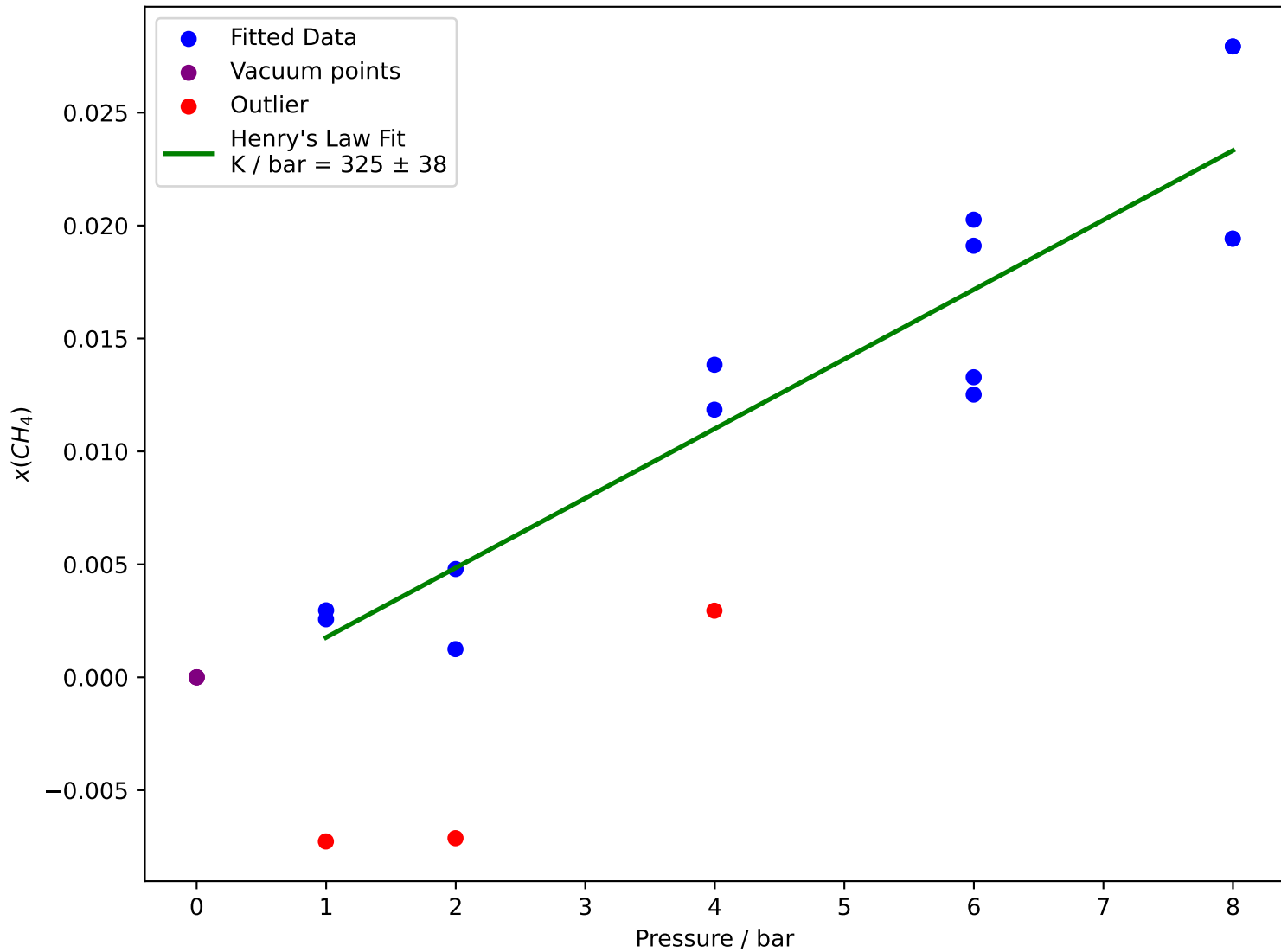

Supplement: Supplementary file 1 — Supplementary Material [file CSSC-18-e202501347-s001.zip › Microbalance_data_analysis/P8881triaz-CH4_3/molefraction_fit_70C.pdf]

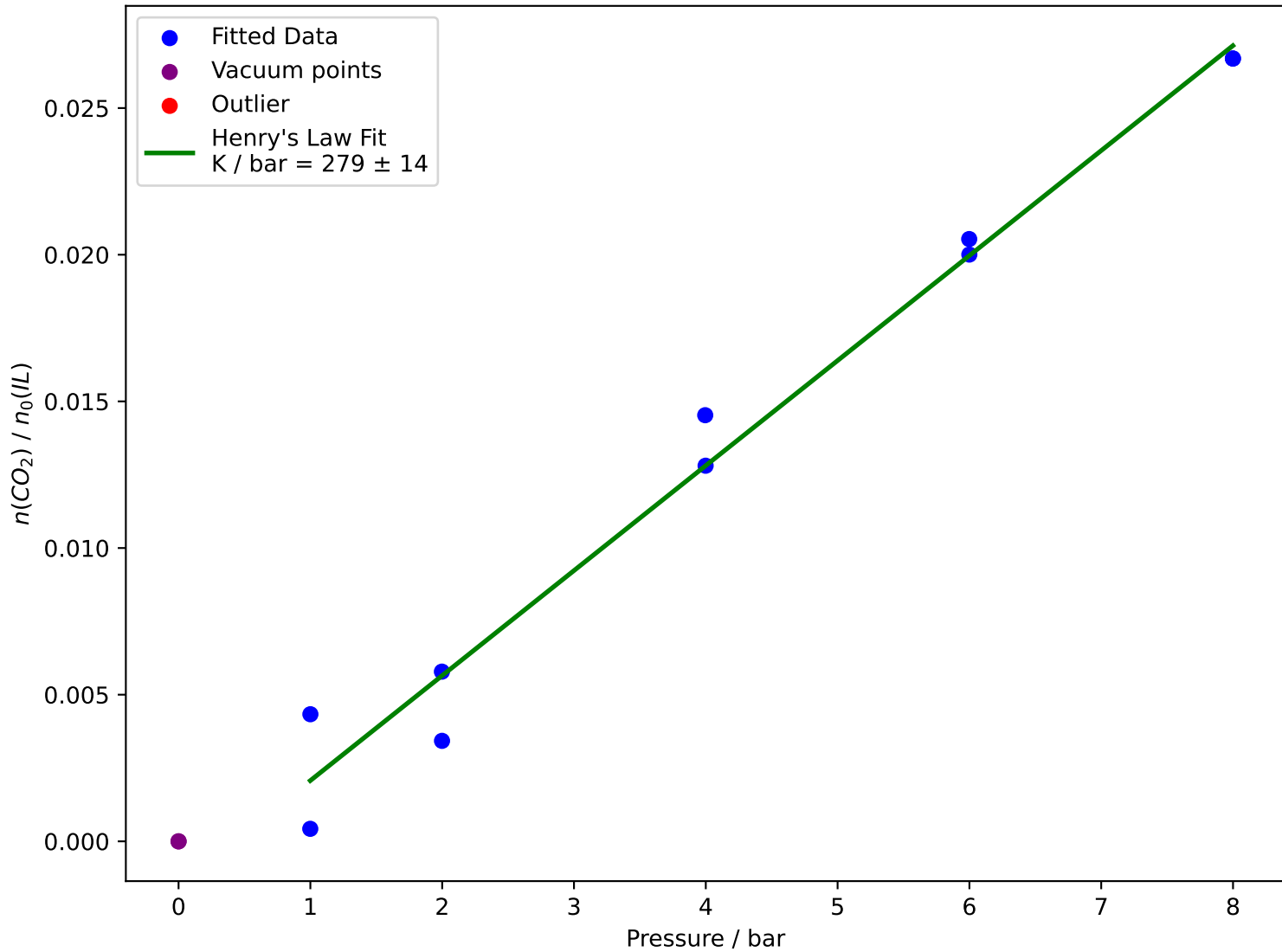

Supplement: Supplementary file 1 — Supplementary Material [file CSSC-18-e202501347-s001.zip › Microbalance_data_analysis/P8881triaz-CH4_3/moleratio_fit_50C.pdf]

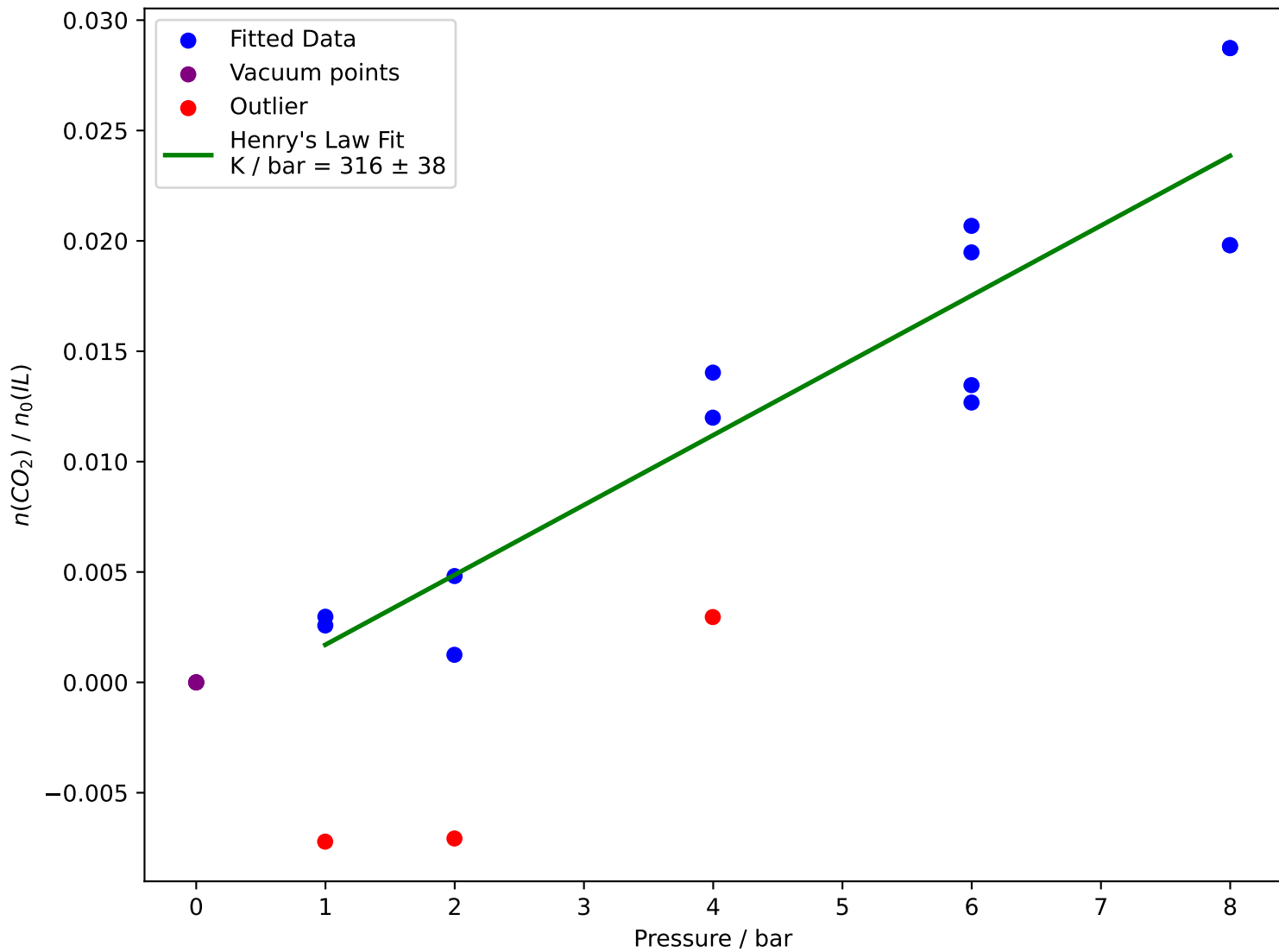

Supplement: Supplementary file 1 — Supplementary Material [file CSSC-18-e202501347-s001.zip › Microbalance_data_analysis/P8881triaz-CH4_3/moleratio_fit_70C.pdf]

# Empty Pan Data at T = 29.98 °C

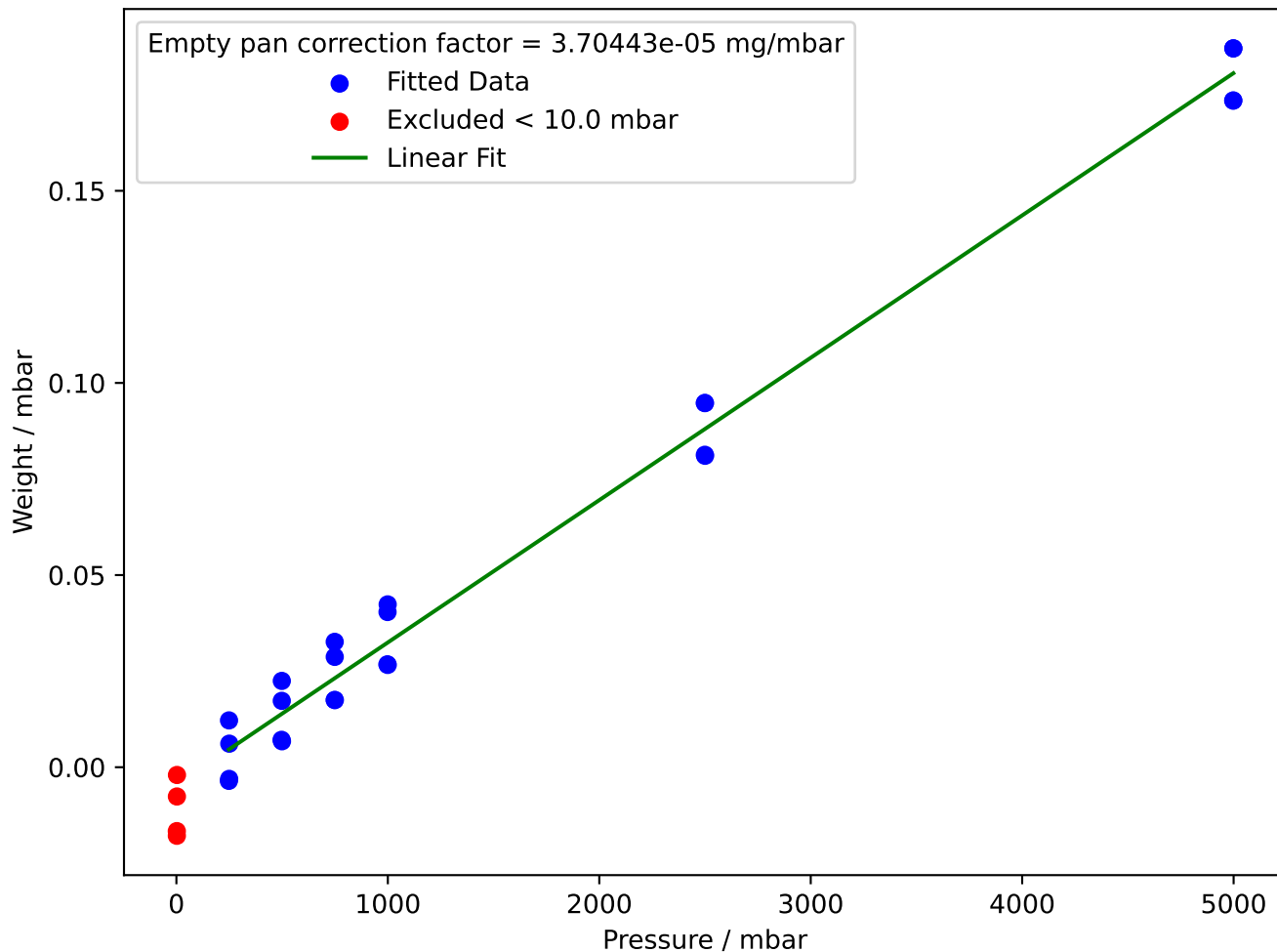

Supplement: Supplementary file 1 — Supplementary Material [file CSSC-18-e202501347-s001.zip › Microbalance_data_analysis/P8881triaz-CO2_1/empty_pan_plot_30.0C.pdf]

# Absorbed gas at T = 30.0°C

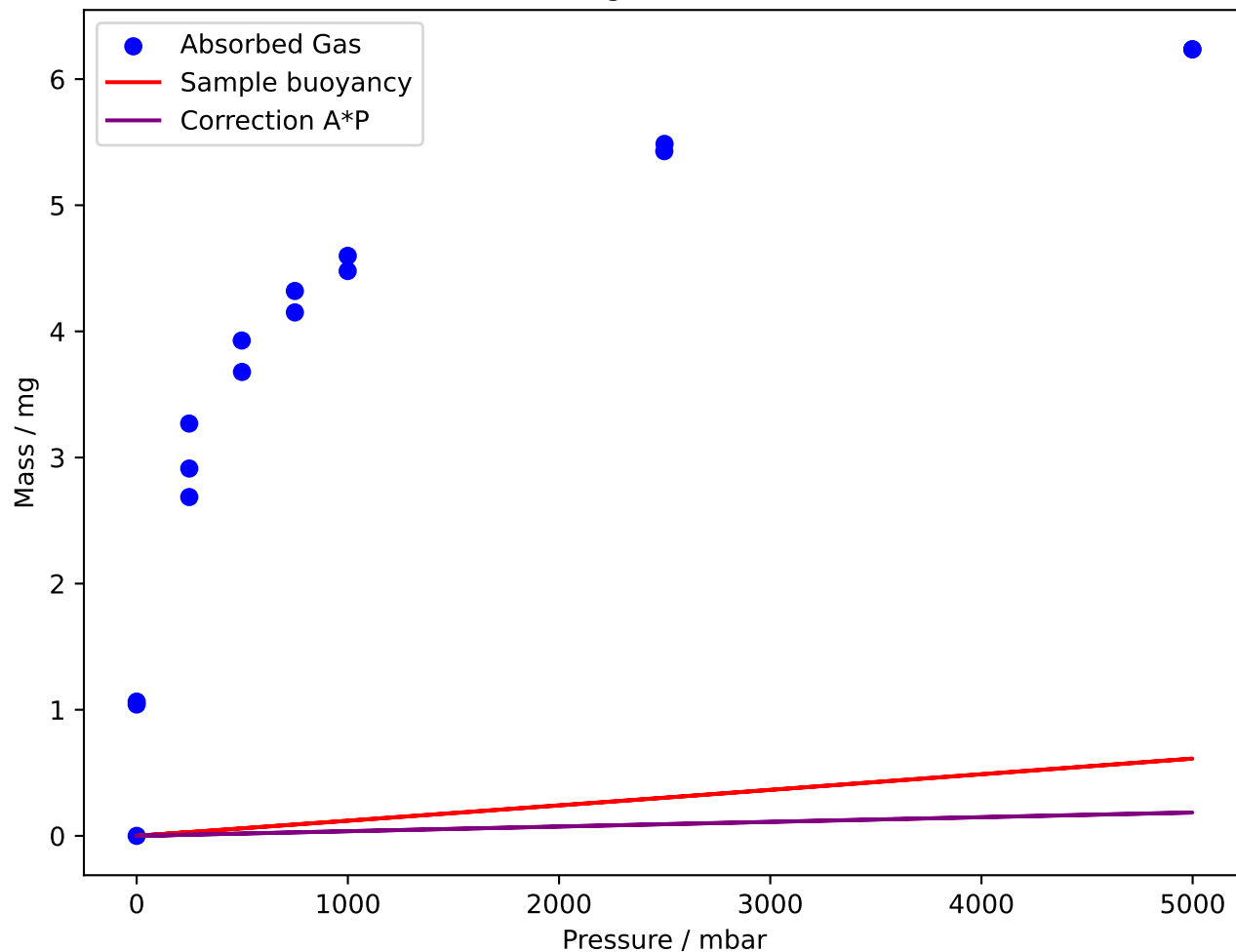

Supplement: Supplementary file 1 — Supplementary Material [file CSSC-18-e202501347-s001.zip › Microbalance_data_analysis/P8881triaz-CO2_1/measurement_plot_30.0C.pdf]

# Empty Pan Data at T = 69.95 °C

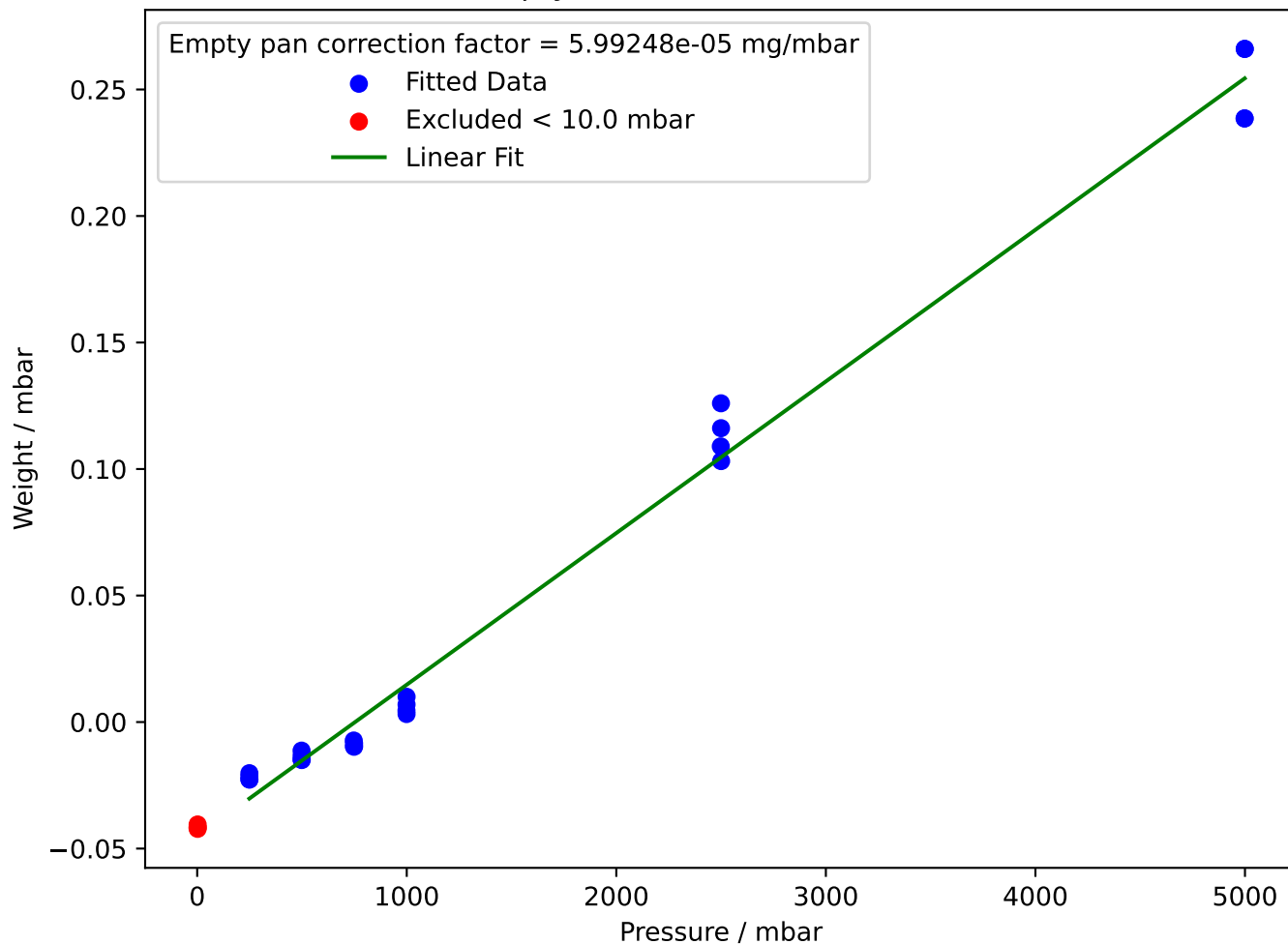

Supplement: Supplementary file 1 — Supplementary Material [file CSSC-18-e202501347-s001.zip › Microbalance_data_analysis/P8881triaz-CO2_2/empty_pan_plot_70.0C.pdf]

# Absorbed gas at T = 70.0°C

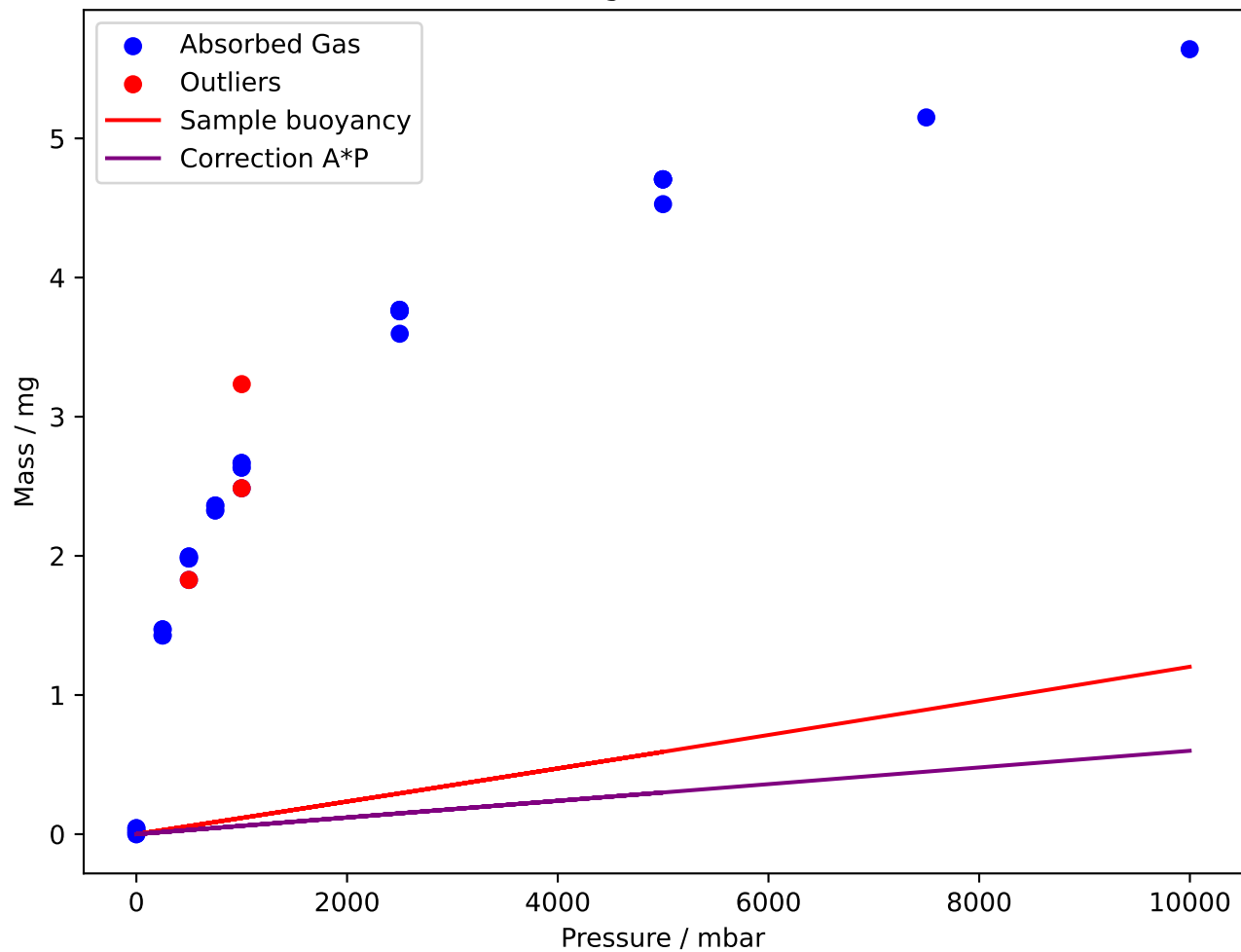

Supplement: Supplementary file 1 — Supplementary Material [file CSSC-18-e202501347-s001.zip › Microbalance_data_analysis/P8881triaz-CO2_2/measurement_plot_70.0C.pdf]

# Empty Pan Data at T = 29.98 °C

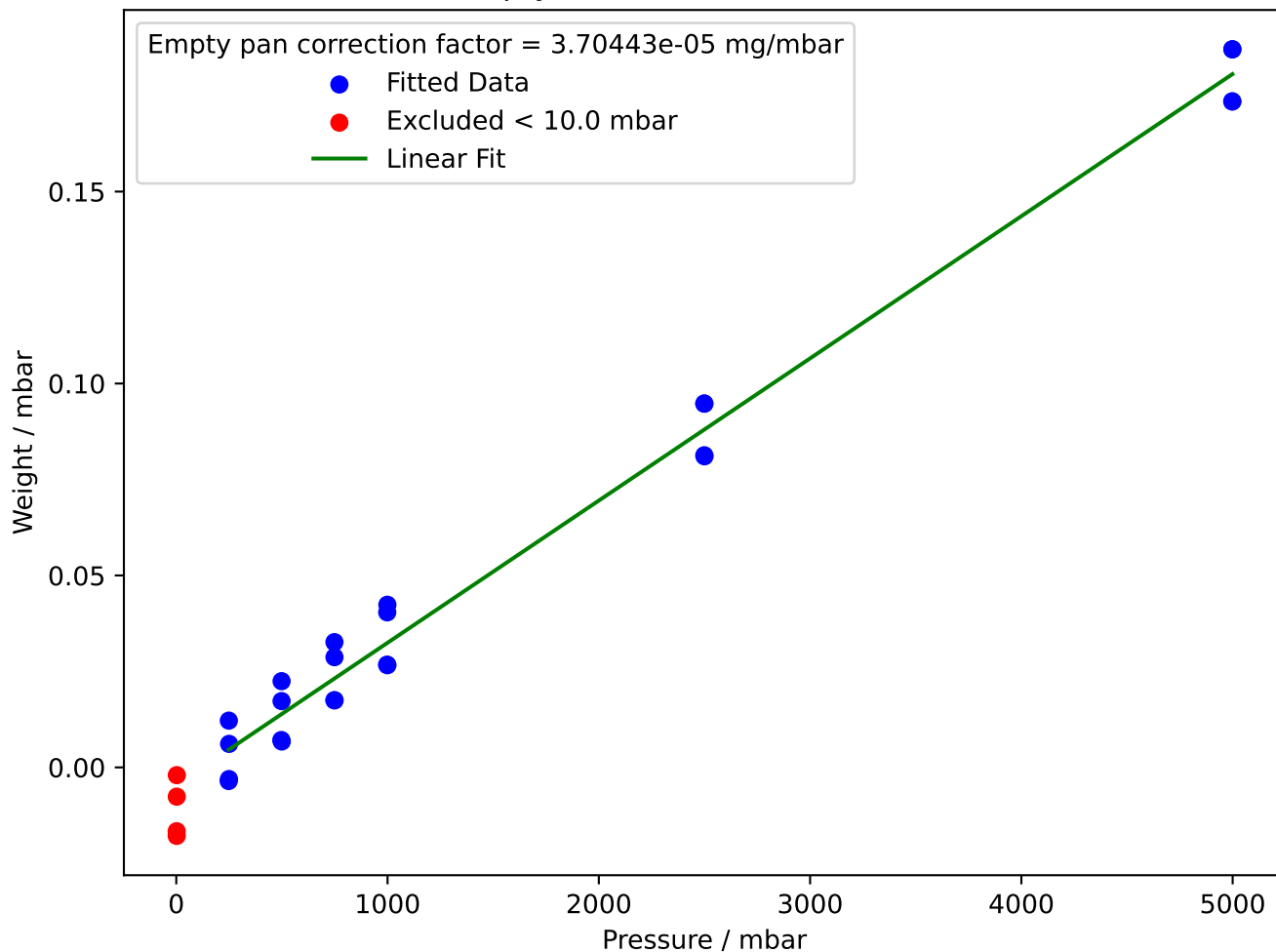

Supplement: Supplementary file 1 — Supplementary Material [file CSSC-18-e202501347-s001.zip › Microbalance_data_analysis/P8881triaz-CO2_3/empty_pan_plot_30.0C.pdf]

Empty Pan Data at T = 50.08 °C

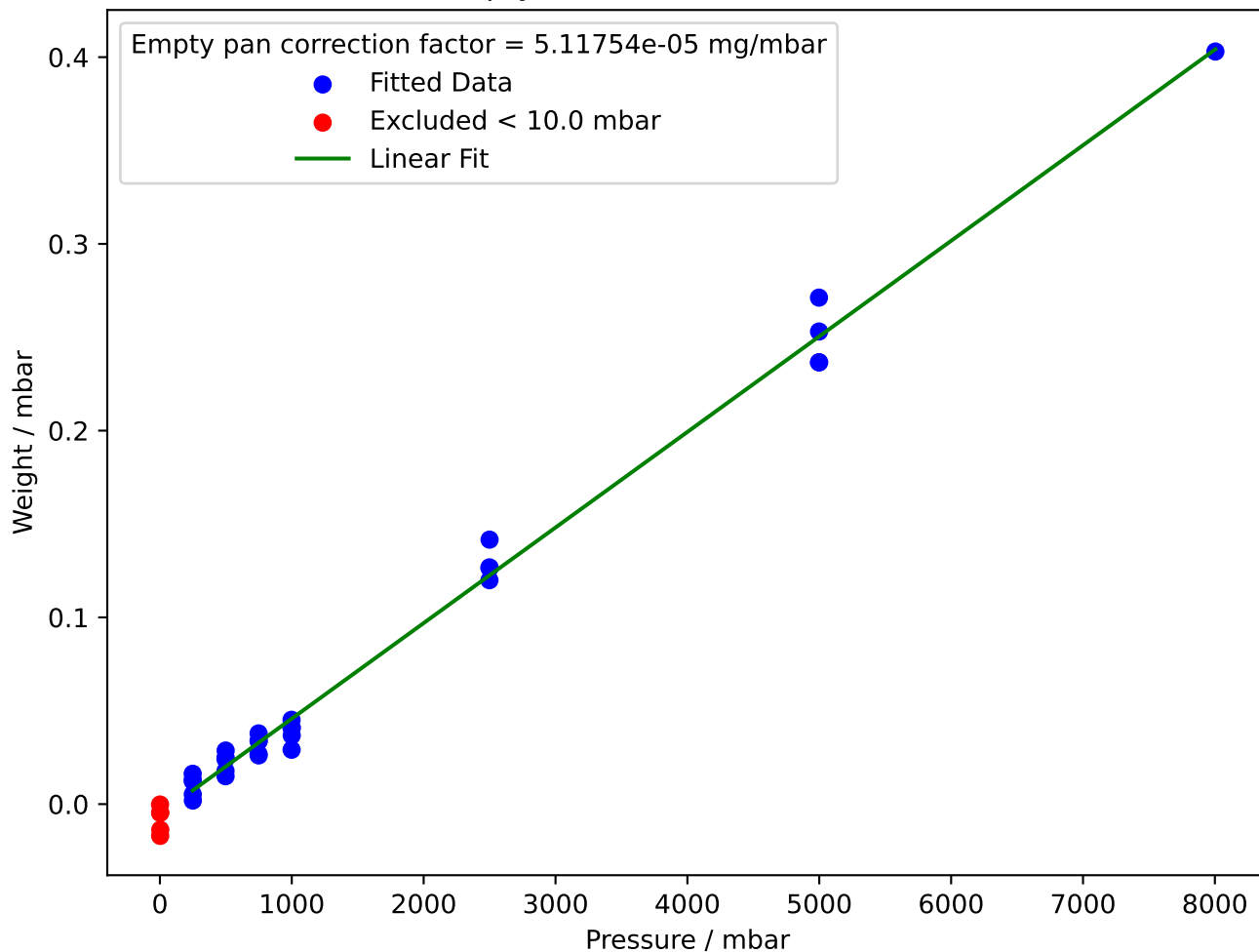

Supplement: Supplementary file 1 — Supplementary Material [file CSSC-18-e202501347-s001.zip › Microbalance_data_analysis/P8881triaz-CO2_3/empty_pan_plot_50.0C.pdf]

# Absorbed gas at T = 30.0°C

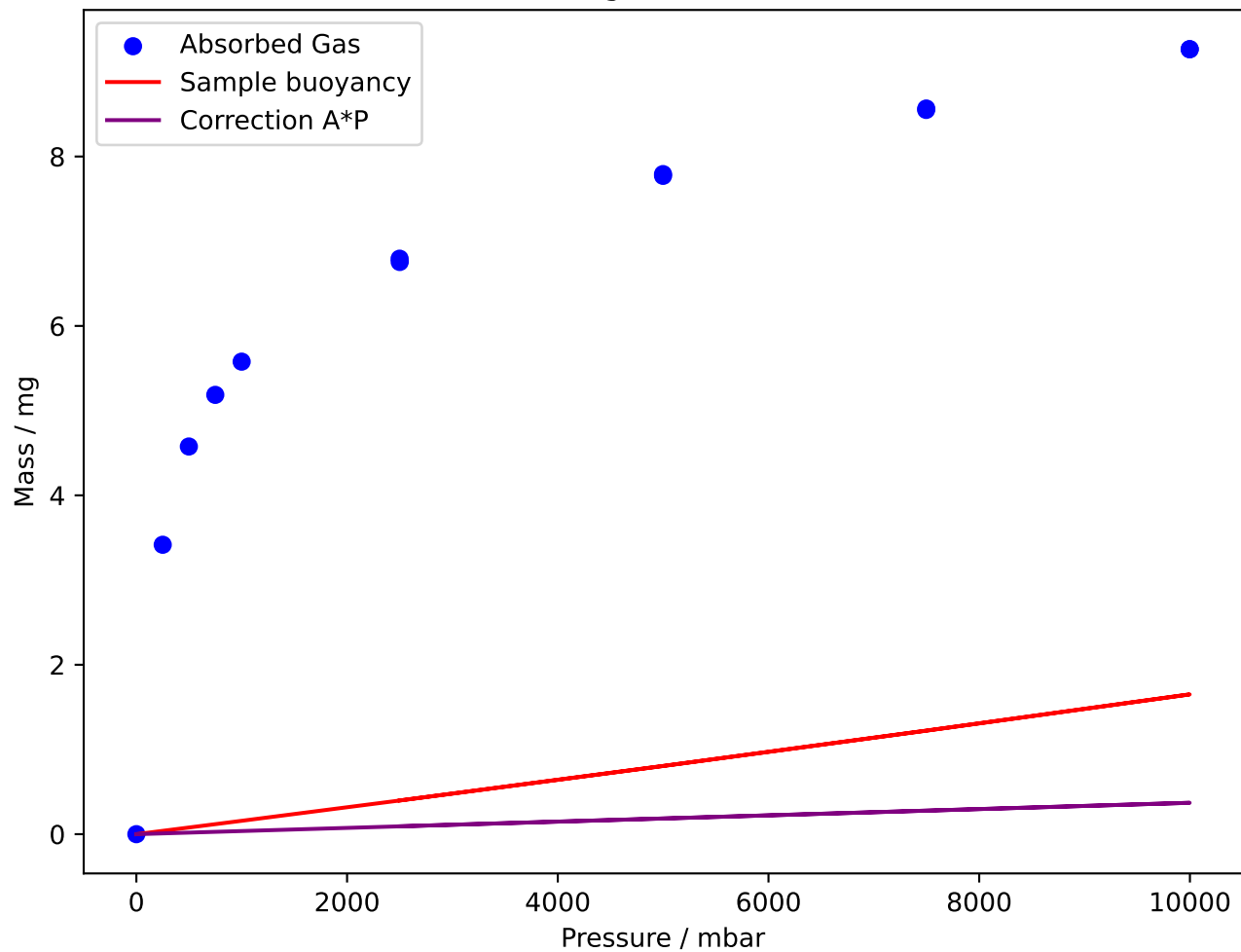

Supplement: Supplementary file 1 — Supplementary Material [file CSSC-18-e202501347-s001.zip › Microbalance_data_analysis/P8881triaz-CO2_3/measurement_plot_30.0C.pdf]

# Absorbed gas at T = 50.0°C

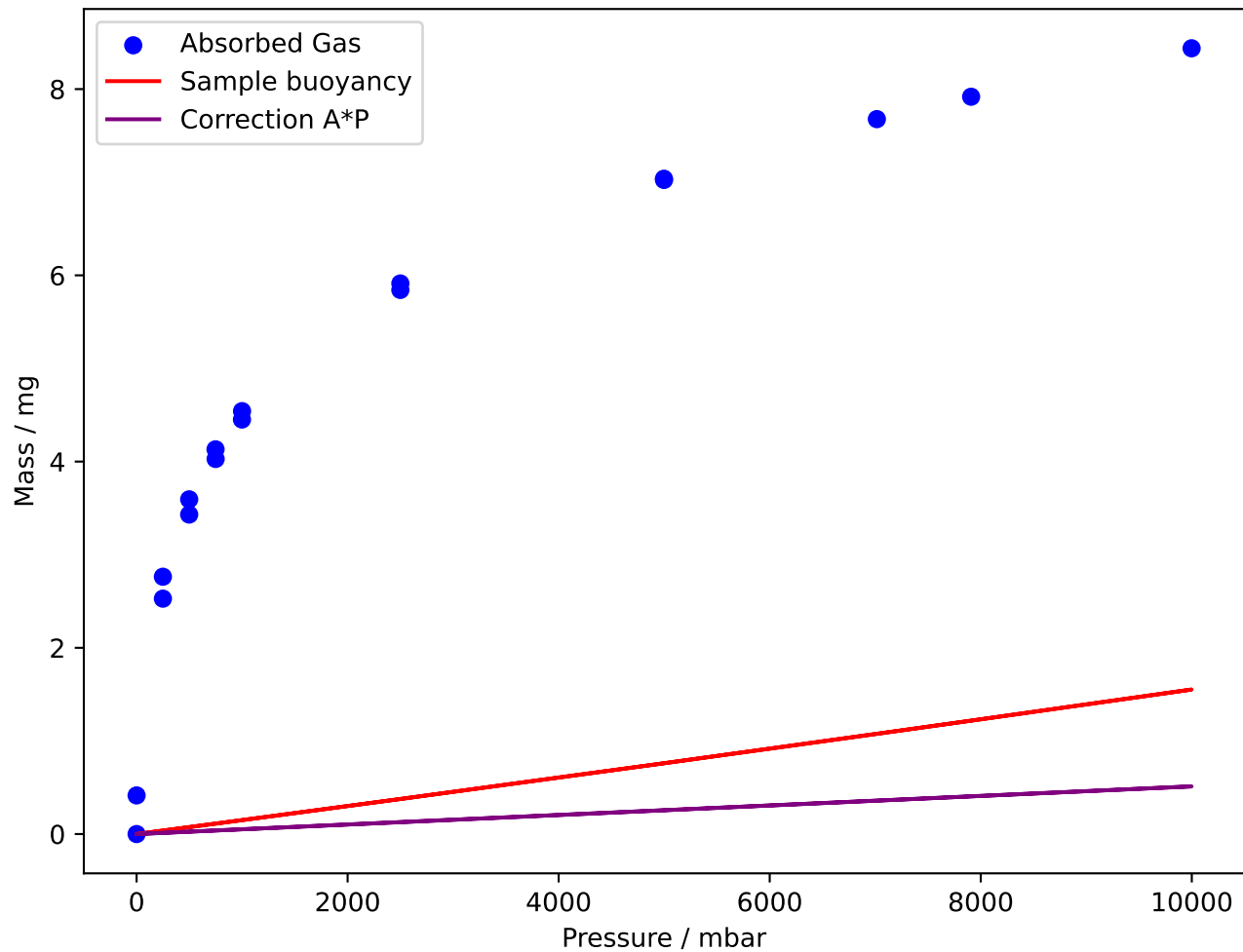

Supplement: Supplementary file 1 — Supplementary Material [file CSSC-18-e202501347-s001.zip › Microbalance_data_analysis/P8881triaz-CO2_3/measurement_plot_50.0C.pdf]

# Empty Pan Data at T = 30.00 °C

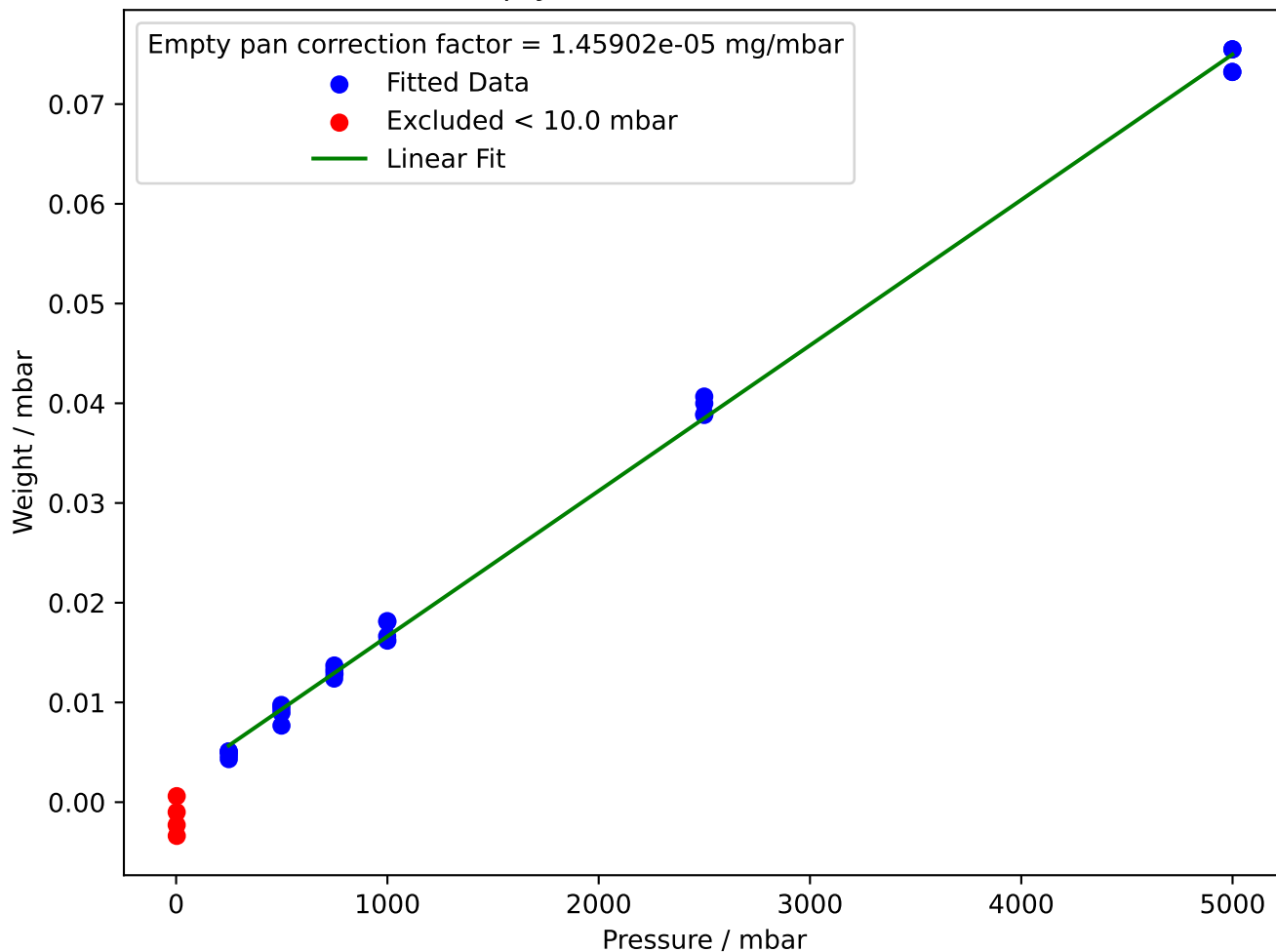

Supplement: Supplementary file 1 — Supplementary Material [file CSSC-18-e202501347-s001.zip › Microbalance_data_analysis/ZIF8-CH4/empty_pan_plot_30.0C.pdf]

# Empty Pan Data at T = 50.18 °C

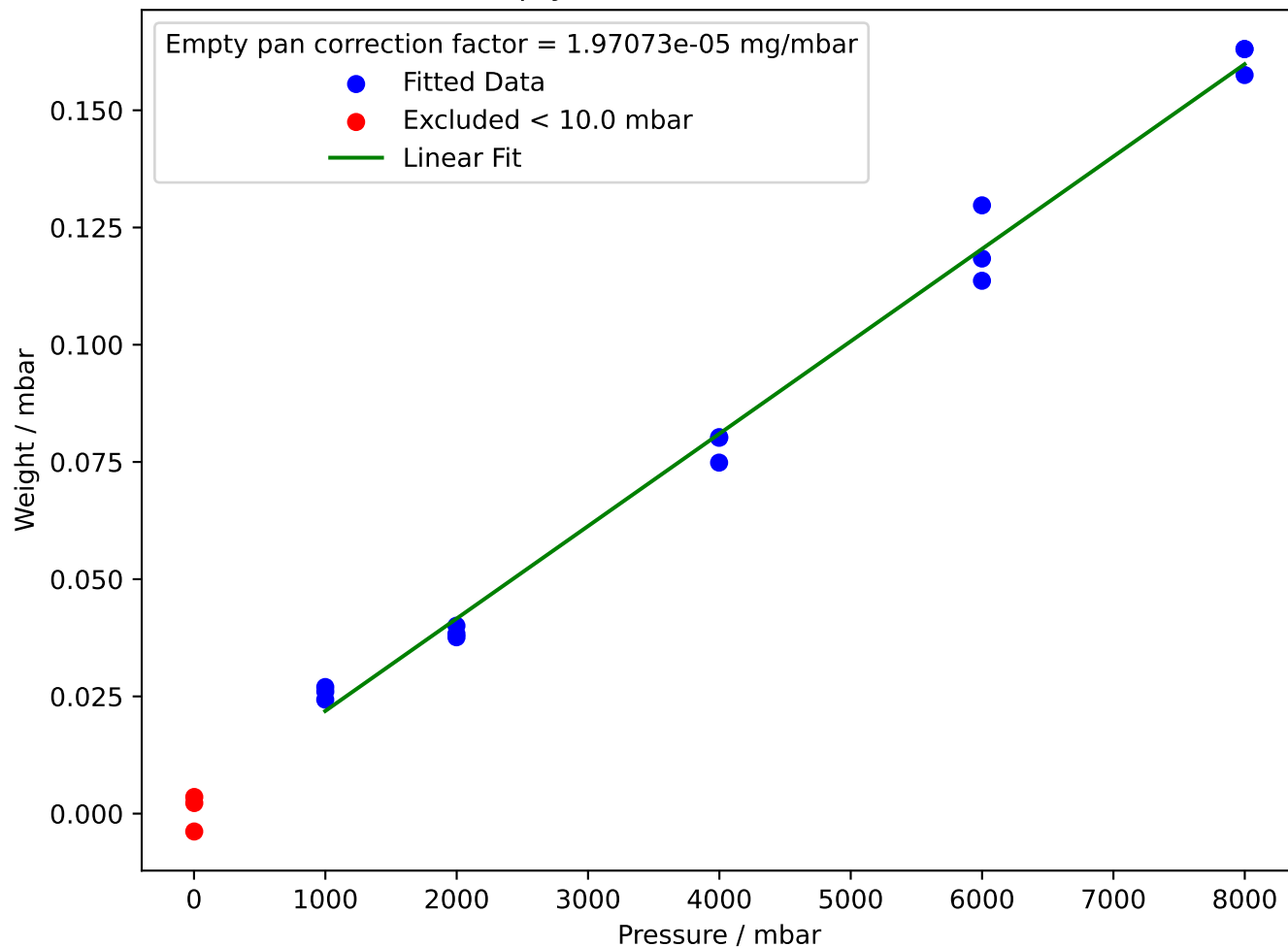

Supplement: Supplementary file 1 — Supplementary Material [file CSSC-18-e202501347-s001.zip › Microbalance_data_analysis/ZIF8-CH4/empty_pan_plot_50.0C.pdf]

# Empty Pan Data at T = 70.12 °C

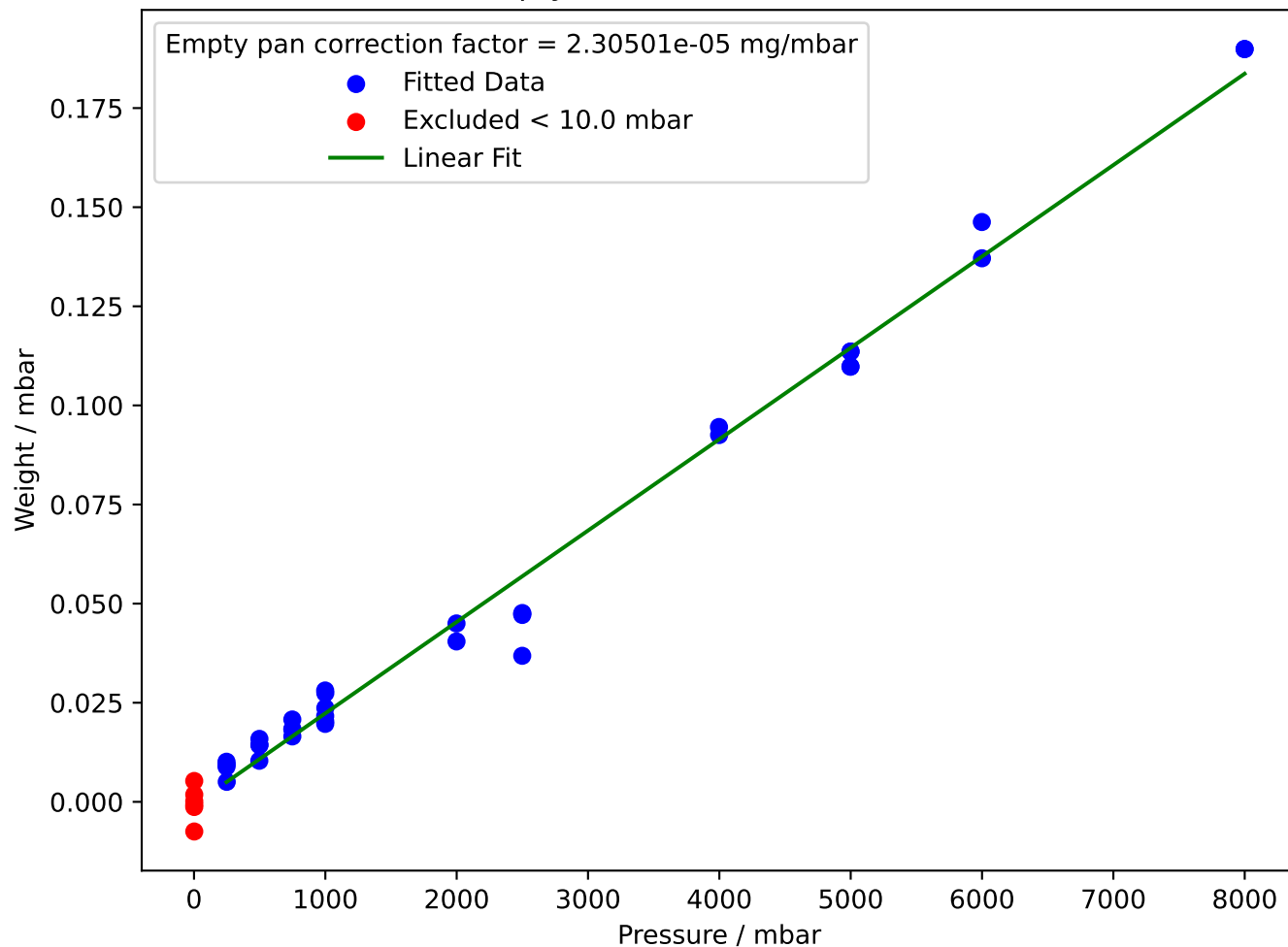

Supplement: Supplementary file 1 — Supplementary Material [file CSSC-18-e202501347-s001.zip › Microbalance_data_analysis/ZIF8-CH4/empty_pan_plot_70.0C.pdf]

Absorbed gas at  $T = 30.0^{\circ}\text{C}$

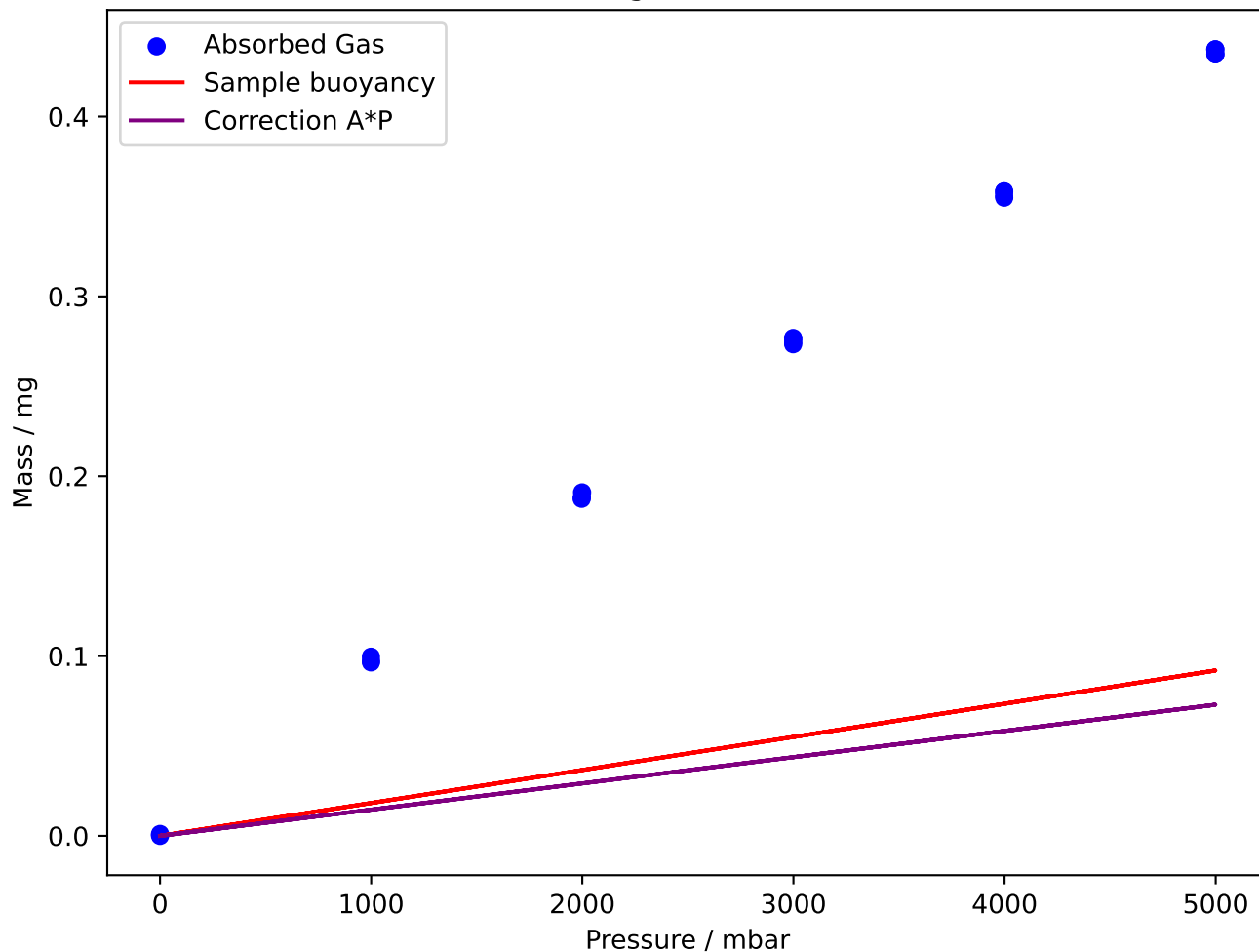

Supplement: Supplementary file 1 — Supplementary Material [file CSSC-18-e202501347-s001.zip › Microbalance_data_analysis/ZIF8-CH4/measurement_plot_30.0C.pdf]

# Absorbed gas at $T = 50.0^{\circ}\text{C}$

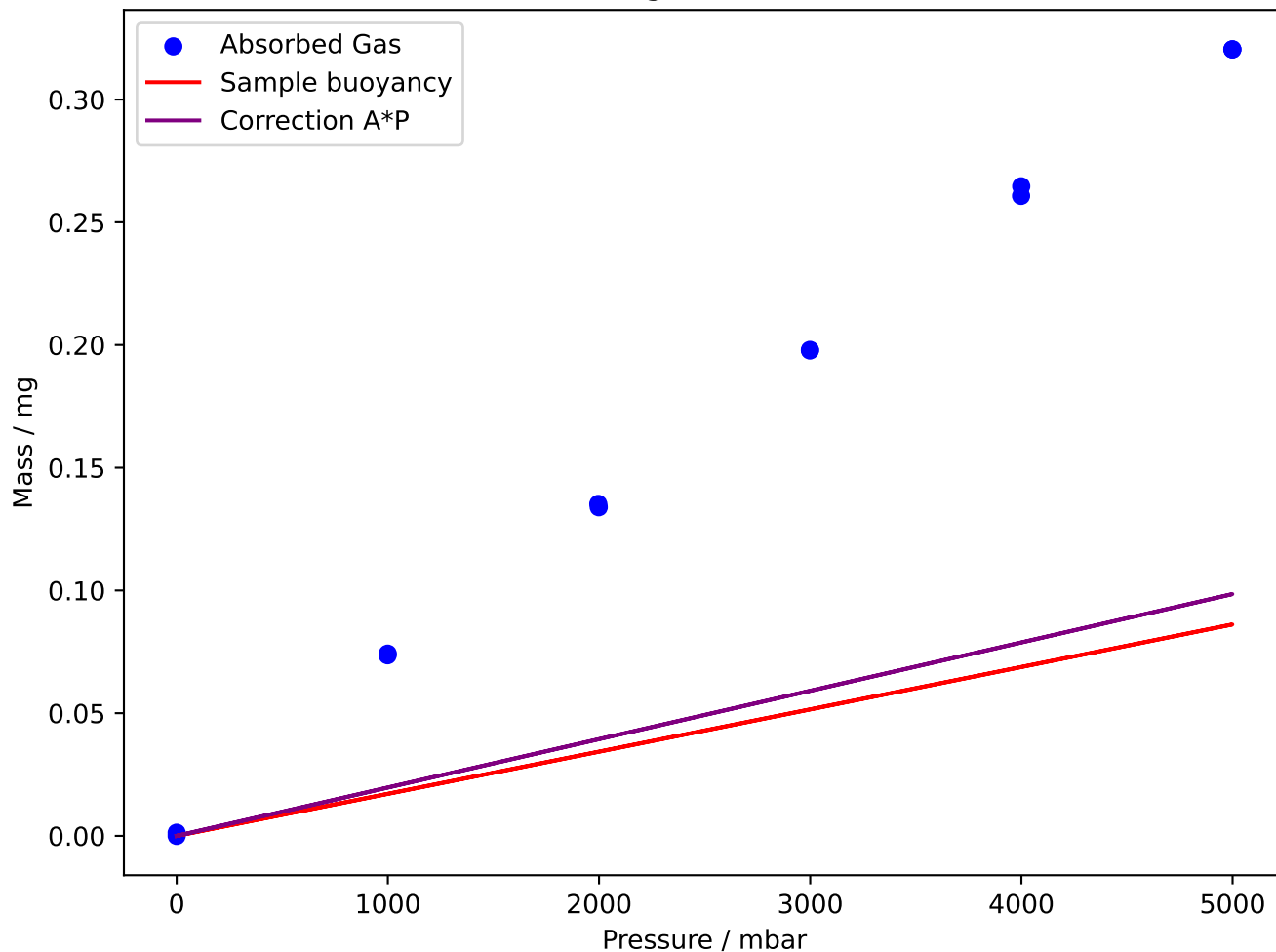

Supplement: Supplementary file 1 — Supplementary Material [file CSSC-18-e202501347-s001.zip › Microbalance_data_analysis/ZIF8-CH4/measurement_plot_50.0C.pdf]

Absorbed gas at  $T = 70.0^{\circ}\text{C}$

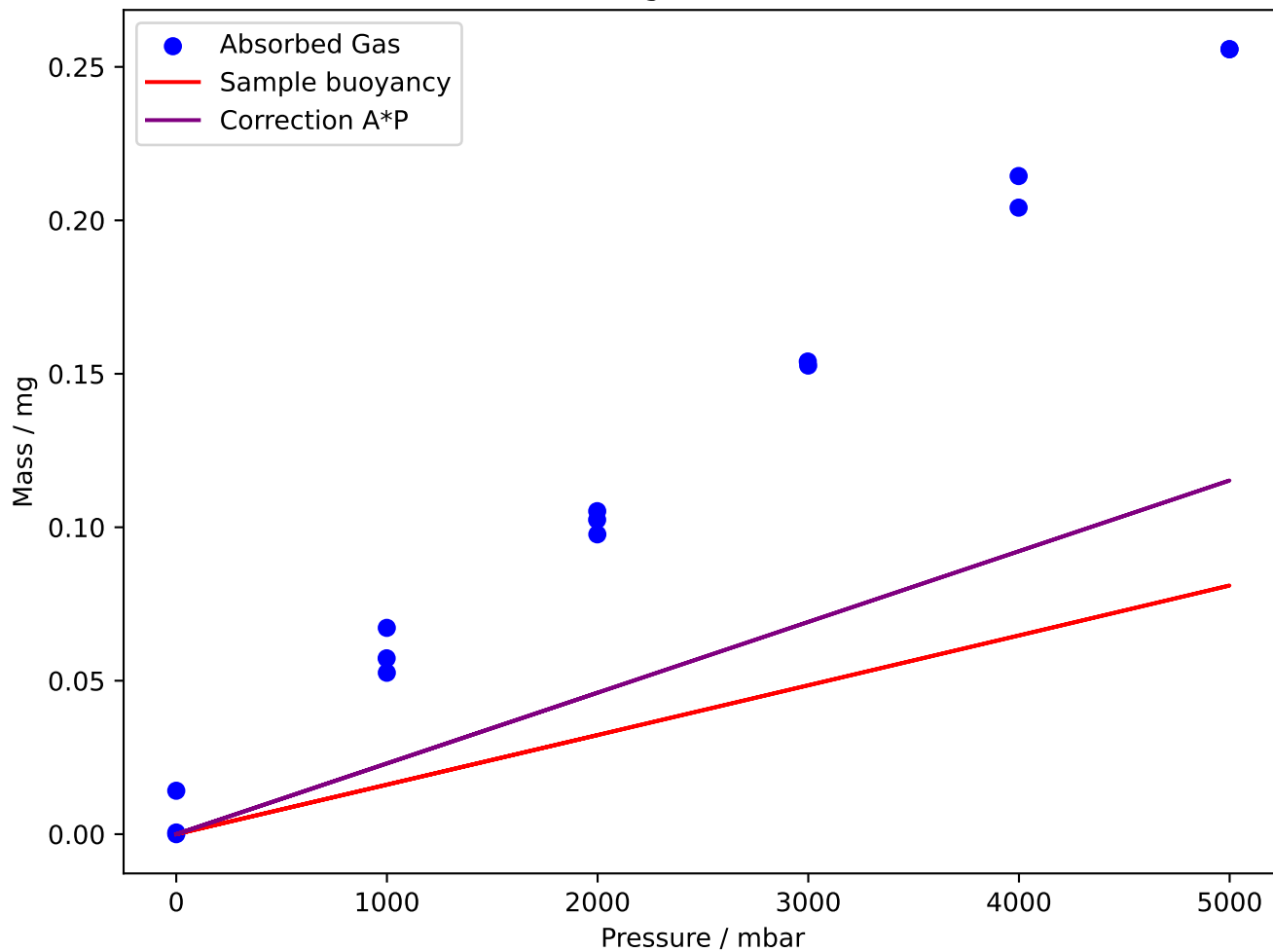

Supplement: Supplementary file 1 — Supplementary Material [file CSSC-18-e202501347-s001.zip › Microbalance_data_analysis/ZIF8-CH4/measurement_plot_70.0C.pdf]

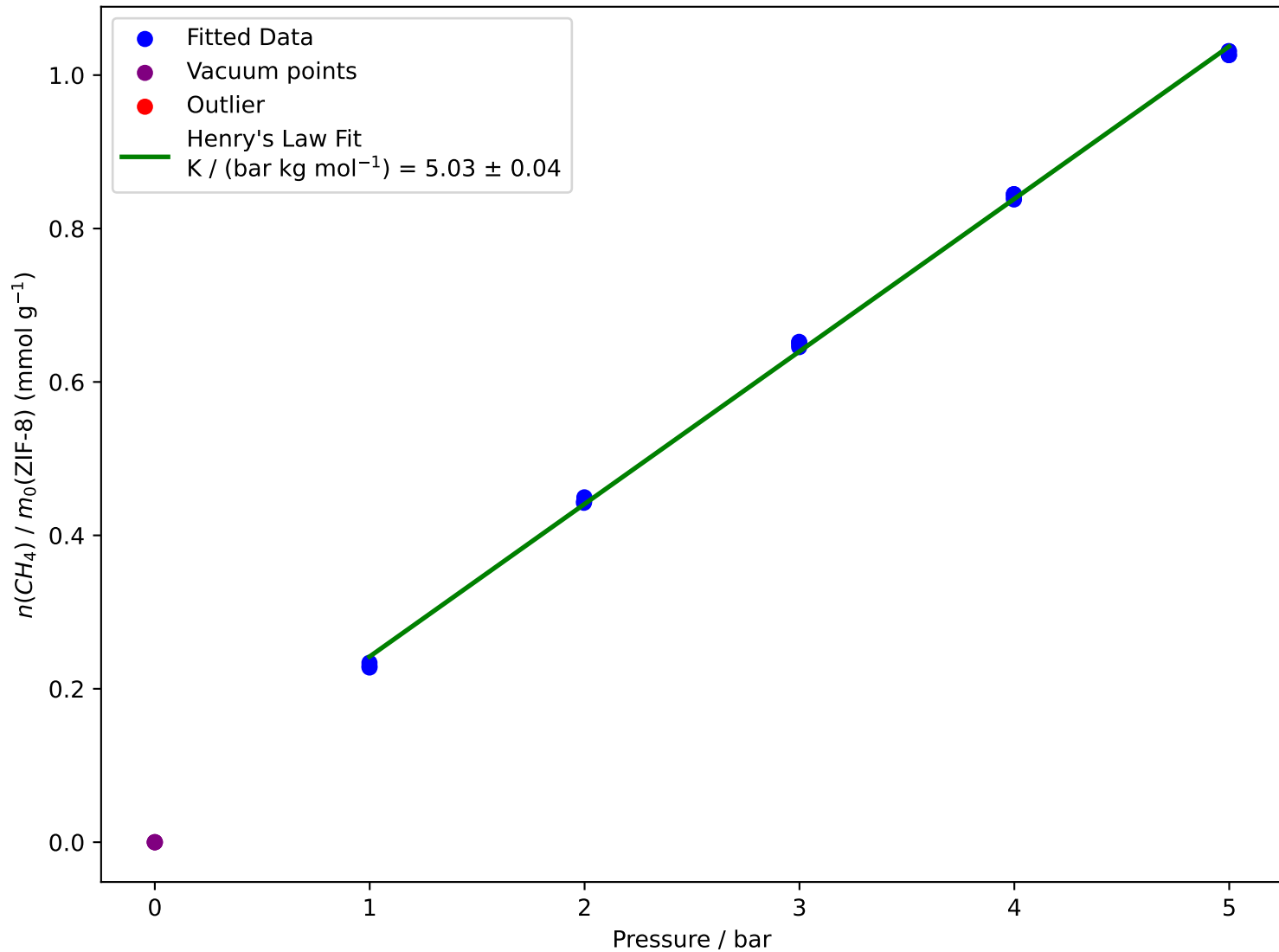

Supplement: Supplementary file 1 — Supplementary Material [file CSSC-18-e202501347-s001.zip › Microbalance_data_analysis/ZIF8-CH4/molality_fit_30C.pdf]

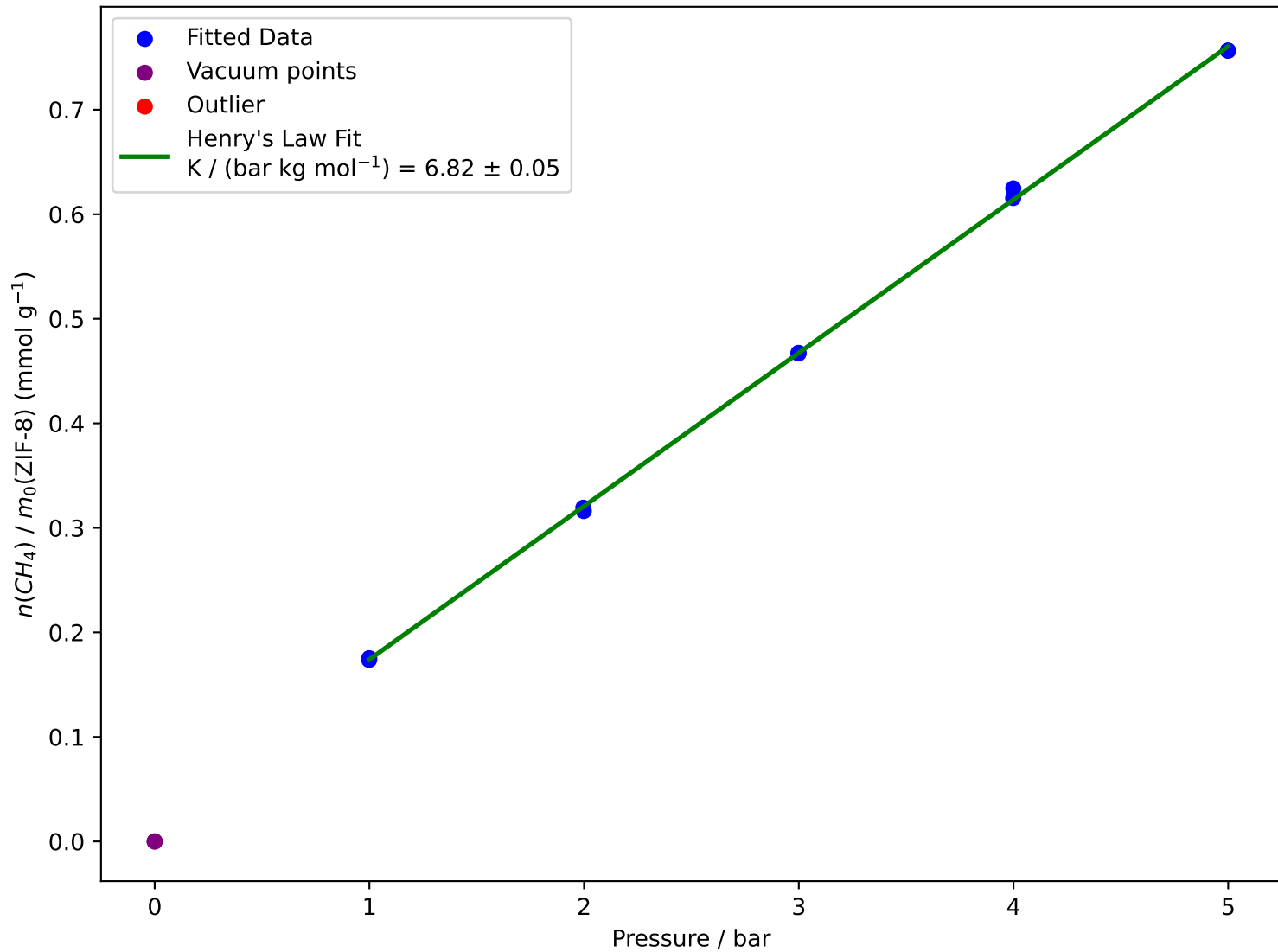

Supplement: Supplementary file 1 — Supplementary Material [file CSSC-18-e202501347-s001.zip › Microbalance_data_analysis/ZIF8-CH4/molality_fit_50C.pdf]

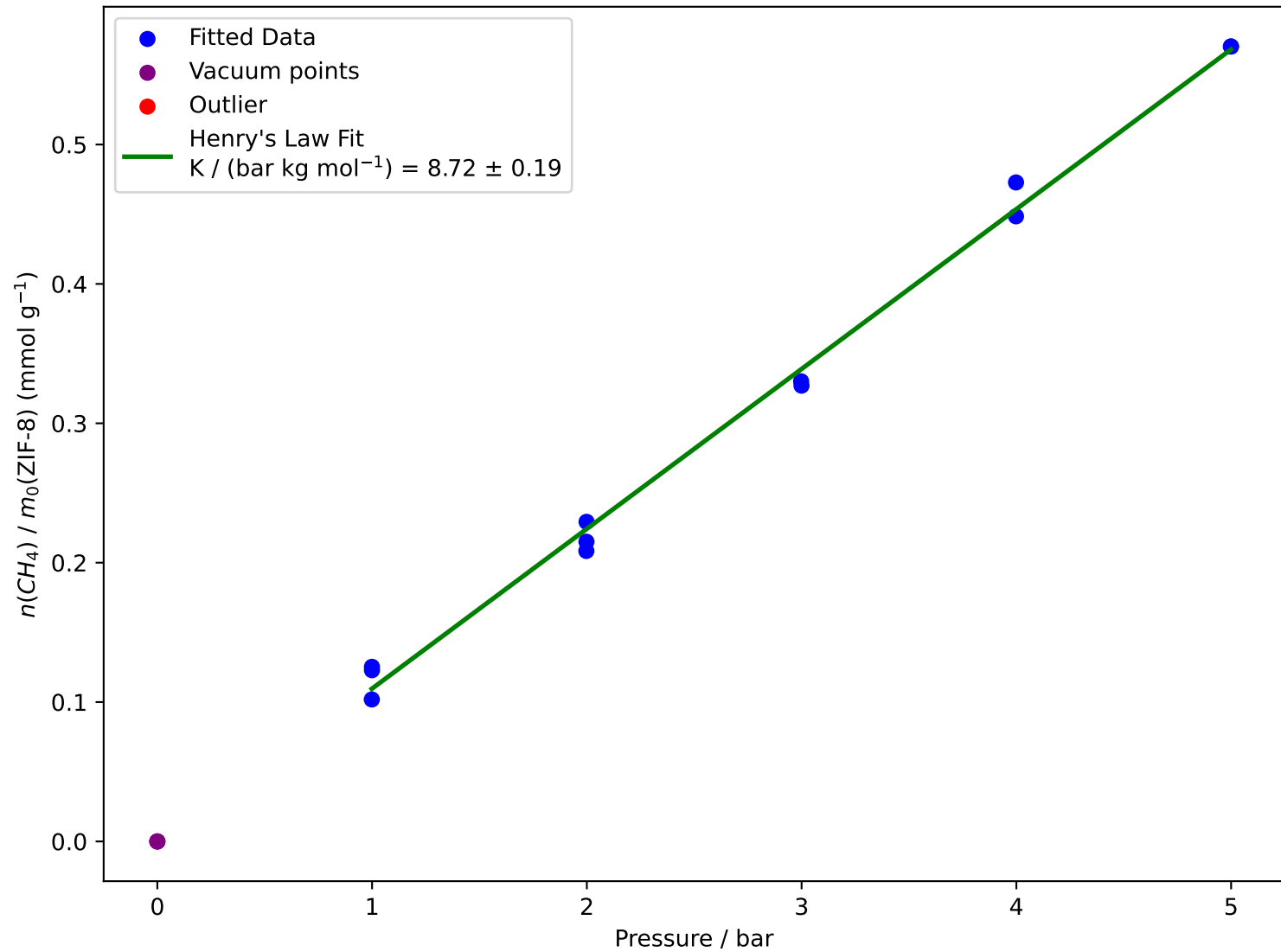

Supplement: Supplementary file 1 — Supplementary Material [file CSSC-18-e202501347-s001.zip › Microbalance_data_analysis/ZIF8-CH4/molality_fit_70C.pdf]

# Empty Pan Data at T = 29.98 °C

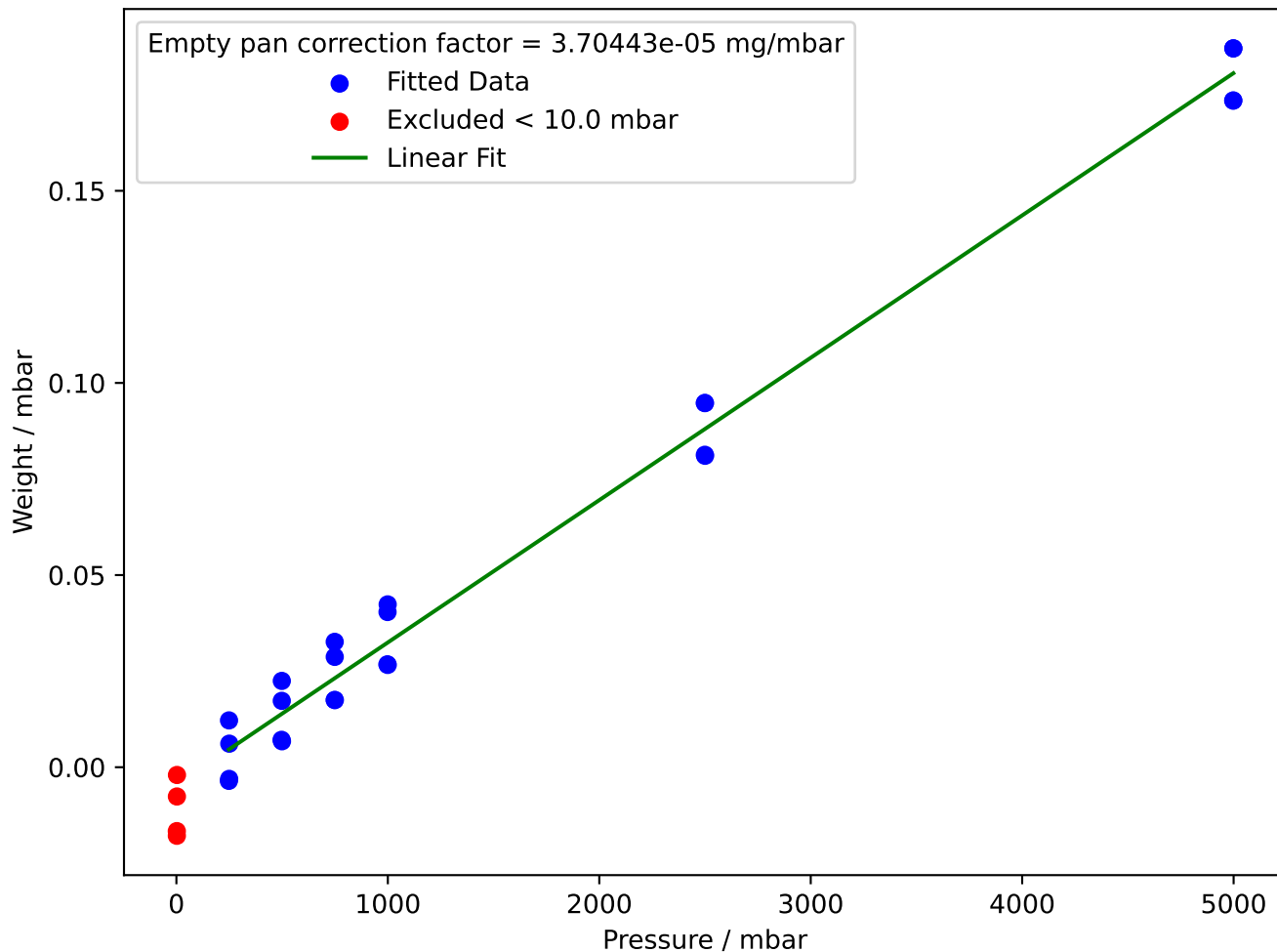

Supplement: Supplementary file 1 — Supplementary Material [file CSSC-18-e202501347-s001.zip › Microbalance_data_analysis/ZIF8-CO2/empty_pan_plot_30.0C.pdf]

# Absorbed gas at T = 30.0°C

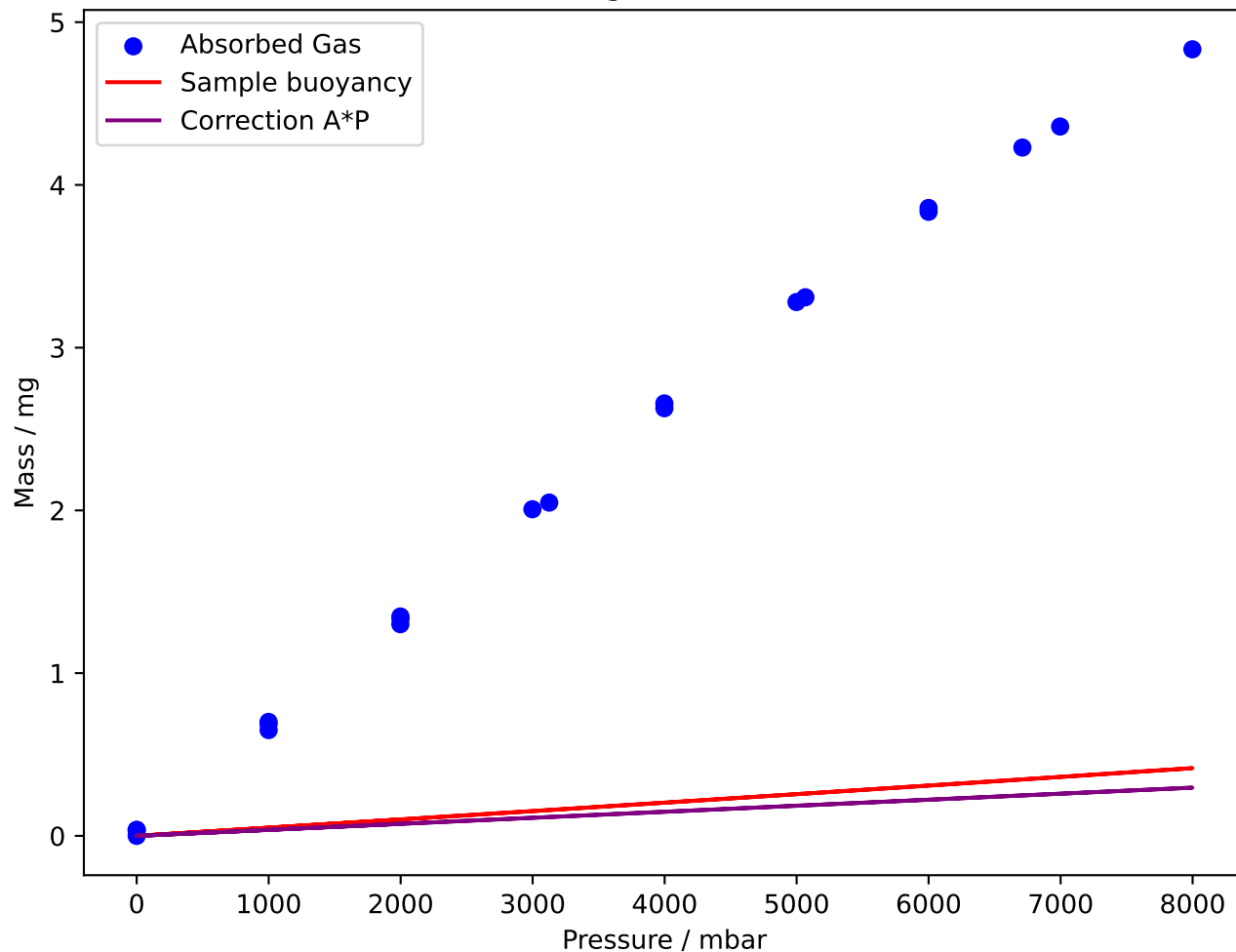

Supplement: Supplementary file 1 — Supplementary Material [file CSSC-18-e202501347-s001.zip › Microbalance_data_analysis/ZIF8-CO2/measurement_plot_30.0C.pdf]

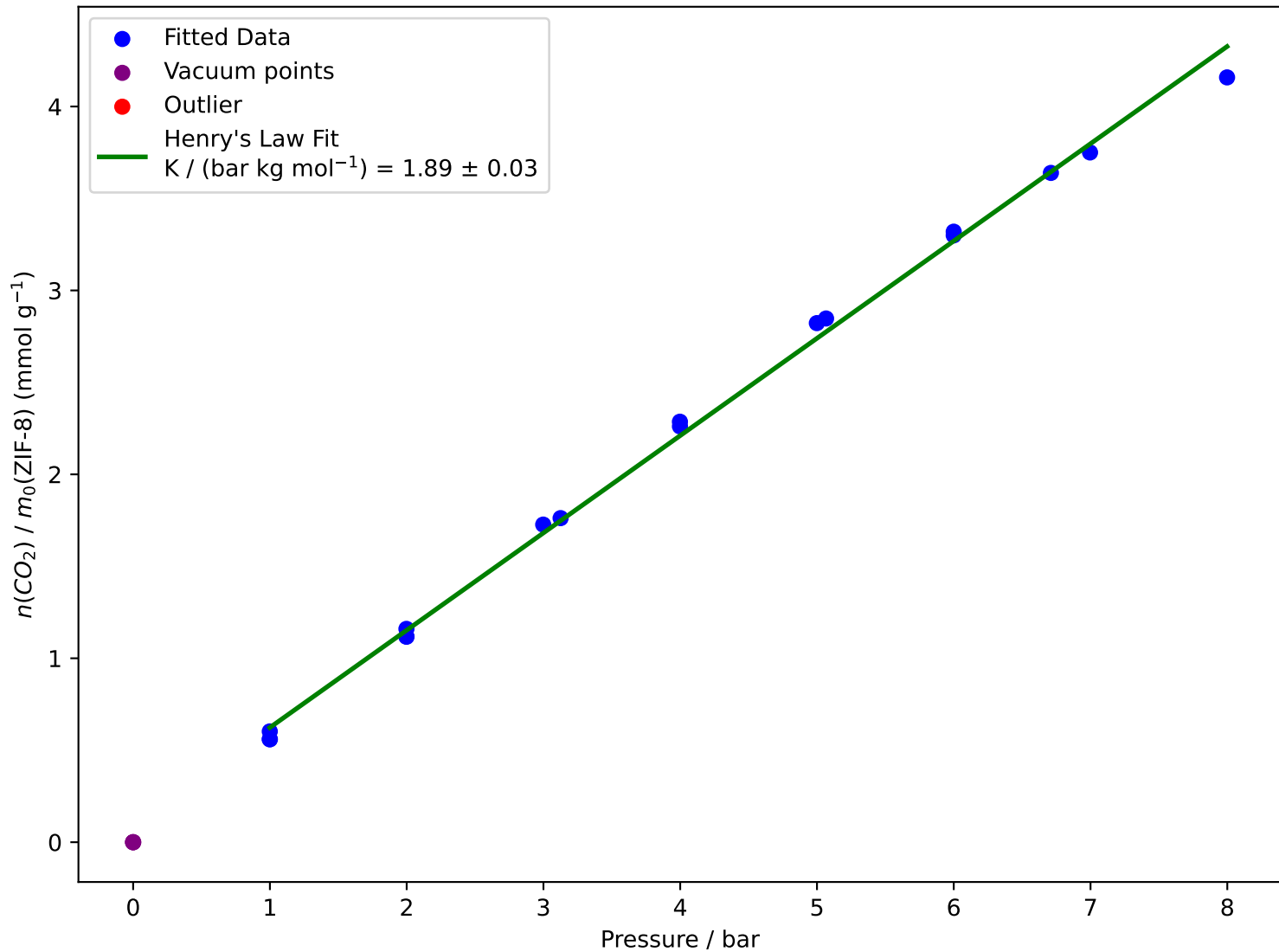

Supplement: Supplementary file 1 — Supplementary Material [file CSSC-18-e202501347-s001.zip › Microbalance_data_analysis/ZIF8-CO2/molality_fit_30C.pdf]
